# RNA-sequence analysis of gene expression from honeybees (*Apis mellifera*) infected with *Nosema ceranae*

**DOI:** 10.1371/journal.pone.0173438

**Published:** 2017-03-28

**Authors:** Bouabid Badaoui, André Fougeroux, Fabien Petit, Anna Anselmo, Chiara Gorni, Marco Cucurachi, Antonella Cersini, Anna Granato, Giusy Cardeti, Giovanni Formato, Franco Mutinelli, Elisabetta Giuffra, John L. Williams, Sara Botti

**Affiliations:** 1 Parco Tecnologico Padano - CERSA, Integrative Biology Group, Lodi, Italy; 2 Syngenta, Guyancourt, France; 3 RNAGRO, TOURS, France; 4 Istituto Zooprofilattico Sperimentale del Lazio e della Toscana, Roma, Italy; 5 Istituto Zooprofilattico Sperimentale delle Venezie, Legnaro, Padua, Italy; 6 Davies Research Centre, University of Adelaide, Roseworthy, South Australia, Australia; University of North Carolina at Greensboro, UNITED STATES

## Abstract

Honeybees (*Apis mellifera*) are constantly subjected to many biotic stressors including parasites. This study examined honeybees infected with *Nosema ceranae* (*N*. *ceranae*). *N*. *ceranae* infection increases the bees energy requirements and may contribute to their decreased survival. RNA-seq was used to investigate gene expression at days 5, 10 and 15 Post Infection (P.I) with *N*. *ceranae*. The expression levels of genes, isoforms, alternative transcription start sites (TSS) and differential promoter usage revealed a complex pattern of transcriptional and post-transcriptional gene regulation suggesting that bees use a range of tactics to cope with the stress of *N*. *ceranae* infection. *N*. *ceranae* infection may cause reduced immune function in the bees by: (i)disturbing the host amino acids metabolism (ii) down-regulating expression of antimicrobial peptides (iii) down-regulation of cuticle coatings and (iv) down-regulation of odorant binding proteins.

## Introduction

Honeybees (*Apis mellifera*), are critical for agricultural ecosystems, but are exposed to many biotic stressors including bacteria, viruses and fungi. *Nosema ceranae* (*N*. *ceranae*) is a microsporidian parasite which is one of the most common parasites of the honeybee. It infects the midgut [1] and has many adverse effects which reduce the health of individual honeybees [2–4] and of the colony [5–10]. *N*. *ceranae* infection is associated with several fitness related problems including deterioration of the gut epithelial cells [11,12], immune suppression [13] and energetic stress [14,15].Honeybees have developed many defense mechanisms to deal with parasite infection which include an antimicrobial secretion layer on the gut, and cellular and humoral immune mechanisms [16]. The honeybee humoral response has been shown to involve four antimicrobial peptides, abaecin, apidaecin, defensin and hymenoptaecin [17–20].

A draft *A*. *mellifera* genome sequence was published in 2004 [21] and an improved version of the genome sequencewas published in 2014 [22]. This sequence is valuable for the interpretation of RNA-seq data to study gene expression. RNA-seq may be used [23] to characterize the whole transcriptome including transcription start sites (TSS), splicing variants and differential promoter usage [24].

The molecular response of the honey bee to infection by *N*. *ceranae* has been investigated using RNA-seq, but the focus to date has been on cells of the midgut [25]. Digital gene expression has also been used to study the neurogenomic response to *N*. *ceranae* in the bee brain [26].The honey-bee nervous system, trachea and intestine interact to maintain the physiological homeostasis following damage resulting from infection; e.g. intestinal stem cells replace damaged intestinal epithelial cells [27]. Interestingly, the message for intestinal regeneration come not only from the damaged epithelial cells, but also from adjacent (trachea, muscles) and distant tissues, and the brain (reviewed in [28]).

In this study, RNA-seq was used to investigate gene expression, splice variants, TSSs and differential promoter usage in honeybees infected with *N*. *ceranae* at days 5, 10 and 15 P.I. The new version of the bee genome (Amel_4.5_scaffolds.fa) was used to annotate the genes. The study addressed the global transcriptome response of the bee to *N*. *ceranae* infection, not the response of specific tissues, and identified many transcripts and pathways involved in the interaction between the honeybee and the parasite.

## Material and methods

### *N*. *ceranae* spore preparation

*N*. *ceranae* spores were obtained according to Aufauvre et al. 2011 [25]. Briefly, forager bees were sampled at the hive entrance. The abdomens of 30 bees were triturated adding 30 ml distilled water. The homogenate was filtered and the resulting suspension was centrifuged for 6 minutes at 800g. The supernatant was removed and the spores resuspended in 3 ml distilled water. The spore concentration was determined by counting using a haemocytometer chamber. Amedian of 8.6 X 10^5^spores/ml was obtained.

### Inoculation of bees with *N*. *ceranae*and RNA extraction from honeybees

Frames of *Apis mellifera* capped brood containing "about-to-emerge" pupae were cut out from healthy colonies located in France. The hives were verified as *N*. *ceranae* free. Subsequently, the frame of pupae from each colony was divided into 2 equal parts, introduced into two plastic boxes and kept in an incubator at ~30°C with high humidity. The experiment was performed in the apiary of Luz Saint Sauveur located at 00° 00’ 50.4”O and 42° 51’ 57.6” N.

After about 2 days, the emerging bees were counted, and 30 bees were retained within each box. The two boxes were transferred to the inoculation chamber (25°C) with a 12 hour dark/light (12/12 d/l) cycle. The bees of the "inoculation box" were fed for two days with 2 ml of 66% sucrose solution (w/v) containing 86,000 *N*. *ceranae* spores per bee (total of 2.6X10^5^ spores per "inoculation box"). The bees of the “control box” were fed with 2 ml of a solution of 66% sucrose without *N*. *ceranae* spores for two days. Throughout the experiment, sucrose solution was supplemented only when the Pasteur pipettes in the boxes were completely empty. On days 5, 10 and 15, five bees per box were removed and ground in liquid nitrogen, transferred in micro-tube containing Trizol and stored at -80°C. The number of live/dead bees in both boxes was counted daily and the dead bees were carefully removed. Total RNA was extracted from three bees from each box and at each time point (5, 10 and 15 days post infection, P.I.) using TRIzol (Invitrogen Life Technologies, Milan-Italy) and RNeasy columns (Qiagen). RNA quality was assessed by microcapillary electrophoresis on an Agilent 2001 Bioanalyzer (Agilent Technologies) with RNA 6000 Nanochips. RNA was quantified by spectrophotometry (ND-1000; NanoDrop Technologies).

#### Control for *N*. *ceranae* infection

Presence of *N*. *ceranae* RNA in each sample was assessed by PCR [2] or qPCR [4,29]. Only samples from the "inoculation box" that were positive for *N*. *ceranae* RNA were used to create RNA sequencing libraries. To verify that the inoculation was successful, the 15 remaining bees after 14 days (P.I) were examined for *N*. *ceranae* spores by microscopy and the number of spores present recorded.

### Library preparation for RNA-seq

Eighteen libraries corresponding to 9 control and 9 infected samples at 5, 10 and 15 days P.I. (3 samples each time-point) were sequenced. TruSeq Sample Prep Kits (Illumina Inc., San Diego, CA) were used to create libraries for 1×100bp single-end sequencing on the Illumina HiSeq 2000 instrument. The sequences obtained were submitted to the Sequence Read Archive at NCBI (PRJNA378655).

### RNA-seq data analysis and functional analysis of differentially expressed genes between control and infected bees

RNA-seq data analysis was performed as reported by Badaoui et al [30]. Briefly, sequence visualization and statistical analyses were performed with FASTQC (http://www.bioinformatics.bbsrc.ac.uk/projects/fastqch). Reads were mapped to the latest version of the honeybee reference genome (Amel_4.5_scaffolds.fa) [22]) using TopHat v2.0.10 with default parameters [31]. Transcript assembly was performed using Cufflinks v2.2.0 [32]. Once the short read sequences were assembled, the output files were merged using the Cuffmerge [31] function and processed using Cuffcompare [32], along with a reference GTF annotation file downloaded from the beebase database (http://hymenopteragenome.org/beebase/). Differential expression analysis was performed using the Cuffdiff function [32]. Three comparisons of gene expression were made between the control and infected bees at days 5, 10 and 15 P.I. For all the analyses, a feature was considered significant if the false discovery rate (FDR) was less than 0.05. The differentially expressed genes at 5, 10 and 15 days P.I were mapped to pathways from the Kyoto Encyclopedia of Genes and Genomes (KEGG) database [33]. The statistical analyses for bee survival and hunger level were performed using the R software "stats" package.

### Proboscis Extension Reflex assay (PER) to assess hunger levels by sucrose uptake behavior

After day 14, eight bees from each box were captured individually with forceps and each bee was placed individually in a glass vial and chilled on ice for 1 min. The bees were fixed to a long plastic drinking straw with tape around the thorax. Testing began 1h17min after the last bees were attached to the straws. The antennae were touched with a droplet of sucrose and if a bee responded by fully extending the proboscis—a Proboscis Extension Response (PER)—was recorded. Each bee was assayed for PER with a concentration series of 0.1%, 0.3%, 1%, 3%, 10% and 30% sucrose solution. Desensitization with water was done between each sucrose concentration. The number of *N*. *ceranae* spores in each bee was determined under a microscope after the PER test.

## Results

### Honeybees infection with *N*. *ceranae*: Infection status, survival rates and hunger analysis

Infection with *N*. *ceranae* was assessed by PCR and qPCR: only three out of five bees sampled from the "inoculated box" at 5 day P.I. were positive for *N*. *ceranae*, while all 5 bees were positive at days10 and 15 P.I. *N*. *ceranae* was not detected in any bees from the "control box". After day14 P.I., 15 bees were examined for the presence of Nosema spores under the microscope and the number of spores recorded. All the inoculated bees were infected, with a mean of 5.0 x10^5^spores per bee, and all the control bees were free from any spore (S1 Table).

Survival of infected bees at day 5 P.I. was significantly decreased (p<0.1): 83% compared to 100% for the control bees (S1 Fig). However, the survival rates between the infected and control bees, were not statistically significant at days 10 and 15 P.I. Bees infected with *N*. *ceranae* had increased levels of hunger compared with controls, assessed by the Proboscis Extension Response and feeding experiments (S2 Fig).An increase in the Proboscis Extension Response was seen especially at intermediate and high sucrose concentrations; 10% of sucrose solution (p-value<0.1) and 30% (p-value<0.1).

### 1. Differentially expressed genes, isoforms and TSSs, and differential promoter usage in honeybees infected with *N*. *ceranae*

#### 1.1. RNA-seq reads features and statistics

The mean of the reads produced from the eighteen libraries was 13.8 M per library. The mapping rate was, on average, 70% except for one library which had a low mapping rate of 29% and which was discarded from further analyses (S2 Table).The number of expressed unique genes and isoforms in infected and non-infected bees were 15694and 33182, respectively. The most highly expressed genes, with Fragments Per Kilobase of exon per Million fragments mapped (FPKM) between 1000 and 10000, represented a small fraction of the total genes (0.9%). Genes expressed at a low level, with FPKM between 1 and 10, were considerably more abundant (30–32%). Genes with intermediate expression, FPKM values from 100 to 1000 were the most abundant (33–36%).

**Fig 1:**
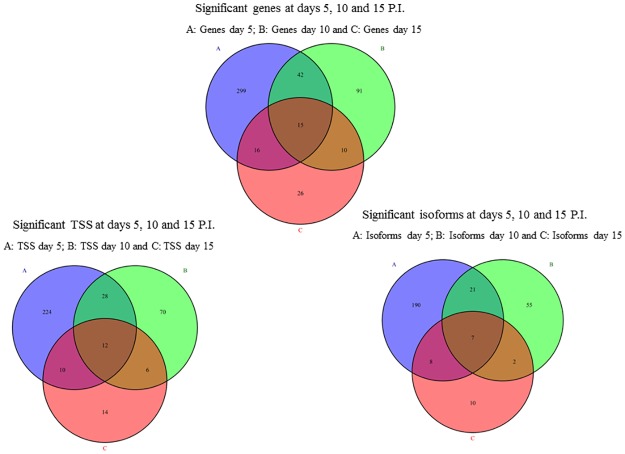
Venn diagram illustrating the significantly affected genes (A), isoforms (B) and TSS (C) between infections at days 5, 10 and 15 P.I.

The differentially expressed genes, isoforms, TSS and differential promoter usage between control and infected bees were respectively {372;226;274;8} at day 5 P.I., {158;85;116;8} at day 10 P.I. and {67;27;42;0} at day 15 P.I. (S3 Table).Among the differentially expressed genes, 53%, 50% and 30% were also differentially expressed at the isoform level, at days 5, 10 and 15 P.I., respectively(see Venn diagram, S3 Fig). The transcripts with altered forms at more than one time point of infection (days 5, 10 and 15 P.I.) were 15, 7 and 12 for the genes, isoforms and TSS, respectively (see Venn diagram in Fig 1).

#### 1.2. Transcriptional, post-transcriptional regulation and differential promoter usage in bees after infection with *N*. *ceranae*

Generally, transciptionally regulated isoforms haddifferent TSSs whilst post-transcriptionally regulated isoforms had the same TSSs (S4 Fig, [24]).The transcriptional start sites and isoforms of the top twenty most differentially expressed genes (S3 Table) at each time point (days 5, 10 and 15 P.I.) were investigated to assess if they were regulated at transcriptional or post-transcriptional levels, or both.

Three groups of genes were defined:

Group1:“Un-spliced and Transcriptionally regulated” genes that had one isoform and one TSS.This group had 9, 6 and 10 genesat days 5, 10 and 15 P.I. respectively (S5, S6 and S7 Figs (group 1)). Most of the genes in this group have a role in the immune response (e.g. GB40713, GB43007, and GB42217)
Group 2: “Spliced and Transcriptionally regulated” genes that had more than one isoform but only one TSS for each isoform. This group had 5, 8 and 3 genes at days 5, 10 and 15 P.I, respectively (S5, S6 and S7 Figs (group 2)).Many of these genes are involved in carbohydrate metabolism and ion transport (e.g. GB51580, GB45152, and GB46516)
Group 3: “Spliced and both Transcriptionally and Post-Transcriptionally regulated” genes with more than one isoform and more than one TSS. This group, contained genes with isoforms that are transcriptionally regulated and other isoforms which are post-transcriptionally regulated and had 6, 6 and 7 genes at days 5, 10 and 15 P.I, respectively (S5, S6 and S7 Figs (group 3)).These genes are involved, among other functions, in cuticle biosynthesis, trans-membrane transport activity and ATP binding (e.g. GB42218, GB45300 and GB47723).

Differential use of promoters between the control and infected bees was investigated as described by Mortazavi *et al* [24].This approach groups the primary transcripts of a gene based on the promoter used, followed by testing isoform abundance between the control and the infected bees. Eight genes showed different promoter use at day 5 P.I (*GB46442*, *GB41734*, *GB42277*, *GB44487*, *GB46749*, *GB42364*, *GB44323 and GB40485*), 8 genes at day 10 P.I (*GB40787*, *GB48346*, *GB47239*, *GB43964*, *GB40688*, *GB42265*, *GB55747 and GB55915*) and none at day 15 P.I (S3 Table).

#### 1.3. Gene expression characteristics at days 5, 10 and 15 P.I. with *N*. *ceranae*

**Fig 2:**
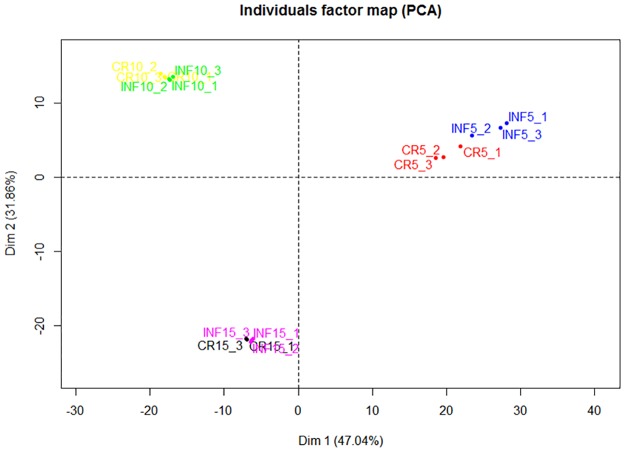
Principal component analysis of RNA-seq data. Gene expression changes were investigated at days 5, 10 and 15 P.I. in honeybees infected (INF) with *N*. *ceranae* or no treatment (CR). The PCA was performed using normalized RNA-Seq data of 675 genes differentially expressed in at least one pairwise comparison: control vs infection at day 5 10 P.I or 15 P.I.Clear differences were seen between samples collected at days 5, 10 and 15 suggesting that ageing of the bees has a larger effect on the pattern of gene expression pattern than infection status.

Principle component analysis (PCA) of the 675 genes which were differentially expressed in at least one pairwise comparison (control vs infection at day 5, control vs infection at day 10, control vs infections at day 15) (Fig 2) showed a clear difference between samples collected at days 5, 10 and 15. The control and infected samples showed the greatest difference at day 5 P.I while this difference was lower at day 10 P.I and no difference between controls and infected bees was found at day 15 P.I. This is reflected in the number of genes that were found to be differentially expressed between the infected bees and controls: 372, 158 and 93 at days 5, 10 and 15 P.I, respectively. The overlap between differentially expressed genes at different time points was greater than expected by chance for 5 days P.I versus 10 days P.I (p = 1.77e-52, hypergeometric test), 5 days P.I versus 15 days P.I (p = 6.55e-33) and10 days P.I versus 15 days P.I (p = 1.92e-33).

At day 5 PI there was a strong down-regulation of Royal jelly genes: *Mrjp1*, *Mrjp2*, *Mrjp3*, *Mrjp4* and *Mrjp6* (S4 Table) and a strong up-regulation of eight serine proteases: *SP22*, *SP40*,*SP44*, *SP17*, *SP18*, *SP35*, *SP36*, *SPH50* as well as the down-regulation of three other serine proteases: *SP34*, *SPH19* (serine protease homolog 19) and *SPH42* (serine protease homolog 42) (S3 and S4 Tables).

#### 1.4. Pathways affected during honeybee infection by *N*. *ceranae*

**Fig 3:**
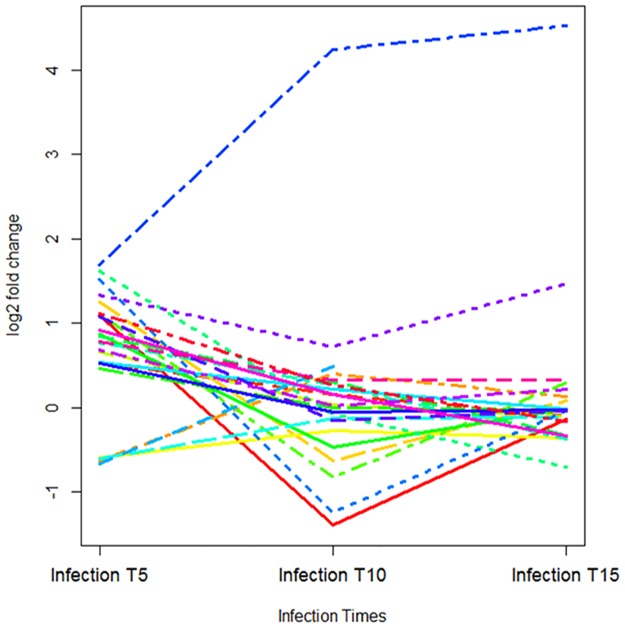
Expression profiles of the genes involved in the 'metabolic pathways'. The X axis shows the time points following infection (days 5, 10 and 15 P.I). On the Y axis, genes expression fold changes.

The most enriched KEGG pathway at all-time points (5, 10 and 15 days P.I) was 'metabolic pathways' (S5A Table, S8 Fig) which includes 'Energy metabolism', 'carbohydrate metabolism', 'amino acids metabolism' and 'lipid metabolism'. The number of genes differentially expressed between infected and control bees in the metabolic pathways decreases considerably from day 5 P.I (29 genes) to day 10 P.I (17 genes) and day 15 P.I (9 genes). The changes in the expression of 29 genes in this pathway were followed through the time-course of the challenge (Fig 3). Except the chitinase 5 (*Cht5*) gene expression of which increased from day 5 P.I to day 10 P.I and to day 15 P.I, expression of all the other genes decreased from day 5 P.I to days 10/15 P.I. Some genes reached their lowest level of expression at day 10 P.I (*GB44841*, *GB45654*, *GB50218*, *GB51814*, *GB42963*, *GB49240*, *GB55706*, *GB42434*), while others had the lowest level at day 15 P.I (*GB52756*, *GB51751*, *GB52724*, *GB42425*,*GB52923*, *GB48905*, *GB44367*, *GB40022*, *GB55705*) (Fig 3).The number of differentially expressed genes in the lysosome pathway, which is within the “metabolic pathways”, was also enriched at day 5 P.I (*GB53306*, *GB43825*, *GB51751*, *GB54097*, *GB55242*, *GB42616*) and day 10 P.I (*GB53306*, *GB43825*, *GB51751*, *GB53579*) although not at day 15 P.I. (S5A Table).

The KEGG annotation database contains little information on the bee signaling pathways. Therefore the differentially expressed genes were used to manually infer other pathways potentially modified during infection by *N*. *ceranae*. This identified the 'Immunity pathway', 'Trehalose transporter pathway', 'Cuticular pathway', 'Cytochrome pathway', 'Serine protease pathway' and 'Neurogenesis pathway' as being affected by the infection (S5B Table).

## Discussion

In this work we report differentially expressed genes, isoforms, TSS and promoter usage associated with infection of the bees with *N*. *ceranae*.

### Regulation complexity of differentially expressed genes in bees infected with *N*. *ceranae*

The most differentially expressed genes between uninfected and infected bees were classified into three groups according to their transcriptional/post-transcriptional regulation. The first two groups included genes involved in the innate immune response. These genes did not show any evidence of different isoforms and/or post-transcriptional regulation. Examples of these groups include: *GB48966*, a *homeotic protein caudal*, which operates post-embryonically in the intestine where it regulates antimicrobial peptide levels to maintain the normal gut flora [34]; *GB51833*, *Sodium dependent nutrient amino acid trasporter*, which contributes to the synthesis of catecholamines for sclerotization pathways involved incuticle colour, wound curing, and immune responses [35].

Post transcriptional regulation is responsible for expanding protein functional diversity and differs dependent on environmental conditions and developmental stage to expand phenotypic plasticity on the molecular level [36,37]. However, the selection of the incorrect splices sites may cause gene dysfunction [38]. The genes in these first two groups may be under strong selection pressure to prevent the emergence of new isoforms or alternative transcriptional regulation to avoid loss of function.

*N*. *ceranae* is amitochondriate, and so has high dependency on host ATP. The energy demands of the parasite increase the energy requirement for the bee, which is seen in the increased hunger of infected bees revealed by the PER test. GB41033, which is involved in the metabolism of the sugar, was highly expressed in infected bees, and is consistent with increased sugar metabolism associated with the higher energy requirement [11].*GB42217*, *acyl-CoA desaturase 1-like*, has a role in oxidation-reduction process following infection [39].

The third group includes genes mainly regulate biochemical and developmental processes, and includes receptors such as serpentine (*GB45151*) that regulate the expression of multiple classes of developmental genes [39, 40], SLC6 (GB51834) that transport a whole suite of molecules across cell membranes [41] and hedgehog (*GB45300*) which is required for normal regulation of cell proliferation and differentiation during embryonic stages [40].

This third group genes includes immune function genes and shows abundant examples of alternative splicing. There are many examples where the immune system has been shown to use diverse mechanisms of gene regulation to increase the versatility of response, including alternative splicing, different TSS and promoter usage [42,43], for example, in vertebrates alternative splicing of CD44, a protein involved in T cell homing, is crucial for T cell function [43].The transcription factors p63, p73 and p53, implicated in cell response to stress and development, and encodes multiple proteins as a result of post transcriptional regulation [42]. Diverse post-transcriptional regulation mechanisms seen for genes involved in bee immune response, cell signals and development may increase the immune repertoire to respond to infection.

Alternative promoter usage was found for 8 genes at days 5 and 10P.I in comparison with uninfected controls, but none were seen at day 15 P.I. This suggests that alternative promoter usage is important for bee-*N*. *ceranae* interaction as seen in previous studies where differential promoter usage has been associated with the diversity of gene expression pattern to deal with different pathogen challenges [44,45].That no alternative promoter usage was found at day 15 P.I and that a small number of differentially expressed genes is seen at this time point compared with time points 5 and 10 P.I. may suggest that the host and parasite are becoming adjusted to each other. It has been reported that genes with alternative promoters use are also more likely to be differentially expressed [46]. Genes related to human cancer which have altered expression have on average 2 promoters compared with an average of 1.5 promoters for the other human genes [47].

The overrepresentation of the splice variants, 30%-53% of genes between 5 and 10 days PI respectively of differentially expressed genes, seen following infection, suggests that extending protein diversity is important for bees to deal with the *N*. *ceranea* infection. Therefore, start site and post-transcriptional regulation is potentially important for tuning the response of the bee to *N*. *ceranea* infection, possibly by controlling the strength and the duration of the response [48]. This is in accordance with findings reported for Drosophila melanogaster [49] and mammals [50] where splice variants play an important role in dealing with infection. Interestingly, alternative TSSs have been associated with stable differences in behavior in honeybees [51].

Genes involved in the “metabolic pathways” (S5 Table) have higher expression at day 5 P.I than days 10 and 15 P.I. This suggests an increased energy demand placed on the bee by *N*. *ceranae* during early stages of the infection which is in agreement with the PER test responses.

### Pathways enriched in bees infected with *N*. *ceranae*

The global pathway 'metabolic pathways' showed a general increase in expression at day 5 P.I. Increased energy metabolism via carbohydrate catabolism is a key feature of bees infected with *N*. *ceranae*. Increased energy need and hence hunger was seen in the present study from the PER tests.*Cht5*, *GMCOX3*, *LOC55124* and *LOC408474* were the most highly expressed genes in this pathway at day 5 P.I with a log fold change greater than 1.5. *Cht5* and *GMCOX3* are involved in chitin metabolism and immune response respectively, and have previously been found to be modulated during honeybee infection with *N*. *cerania* [25]. While *GMCOX3* expression decreased after day 5 P.I, expression of *Cht5* continued to increase to reach log fold changes of 4.28 and 4.52 at days 5 and 10 P.I, respectively. *LOC55124* and *LOC408474* are involved in protein and lipid metabolism, respectively.

The lysosome pathway also plays an important role in bees infected with *N*. *ceranae* which is shown by the genes GB53306, GB43825, GB51751, GB54097, GB55242, and GB42616in this pathway having increased levels of expression in infected bees. Lysosome has been implicated in antifungal and antiviral activity [52]. An increased level of lysosome activity maybe a defense strategy used by the bee to digest the *N*. *ceranae* mycelia. Furthermore, the pathways: 'Immunity', 'Trehalose transporter', 'Cuticular', 'Cytochrome', 'Serine protease' and 'Neurogenesis' were modulated in infected bees compared with controls (S5B Table). These pathways have also been found to be regulated by bees in response to *N*. *ceranae*, *N*. *Microsporidia*, *E*.*coli and* Paenibacillus larva infections [25, 11, 53].

### Infection with *N*. *ceranae* reduces the bees energy reserves

Food quantity and quality is a key factor regulating the development of female larvae, either into a queen or a worker. Specifically, the Major Royal Jelly Proteins (MRJPs) are consumed to some extent by all larvae, but mainly by the larvae destine to become the adult queen [35].The Royal Jelly genes: *Mrjp1*, *Mrjp2*, *Mrjp3*, *Mrjp4* and *Mrjp6*were down regulated at day 5 P.I. The protein hexamerin (*HEX*) is accumulated by the bees to enhance their growth [54].Expression of this gene was not changed at day 5 P.I but was down-regulated at days 10 and 15 P.I. Down-regulation of *HEX110*, *HEX70b* and *HEX70c* has previously been reported in bees infected with *Paenibacillus larvae* and *chalkbrood fungus* [55, 56]. Neuropeptide Y (*NPY*), has been implicated in regulating food intake [57].The receptor for this neuropeptide was up-regulated following *N*. *ceranae* infection. Insulin growth factor binding protein was also up-regulated **(**S3 Table). Insulin signaling regulates nutritional balance [57]. Importantly, among the top pathways differentially expressed (up-regulated) at day 5 P.I, were 'Energy metabolism', 'lipid metabolism' and 'carbohydrate metabolism'. Therefore *N*. *ceranae* infection seems to affect metabolism and nutritional status of the infected bees. The increased transcription of four genes mapping to the trehalose transporter (*LOC413576*, *LOC726505*, *LOC724874* and *LOC413575)*, which constitute the main major carbohydrate energy storage molecule in insects, supports this observation.

Many studies have reported changes in nutrition [14,58] and increased feeding rate [59,60] in parasitized insects. In particular, studies of bees infected with *N*. *ceranae* have reported a higher sugar demand [11,14,60–64]. *N*. *ceranae* requires ATP to replicate within the honey bee mid-gut tissue [11,65] but being amitochondriate, has limited metabolic capacity and is unable to produce ATP.As a consequence it is completely dependent on host ATP [66,67]. *N*. *ceranae* therefore imposes an energetic demand on its host, which is consistent with the changes expression of metabolism related genes the increased hunger responses and lower survival seen in the present study.

### *N*. *ceranae* infection alters amino acid metabolism and suppresses the bee immune response

The amino acids metabolic pathway was modulated by *N*. *ceranae* infection. In a previous study, worker bees infected with *N*. *ceranae* were found to have altered amino acids titers in their haemolymph [68], which may affect the immune function [69–71]. Disturbance of protein metabolism may be a strategy adopted by *N*. *ceranae* to suppress the bee’s immune response [13] and promote its own survival. Bees infected with Varroa mite, show changes the expression of genes related to protein metabolism, including the down regulation of the Vg [72–74]. In the present study the expression of genes coding for the Vg, MRJP protein were found to be down-regulated.

Following infection by *N*. *ceranae*, a large number of genes coding for serine proteases were modulated (*SP17*, *SP18*, *SP22*, *SP34*, *SP35*, *SP36*, *SP40*, *SP44*, *SPH19*, *SPH42*,*SPH50*) at days 5, 10 and 15 P.I. The serine proteases are implicated in the immune response for Drosophila where 94 out of 201 serine protease genes are involved in diverse immune protease cascades [53].

Except SP36 and SPH42, all these genes showed alternative splicing and differential TSS (S3 Table) which might increase their expression plasticity and function. Activation of these molecules in the arthropod hemolymph is a central component of several immune responses, including the activation of antimicrobial peptide synthesis, and modulation of hemocyte function [75, 76].

Therefore, in addition to placing additional energy demands on the host *N*. *ceranae* also seems to affect the immune response of infected bees.

Honeybees have many immune pathways and defense mechanisms consisting of both cellular and humoral immune response [16] including antimicrobial peptide (AMPs) synthesis [77]. Honeybee humoral immunity involves four main antimicrobial peptides: abaecin, apidaecin, defensin and hymenoptaecin [20]. In the present study, apidaecin (*Apid1*, *Apid73*), defencin (*Def1*) and hymenoptaecin (*GB51223*) were strongly down-regulated at day 5 P.I compared with uninfected controls but not at days 10 and 15 P.I, except for the hymenoptaecin gene that was up-regulated at day 15 P.I. All these genes have at least two differentially expressed isoforms and TSS [78]. Different sequences of apidaecin arising from post translational regulation have been reported [79, 17], and Klaudiny et al [78] have reported a novel defensin isoform in honeybee. The finding reported here suggests that specific splice variation and TSS may occur in response to pathogens.

The egg yolk protein vitellogenin, which has been implicated in regulating the number of haemocytes [80] was significantly decreased following *N*. *ceranae* infection at day 5 P.I. Immune suppression has been reported following infection with by *N*. *ceranae* [13] and the Varroa mite [81].

Two genes coding for the major components of pathogen recognition (PGRPs) were modulated in this study: peptidoglycan recognition protein S1 (*PGRP-S1*) was down-regulated and peptidoglycan recognition protein S3 (*PGRP-S3*) was up-regulated. Honeybees have four PGRPs, PGRP-S1, PGRP-S2, PGRP-S3, and PGRP-LC [79], either act as recognition proteins, or can degrade bacterial cell wall through amidase activity [82, 83].Honeybees have much lower diversity of PGRP than Drosophila and Anopheles which have 13 and seven genes, respectively. This may limit the diversity of pathogens that bees can recognize compared with drosophila [79]. The present study identified that all the honeybee PGRPs were subjected to alternative splicing and TSS differentiation, which may be an evolutionary strategy to diversify the protein repertoire and overcome the limited number of PGRPs.

### Cuticular, olfactory and neuronal modifications during infection of bees with *N*. *ceranae*

Cuticle coatings are important to protect insects from pathogens and cuticle structure may be altered during infection [84]. In this work, seven cuticle genes (*LOC724464*, *CPR19*, *LOC725547*, *Cpap3-a*, *CPR24*, *CPR5* and *Cpap3-dC*) were down-regulated at day 5 P.I compared with controls and one additional gene (*CPR2*) was down-regulated at day 10 P.I. All these genes showed alternative splicing and differential TSS. The down-regulation of these cuticle genes maybe a strategy of the pathogen to improve its survival, by facilitating transfer among members of infected hive. Expression of the cuticle genes is associated with hygienic behavior [85, 86] which may also affect parasite transfer and survival.

Infection by *N*. *ceranae* may also change the behavior of bees by altering olfactory acuity. Three genes coding for (*Obp14*, *Obp18* and *Obp3*), were down-regulated at day 5 P.I. compared with controls, two (*obp3*, *obp17*) at day 10 P.I and two (*obp14*, *obp3*) at day 15 P.I. The odor binding proteins are not restricted to olfaction and they have other functions, including the adaptation to different environments [87]. Changes in expression of odor binding proteins following *N*. *ceranae* may change the behavioral or physiological responses of bees.

Transcripts involved in neurogenesis such as Slit genes, *LOC410555*, *LOC724772*, leucine-rich repeat neuronal protein 3 (*LOC724187*), neuropeptide and neurotransmitter transporter 8, were modulated at day 5 P.I but not at days 10 and 15 P.I. Slitis part of a large network of genes involved in tissue regeneration and has been shown to be down regulated following infection by Nosema [11]. Changes in expression of genes involved in neuronal development have also been reported following infestation with the Varroa mite [88].Therefore bee parasites may affect behavior either to enhance their survival or to promote the spread within the colony.

## Conclusions

*N*. *ceranae* infection results in the regulation of gene function, both at the level of expression and through changes in promoter usage and post translational variation. The effects of *N*. *ceranae* infection are seen on expression of genes involved in three key processes:1) energetic stress which is reflected in elevated hunger levels, 2) the modulation of the metabolic pathways, particularly immune function and 3) changes in behavior mediated through altered olfactory or hygiene related gene expression. These observed changes could be associated with the host responding to clear the infection, and with the infecting *N*.*Ceranea* altering host gene expression to promote its survival.

## Supporting information

S1 Table Counts of *N*. *ceranae*in control and infected bees with N.Ceranea. 100% of the inoculated bees used for the bioassay were infected by Nosema while no control bees were infected.
(PDF)

S2 Table (i) RNA-seq reads numbers generated per sample, (ii) RNA-Seq reads number and percentage mapped to the honeybee genome and (iii) the number and percentage of RNA-seq reads that mapped to more than one site in the genome. (PDF)

S3 Table Differentially expressed genes, isoforms, TSS and differential promoter usage during infection with *N*.*Ceranea* at days 5, 10 and 15 P.I. (XLS)

S4 Table Genes differentially expressed (|FC|> = 1.5) between infected and control bees at days 5, 10 and 15 P.I. (PDF)

S5 Table A. mapping of the differentially expressed genes at days 5, 10 and 15 P.I. to pathways from the Kyoto Encyclopedia of Genes and Genomes (KEGG) database. The top pathways in which at least 4 genes were involved were selected. B. Manually inferred pathways using the differentially expressed genes at days 5, 10 and 15 P.I.
(ODS)

S1 Fig Survival rates of control and infected bees with *N*. *ceranae* up to 14 days Post Infection (P.I.). (PDF)

S2 Fig Percentage of bees triggering a Proboscis Extension Response at different concentration sucrose solution (30%,10%, 3%, 1%, 0.3% and 0.1%). (PDF)

S3 Fig Venn diagram illustrating the significantly affected (A) genes (B), isoforms and (C) TSSs found in bees infected with *N*. *ceranae* at days 5, 10 and 15 P.I. (PDF)

S4 Fig Cufflinks approach for estimating transcriptional and post-transcriptional regulatory effects on overall transcript output. Let’s consider a transcript with two isoforms (A, B). When the abundance of isoforms A, B and C are grouped by TSS, the changes in the relative abundance of the TSS groups indicate transcriptional regulation (A+B vs. C). Post-transcriptional effects are observed as changes in the levels of the isoforms in a single TSS group (A vs. B) (Adapted from Trapnell et al. 2012).
(PDF)

S5 Fig Transcriptional/Post-transcriptional regulation of the top twenty significant genes following the infection of bees with *N*. *ceranae* at day 5 P.I. (Group 1) Un-spliced and transcriptionally regulated genes,(Group 2) spliced and post-transcriptionally regulated genes and(Group 3) spliced and both transcriptionally and post-transcriptionally regulated genes. For each transcript, the “XLOC”, “TSS” and “TCONS” suffixes correspond to the genes, TSSs and isoforms, respectively. Differentially expressed isoforms with different TSSs are transcriptionally regulated, while isoforms with the same TSS are regulated at the post-transcriptional level.
(PDF)

S6 Fig Transcriptional/Post-transcriptional regulation of the top twenty significant genes following the infection of bees with *N*. *ceranae* at day 10 P.I. (Group 1) Un-spliced and transcriptionally regulated genes,(Group 2) spliced and post-transcriptionally regulated genes and(Group 3) spliced and both transcriptionally and post-transcriptionally regulated genes. For each transcript,the “XLOC”, “TSS” and “TCONS” suffixes correspond to the genes, TSSs and isoforms, respectively. Differentially expressed isoforms with different TSSs are transcriptionally regulated, while isoforms with the same TSS are regulated at the post-transcriptional level.
(PDF)

S7 Fig Transcriptional/Post-transcriptional regulation of the top twenty significant genes following the infection of bees with *N*. *ceranae* at day 15 P.I. (Group 1) Un-spliced and transcriptionally regulated genes,(Group 2) spliced and post-transcriptionally regulated genes and(Group 3) spliced and both transcriptionally and post-transcriptionally regulated genes. For each transcript, the “XLOC”, “TSS” and “TCONS” suffixes correspond to the genes, TSSs and isoforms, respectively. Differentially expressed isoforms with different TSSs are transcriptionally regulated, while isoforms with the same TSS are regulated at the post-transcriptional level.
(PDF)

S8 Fig The 'metabolic pathways' enriched following the infection of bees with *N*.*Ceranea*. This global pathway includes mainly 'energy metabolism', 'carbohydrate metabolism', 'amino acids metabolism' and 'lipid metabolism' related genes.
(TIF)
