# Supplementary material for: RNA-sequence analysis of gene expression from honeybees (Apis mellifera) infected with Nosema ceranae
Source: PLoS One. 2017 Mar 28;12(3):e0173438. doi: 10.1371/journal.pone.0173438 (PMC5370102; doi:10.1371/journal.pone.0173438)
Supplement: S1 Table — 100% of the inoculated bees used for the bioassay were infected by Nosema while no control bees were infected. (PDF) [file pone.0173438.s001.pdf]

Table 1S: Spores microscopic counts of the control and infected bees with *N.Ceranea*. 100% of the inoculated bees used for the bioassay were infected by *N.Ceranea* while no control bees were infected .

Inoculated Bees	Spores/bee	Non inoculated bees	Spores/bee
**I1**	650000	C1	0
**I2**	270000	C2	0
**I3**	300000	C3	0
**I4**	440000	C4	0
**I5**	400000	C5	0
**I6**	580000	C6	0
**I7**	660000	C7	0
**I8**	720000	C8	0
