# Supplementary material for: RNA-sequence analysis of gene expression from honeybees (Apis mellifera) infected with Nosema ceranae
Source: PLoS One. 2017 Mar 28;12(3):e0173438. doi: 10.1371/journal.pone.0173438 (PMC5370102; doi:10.1371/journal.pone.0173438)
Supplement: S2 Table — (PDF) [file pone.0173438.s002.pdf]

Table S2. This table reports the (i) RNA-seq reads numbers generated per sample, (ii) RNA-Seq reads number and percentage mapped to the honeybee genome and (iii) the number and percentage of RNA-seq reads that mapped to more than one site in the genome.

Library	Reads number	Mapped reads	Mapped reads percentage	Multiple alignement	Multiple alignement percentage
5	13589421	9842477	72.4	477985	4.9
6	17581972	12675532	72.1	649674	5.1
7	15069458	10868939	72.1	526864	4.8
10	14220656	10283879	72.3	495547	4.8
11	19241017	13842540	71.9	669457	4.8
88	16585379	11959655	72.1	582142	4.9
15	20673693	15062160	72.9	693019	4.6
17	12172835	8696971	71.4	389288	4.5
18	14139277	10121418	71.6	453032	4.5
20	18816187	13555249	72	614301	4.5
22	17267609	12319410	71.3	539503	4.4
23	11064580	7776288	70.3	363532	4.7
24	9436576	2768575	29.3	99515	3.6
25	7906084	5700114	72.1	227429	4
26	9129226	6635306	72.7	267321	4
29	14179870	9819085	69.2	385357	3.9
30	9059013	6233810	68.8	253383	4.1
31	8975729	6362332	70.9	251555	4
**Mean**	13839365.67				
