# Supplementary material for: RNA-sequence analysis of gene expression from honeybees (Apis mellifera) infected with Nosema ceranae
Source: PLoS One. 2017 Mar 28;12(3):e0173438. doi: 10.1371/journal.pone.0173438 (PMC5370102; doi:10.1371/journal.pone.0173438)
Supplement: S4 Table — (PDF) [file pone.0173438.s004.pdf]

Table S4. Genes differentially expressed and with a |log2FC|>=1.5 between infected and control bees at days 5, 10 and 15 P.I.

Infection at day 5 P.I

beebase	gene_entry	gene_symbol	value_1	value_2	log2.fold_change.	q_value
GB41854	NA	NA	76.6849	3.88194	-4.30409	0.0027
GB47444,GB47445,GB47446	NA	NA	125.598	8.47003	-3.8903	0.0027
GB55211	100578601	LOC100578601	5.49417	0.379153	-3.85705	0.015151
GB55204	406121	Mrjp3	27.2866	2.55068	-3.41924	0.0027
GB44487,GB44488	NA	NA	62.4839	6.16819	-3.34057	0.0027
GB52201	NA	NA	30.5521	3.05917	-3.32006	0.0027
GB55205	406090	Mrjp1	186.858	19.5664	-3.25549	0.0027
GB55212	406091	Mrjp2	16.4946	2.02161	-3.02842	0.0027
GB42624	726629	LOC726629	3.31132	0.630704	-2.39237	0.008905
GB45796	727045	LOC727045	3.62661	0.72631	-2.31997	0.006944
GB55206	406133	Mrjp4	35.7809	7.32547	-2.2882	0.0027
GB42410	100578816	LOC100578816	140.364	36.1526	-1.957	0.0027
GB41110	727546	LOC727546	5.7792	1.59877	-1.85391	0.033142
GB55207	406149	Mrjp6	239.93	73.2856	-1.71101	0.0027
GB53407	100578345	LOC100578345	5.32804	1.70831	-1.64104	0.0027
GB48862	NA	NA	95.7405	32.9357	-1.53948	0.0027
GB48407	100578341	LOC100578341	62.4144	178.887	1.5191	0.0027
GB42425	551224	LOC551224	7.02848	20.1984	1.52296	0.0027
GB46286	551524	LOC551524	439.029	1264	1.52561	0.0027
GB47933	408534	SP44	77.6835	225.792	1.53931	0.0027
GB40136	NA	NA	334.003	971.866	1.5409	0.0027
GB44099,GB44100	NA	NA	1299.04	3855.68	1.56954	0.0027
GB43739	551327	LOC551327	202.185	618.118	1.61221	0.0027
GB47239,GB47240	NA	NA	17.8726	54.6663	1.6129	0.0027
GB51814	410747	GMCOX3	29.6173	90.6521	1.6139	0.006944
GB41033	726505	LOC726505	3.81569	11.8211	1.63135	0.0027
GB40284	725159	CYP6AS10	29.7634	93.2301	1.64726	0.0027
GB53141	726980	LOC726980	47.9743	151.823	1.66206	0.0027
GB53565	551600	Cht5	6.07495	19.5959	1.68961	0.0027
GB48468	100576136	LOC100576136	0.649234	2.1473	1.72571	0.004902
GB42426	551180	LOC551180	205.052	681.341	1.73239	0.0027
GB43780	NA	NA	0.370933	1.23564	1.73603	0.040768
GB42619,GB42620	NA	NA	0.731239	2.47007	1.75614	0.0027
GB40285	NA	NA	10.5197	35.7862	1.7663	0.0027
GB42427	409619	LOC409619	19.9357	69.0266	1.7918	0.031665
GB52186	409626	SP40	51.3681	178.501	1.79699	0.0027
GB49796	100578144	LOC100578144	9.11783	31.7152	1.79841	0.0027
GB48738	100578535	LOC100578535	5.70234	19.9601	1.80749	0.0027
GB50467,GB50468,GB50469	NA	NA	4.90113	17.3312	1.82219	0.0027
GB48656	412969	LOC412969	0.726178	2.62339	1.85303	0.0027
GB53306	408992	LOC408992	3.94298	14.8878	1.91677	0.0027
GB41760	411353	LOC411353	149.538	569.912	1.93023	0.0027
GB50026	725250	LOC725250	167.36	651.534	1.96088	0.0027
GB40836	100576126	LOC100576126	1.12209	4.37056	1.96163	0.0027
GB44805	NA	NA	2.01814	8.03765	1.99375	0.006944

GB48079	413645	SP22	268.83	1091.54	2.0216	0.0027
GB40688,GB40689,GB40690	NA	NA	20.2506	83.2232	2.03902	0.0027
GB54417	552158	LOC552158	0.345905	1.51946	2.13511	0.030258
GB48109	409162	LOC409162	5.70332	25.3776	2.15369	0.0027
GB51436	727243	LOC727243	9.66224	46.2929	2.26036	0.0027
GB46103	100577161	LOC100577161	4.74528	27.7755	2.54925	0.0027
GB43310	724312	LOC724312	4.02641	25.1121	2.64082	0.0027
GB43971	NA	NA	2.14297	21.5832	3.33223	0.043043
GB44323	NA	NA	2.58988	122.248	5.56078	0.0027
GB40485,GB40486	NA	NA	0.748228	158.239	7.72441	0.0027
GB41324	NA	NA	0	2.2721	Inf	0.043043
GB45460	NA	NA	0	2.76827	Inf	0.043043
GB47197	NA	NA	0	3.11074	Inf	0.0027
GB51350	NA	NA	0	2.62442	Inf	0.0027
GB42029	NA	NA	4.79258	0	#NOME?	0.041445

Infection at day 10 P.I

GB51799	412203	bw	3.40282	0.267351	-3.66992	0.005342
GB40787	NA	NA	42.1106	3.33419	-3.65877	0.005342
GB55915	NA	NA	80.6107	6.95179	-3.53551	0.005342
GB44729	NA	NA	76.9343	6.94132	-3.47035	0.005342
GB40688,GB40689	NA	NA	42.3555	3.94965	-3.42275	0.005342
GB47239,GB47240	NA	NA	113.296	16.1606	-2.80954	0.005342
GB47409,GB47410	NA	NA	82.8107	12.7088	-2.70399	0.005342
GB52325	724561	LOC724561	12.1301	2.04873	-2.56579	0.005342
GB52201	NA	NA	22.3309	3.92871	-2.50691	0.038249
GB53756	410928	Est-6	4.43434	0.858745	-2.36842	0.005342
GB48510	406095	SP34	6.41499	1.31031	-2.29154	0.005342
GB46180,GB46181	NA	NA	47.7562	9.88127	-2.27292	0.005342
GB40421	NA	NA	4.60838	0.970343	-2.24769	0.005342
GB40836	100576126	LOC100576126	5.00452	1.17332	-2.09263	0.005342
GB42493	724749	LOC724749	13.5694	3.46659	-1.96876	0.017033
NA	NA	NA	66.4905	17.4395	-1.93079	0.005342
GB51901	410976	LOC410976	1.63544	0.513813	-1.67037	0.009713
GB50439	725547	LOC725547	41.1675	12.9732	-1.66598	0.005342
GB53114	100037419	Apd-3	5649.27	1816.44	-1.63695	0.005342
GB53371	677671	Obp3	323.422	104.144	-1.63483	0.005342
GB48861	552685	LOC552685	286.083	93.1308	-1.6191	0.005342
GB47563,GB47564	NA	NA	499.865	170.124	-1.55495	0.005342
GB51670	724225	LOC724225	0.975594	2.81318	1.52785	0.005342
GB53576	406093	LOC406093	1804.03	5454.04	1.5961	0.005342
GB48020	552041	LOC552041	665.934	2166.73	1.70207	0.013458
GB51446	727367	LOC727367	9.78466	31.8617	1.70323	0.013458
GB42217	727333	LOC727333	50.563	181.817	1.84633	0.005342
GB51216,GB51218	NA	NA	9.92476	42.0104	2.08164	0.005342
GB51383	550965	CYP6AR1	1.49915	6.47664	2.1111	0.005342
GB47713,GB47714	NA	NA	1.52638	8.18931	2.42362	0.023045
GB43688	100578392	LOC100578392	2.22249	14.6175	2.71745	0.005342
GB43972	NA	NA	1.98316	15.4163	2.95858	0.005342
GB40526	NA	NA	2.68757	34.9612	3.70138	0.005342

GB53565	551600	Cht5	5.80236	109.715	4.24098	0.005342
GB49819	100577298	LOC100577298	0	1.96962	Inf	0.005342
GB51270	NA	NA	0	1.22817	Inf	0.009713
GB51530	NA	NA	0	18.4732	Inf	0.005342
GB54889	NA	NA	0	8.71965	Inf	0.005342
GB40038	552136	LOC552136	2.05376	0	#NOME?	0.005342
GB40247	NA	NA	4.57908	0	#NOME?	0.009713
**Infection at day 15 P.I**						
GB45090	NA	NA	66.678	2.24217	-4.89424	0.009076
GB52678,GB52679	NA	NA	83.4436	7.03232	-3.56873	0.009076
GB55580	NA	NA	42.7855	4.28644	-3.31927	0.009076
GB52063,GB52064,GB52065	NA	NA	43.2203	5.57822	-2.95383	0.009076
GB54883,GB54884	NA	NA	29.8319	5.1127	-2.5447	0.009076
GB40421	NA	NA	7.67151	1.74808	-2.13374	0.016227
GB53888	100578347	LOC100578347	73.2394	18.9526	-1.95022	0.009076
GB54144	410515	LOC410515	2.20254	0.570675	-1.94843	0.009076
GB53371	677671	Obp3	268.155	89.0439	-1.59048	0.009076
GB40217	413064	LOC413064	11.808	4.02591	-1.55238	0.009076
GB46223	677673	Obp14	95.3043	32.6127	-1.54711	0.009076
GB49544	406088	Vg	39.0872	13.4098	-1.54342	0.009076
GB54097	494509	Mvl	69.0788	221.941	1.68386	0.009076
GB51306	406115	Apid73	3.77587	13.1428	1.79939	0.009076
GB54796	724934	PHDP	3.38972	14.2688	2.07362	0.009076
GB53860	724644	LOC724644	12.4182	56.5899	2.18809	0.02295
GB47318	NA	NA	343.992	1620.65	2.23613	0.02975
GB44834	NA	NA	2.32407	12.2045	2.3927	0.009076
GB43690	727344	LOC727344	10.0711	57.8573	2.52228	0.009076
GB51436	727243	LOC727243	1.38341	9.10756	2.71884	0.047449
GB51383	550965	CYP6AR1	0.653766	4.83468	2.88657	0.036182
GB41286,GB41287	NA	NA	24.1131	183.965	2.93154	0.009076
GB42542,GB42543	NA	NA	1.96848	15.0738	2.93689	0.009076
GB51223	406142	LOC406142	89.0778	796.656	3.16082	0.009076
GB43931	NA	NA	1.39159	18.1945	3.7087	0.02295
GB56026,GB56027	NA	NA	4.09927	55.1184	3.7491	0.009076
GB42218	100576797	LOC100576797	4.40475	59.6944	3.76046	0.009076
GB48011	NA	NA	1.03607	17.8417	4.10607	0.009076
GB53565	551600	Cht5	10.0844	231.375	4.52003	0.009076
GB54277	NA	NA	2.26576	71.3313	4.97647	0.009076
GB44729	NA	NA	4.03348	151.685	5.23291	0.009076
GB52201	NA	NA	1.62214	109.154	6.07232	0.009076
GB54564	NA	NA	0	2.52129	Inf	0.009076
NA	NA	NA	0	2.50895	Inf	0.009076
