# Supplementary material for: RNA-sequence analysis of gene expression from honeybees (Apis mellifera) infected with Nosema ceranae
Source: PLoS One. 2017 Mar 28;12(3):e0173438. doi: 10.1371/journal.pone.0173438 (PMC5370102; doi:10.1371/journal.pone.0173438)
Supplement: S1 Fig — (PDF) [file pone.0173438.s006.pdf]

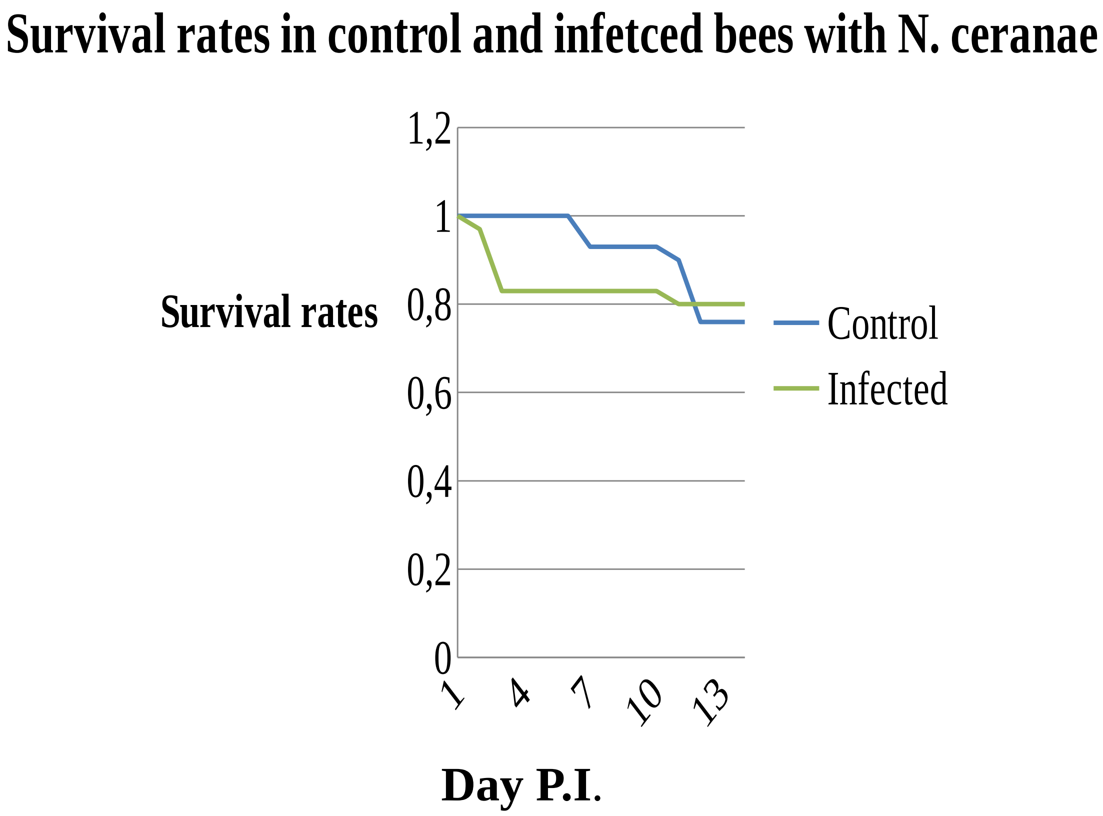


**Figure S1**. Survival rates of control and infected bees with *N. ceranae* during 14 days Post Infection (P.I.).
