# Supplementary material for: RNA-sequence analysis of gene expression from honeybees (Apis mellifera) infected with Nosema ceranae
Source: PLoS One. 2017 Mar 28;12(3):e0173438. doi: 10.1371/journal.pone.0173438 (PMC5370102; doi:10.1371/journal.pone.0173438)
Supplement: S2 Fig — (PDF) [file pone.0173438.s007.pdf]

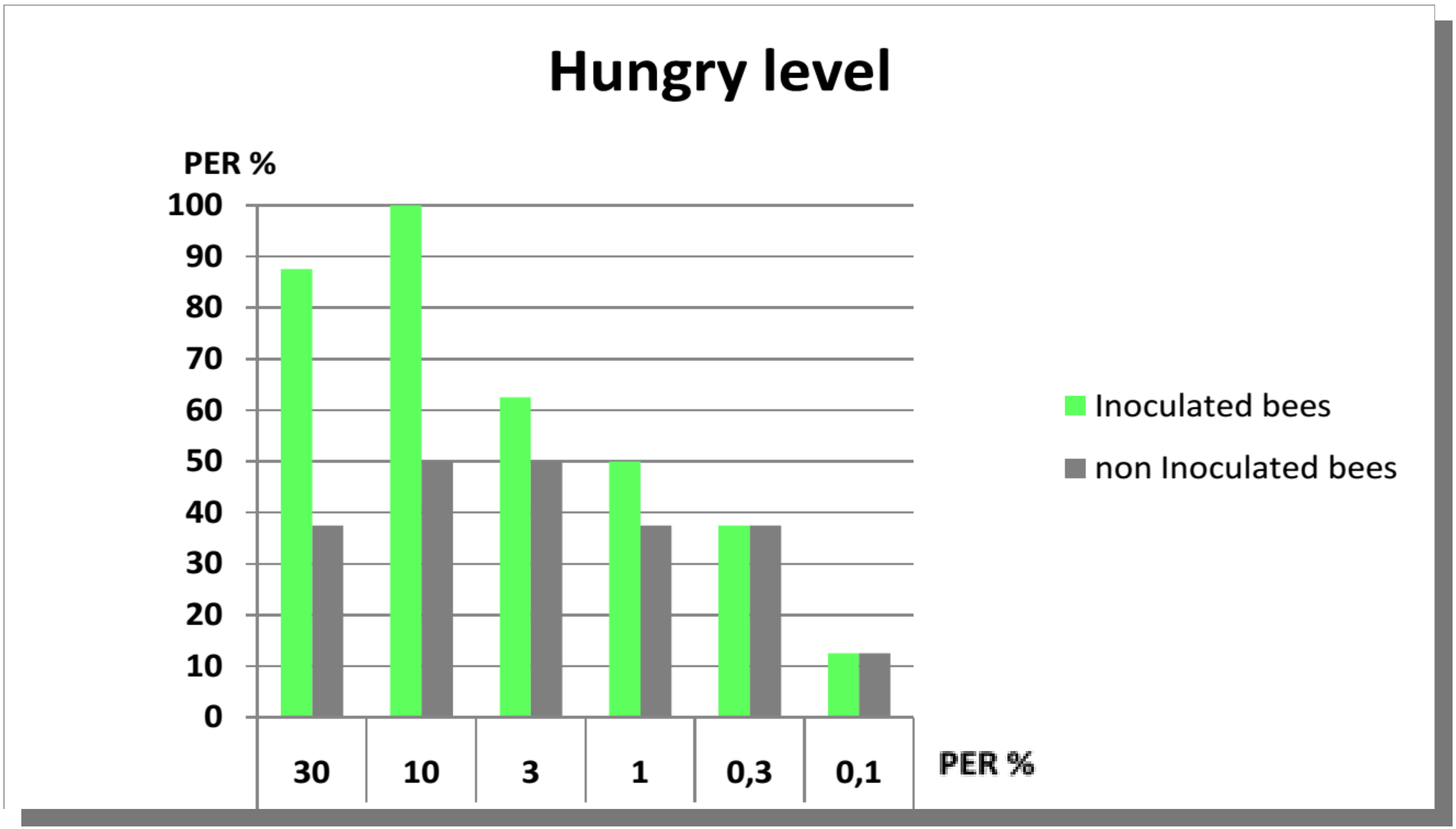

Figure S1. Percentage of bees triggering a Proboscis Extension Response at different concentration sucrose solution (30%,10%, 3%, 1%, 03% and 0,1%).
