# Supplementary material for: RNA-sequence analysis of gene expression from honeybees (Apis mellifera) infected with Nosema ceranae
Source: PLoS One. 2017 Mar 28;12(3):e0173438. doi: 10.1371/journal.pone.0173438 (PMC5370102; doi:10.1371/journal.pone.0173438)
Supplement: S3 Fig — (PDF) [file pone.0173438.s008.pdf]

Significant transcripts at day 5

A: Genes; B: Isoforms and C:TSS

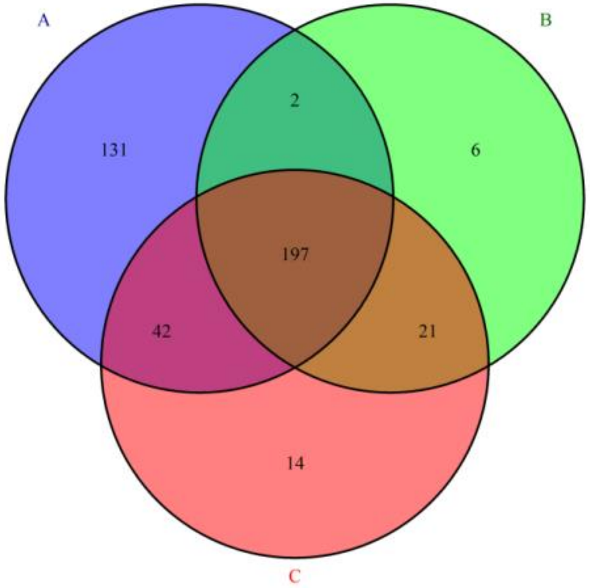

Significant transcripts at day 10

A: Genes; B: Isoforms and C:TSS

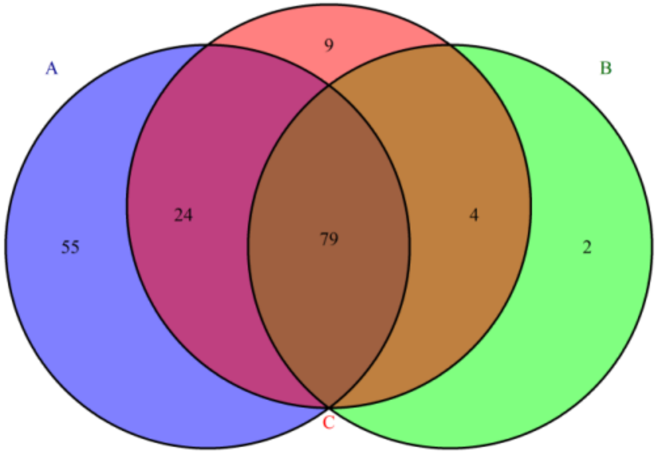

Significant transcripts at day 15

A: Genes; B: Isoforms and C:TSS

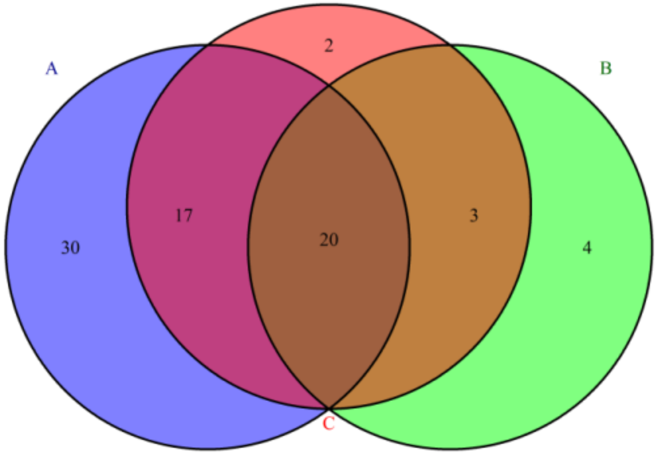

Figure S3. Venn diagram illustrating the significantly affected genes (A), isoforms (B) and TSSs (C) found in bees infected with *N.Ceranea* at days 5, 10 and 15.
