# Supplementary material for: RNA-sequence analysis of gene expression from honeybees (Apis mellifera) infected with Nosema ceranae
Source: PLoS One. 2017 Mar 28;12(3):e0173438. doi: 10.1371/journal.pone.0173438 (PMC5370102; doi:10.1371/journal.pone.0173438)
Supplement: S4 Fig — When the abundance of isoforms A, B and C are grouped by TSS, the changes in the relative abundance of the TSS groups indicate transcriptional regulation (A+B vs. C). Post-transcriptional effects are observed as changes in the levels of the isoforms in a single TSS group (A vs. B) (Adapted from Trapnell et al. 2012). (PDF) [file pone.0173438.s009.pdf]

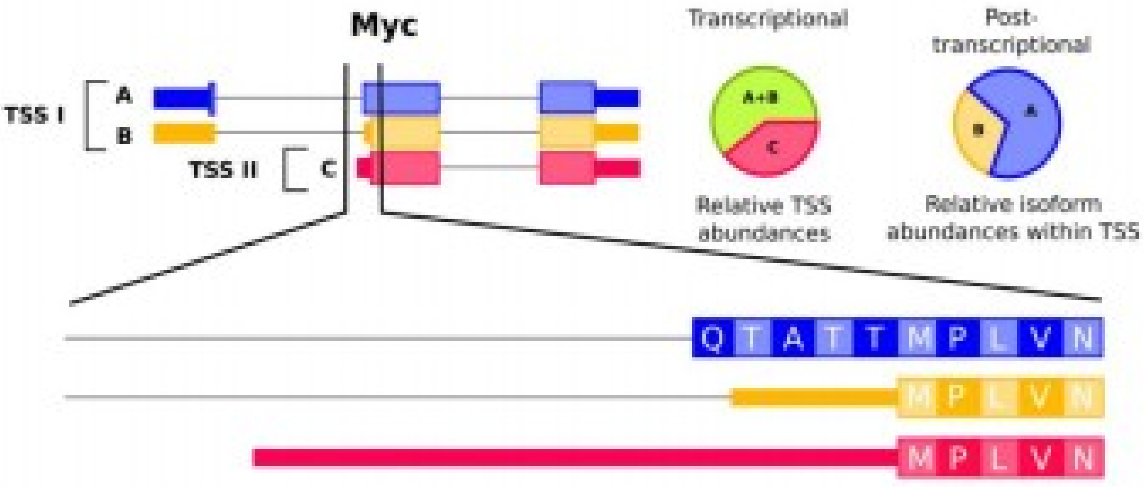

Figure S5. Cufflinks approaches for estimating transcriptional and post-transcriptional regulatory effects on overall transcript output. Let's consider a transcript with two isoforms (A, B). When the abundance of isoforms A, B and C are grouped by TSS, the changes in the relative abundance of the TSS groups indicate transcriptional regulation (A+B *vs*. C). Post-transcriptional effects are observed as changes in the levels of the isoforms in a single TSS group (A *vs*. B) (Adapted from Trapnell *et al*. 2012).
