# Supplementary material for: RNA-sequence analysis of gene expression from honeybees (Apis mellifera) infected with Nosema ceranae
Source: PLoS One. 2017 Mar 28;12(3):e0173438. doi: 10.1371/journal.pone.0173438 (PMC5370102; doi:10.1371/journal.pone.0173438)
Supplement: S5 Fig — (Group 1) Un-spliced and transcriptionally regulated genes,(Group 2) spliced and post-transcriptionally regulated genes and(Group 3) spliced and both transcriptionally and post-transcriptionally regulated genes. For each transcript, the “XLOC”, “TSS” and “TCONS” suffixes correspond to the genes, TSSs and isoforms, respectively. Differentially expressed isoforms with different TSSs are transcriptionally regulated, while isoforms with the same TSS are regulated at the post-transcriptional level. (PDF) [file pone.0173438.s010.pdf]

Group 1: un-spliced and transcriptionally regulated genes

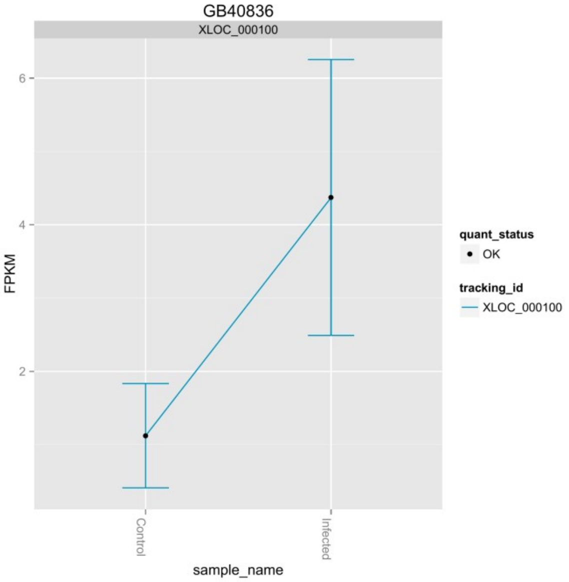
GB40836
XLOC_000100
6
4
2
FPKM
Control
Infected
sample_name
quant_status
OK
tracking_id
XLOC_000100

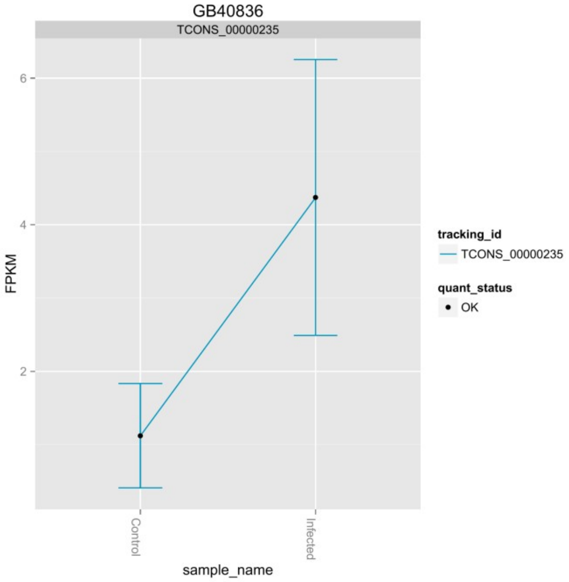
GB40836
TCONS_00000235
6
4
2
FPKM
Control
Infected
sample_name
tracking_id
TCONS_00000235
quant_status
OK

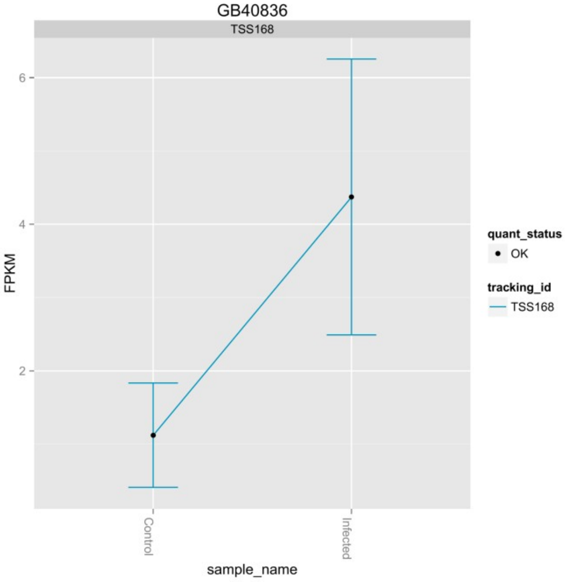
GB40836
TSS168
6
4
2
FPKM
Control
Infected
sample_name
quant_status
OK
tracking_id
TSS168

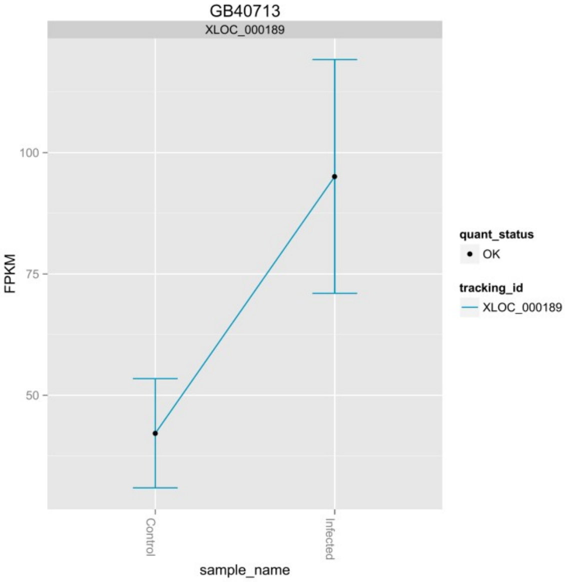
GB40713
XLOC_000189
100
75
50
FPKM
Control
Infected
sample_name
quant_status
OK
tracking_id
XLOC_000189

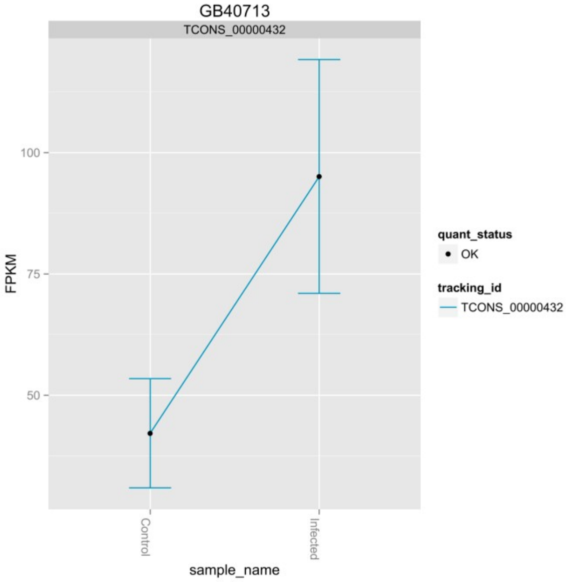
GB40713
TCONS_00000432
100
75
50
FPKM
Control
Infected
sample_name
quant_status
OK
tracking_id
TCONS_00000432

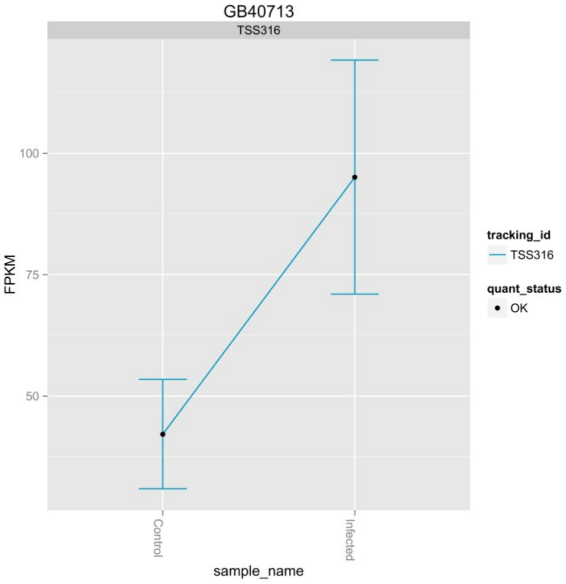
GB40713
TSS316
100
75
50
FPKM
Control
Infected
sample_name
tracking_id
TSS316
quant_status
OK

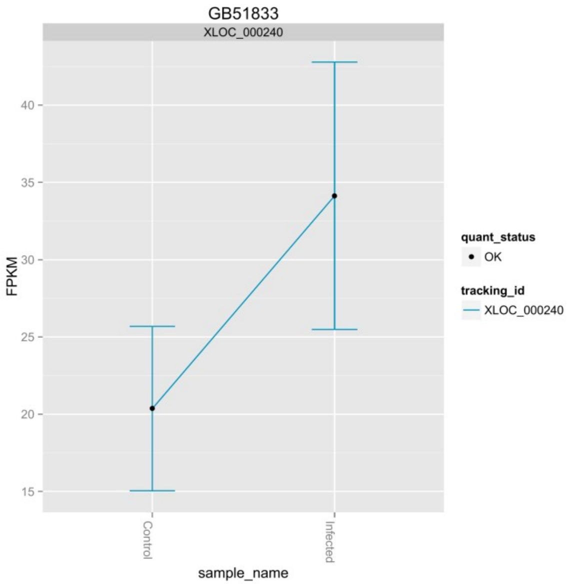
GB51833
XLOC_000240
FPKM
15
20
25
30
35
40
Control
Infected
sample_name
quant_status
OK
tracking_id
XLOC_000240

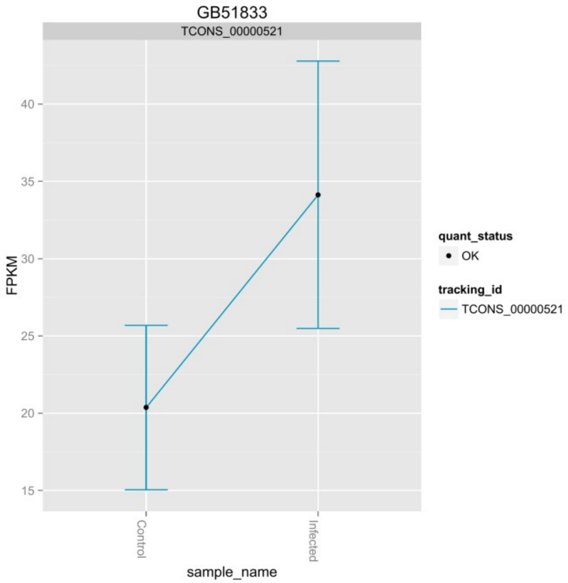
GB51833
TCONS_00000521
FPKM
15
20
25
30
35
40
Control
Infected
sample_name
quant_status
OK
tracking_id
TCONS_00000521

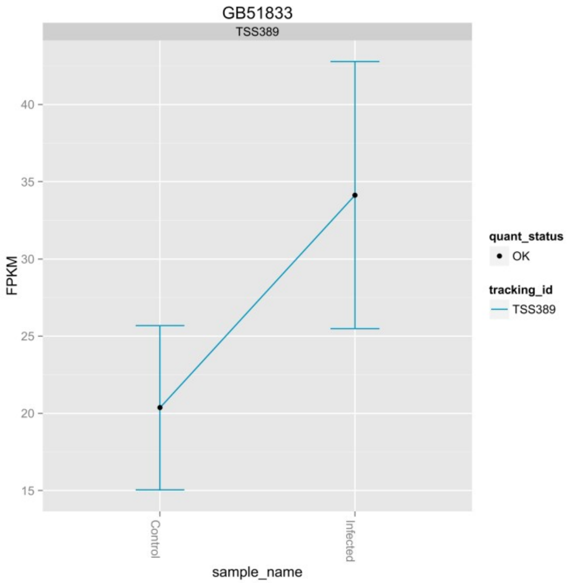
GB51833
TSS389
FPKM
15
20
25
30
35
40
Control
Infected
sample_name
quant_status
OK
tracking_id
TSS389

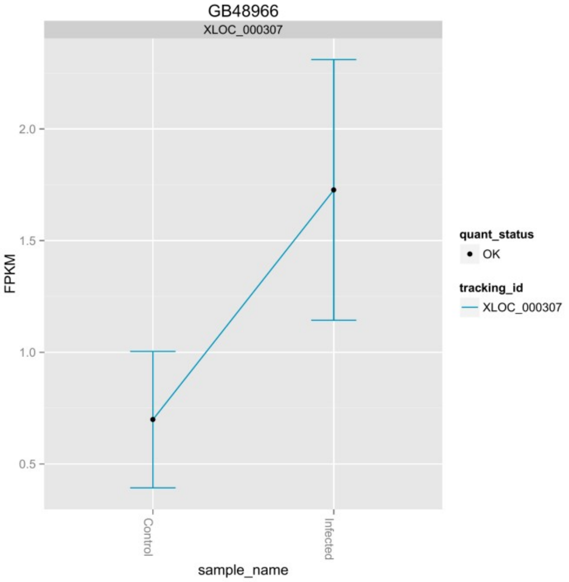

GB48966
XLOC_000307
FPKM
2.0
1.5
1.0
0.5
Control
Infected
sample_name
quant_status
OK
tracking_id
XLOC_000307

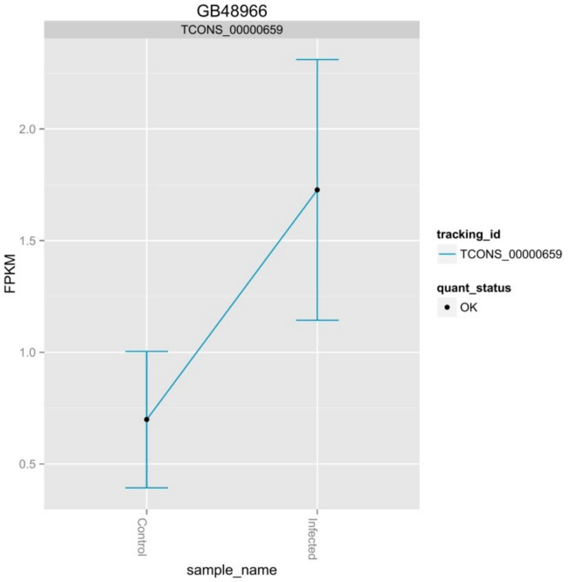

GB48966
TCONS_00000659
FPKM
2.0
1.5
1.0
0.5
Control
Infected
sample_name
tracking_id
TCONS_00000659
quant_status
OK

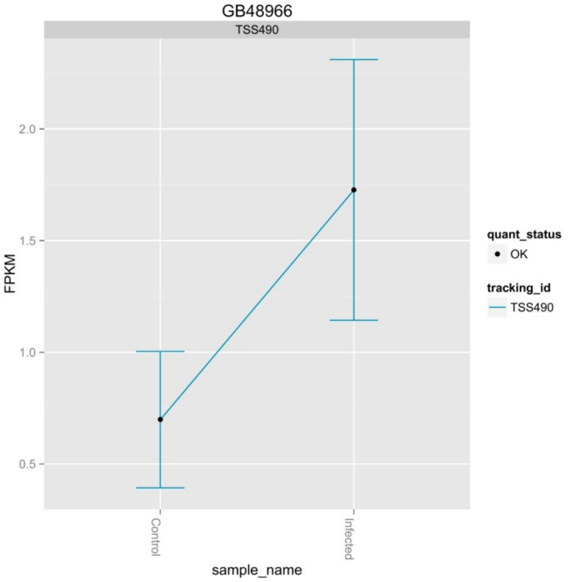

GB48966
TSS490
FPKM
2.0
1.5
1.0
0.5
Control
Infected
sample_name
quant_status
OK
tracking_id
TSS490

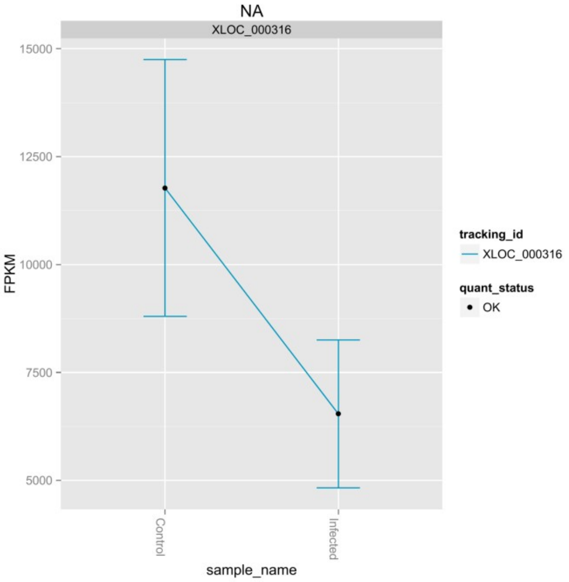
NA
XLOC_000316
15000
12500
10000
7500
5000
FPKM
Control
Infected
sample_name
tracking_id
XLOC_000316
quant_status
OK

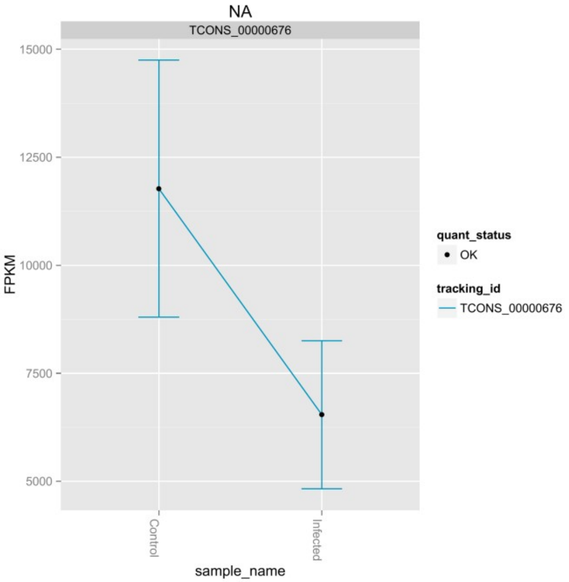
NA
TCONS_00000676
15000
12500
10000
7500
5000
FPKM
Control
Infected
sample_name
quant_status
OK
tracking_id
TCONS_00000676

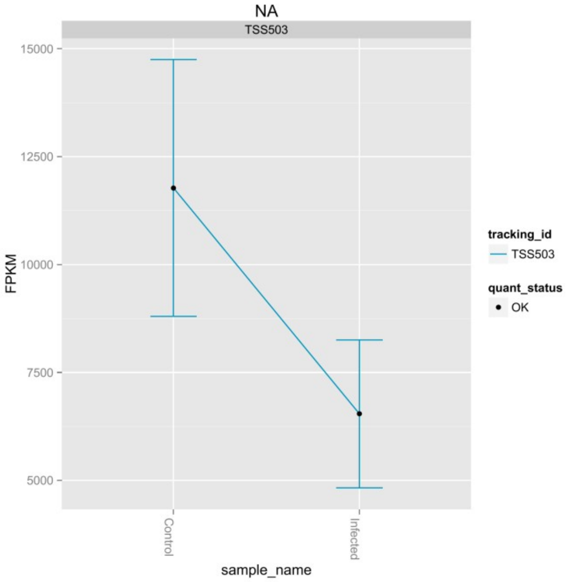
NA
TSS503
15000
12500
10000
7500
5000
FPKM
Control
Infected
sample_name
tracking_id
TSS503
quant_status
OK

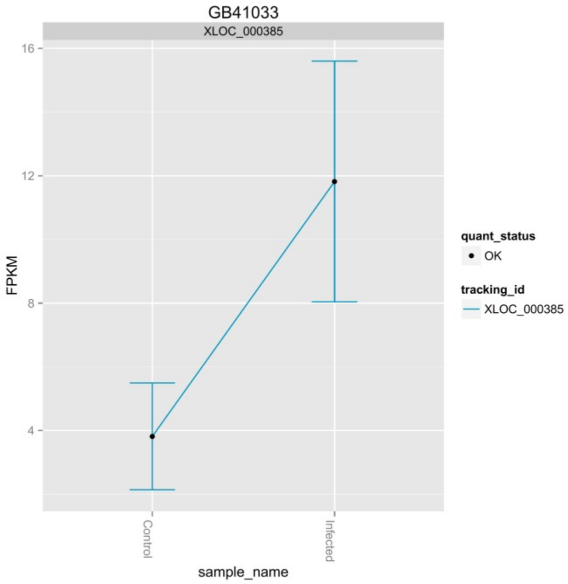
GB41033
XLOC_000385
16
12
8
4
FPKM
Control
Infected
sample_name
quant_status
OK
tracking_id
XLOC_000385

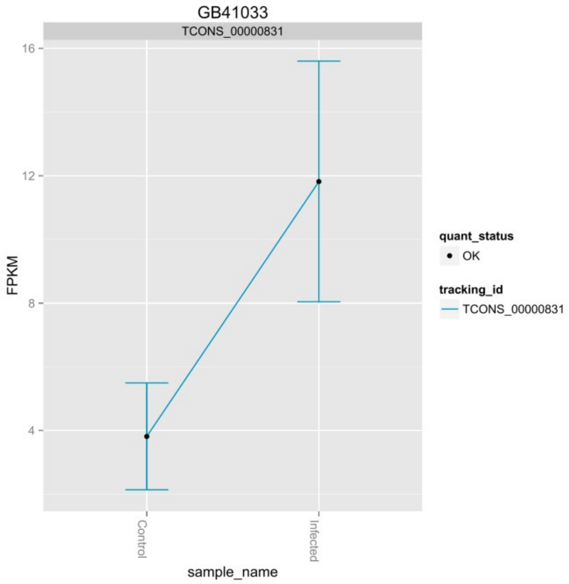
GB41033
TCONS_00000831
16
12
8
4
FPKM
Control
Infected
sample_name
quant_status
OK
tracking_id
TCONS_00000831

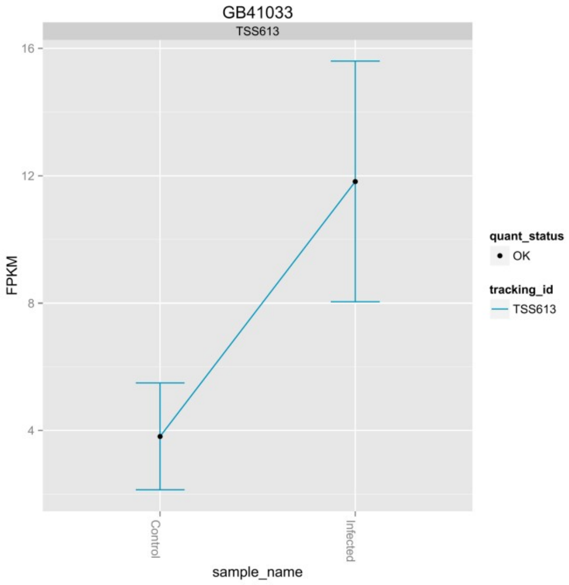
GB41033
TSS613
16
12
8
4
FPKM
Control
Infected
sample_name
quant_status
OK
tracking_id
TSS613

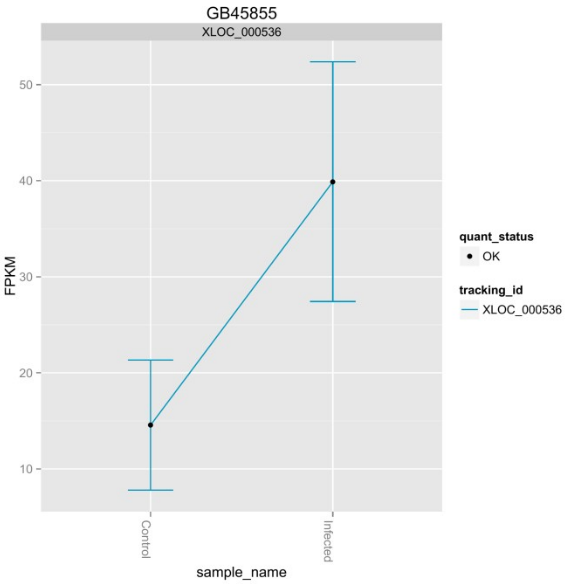

GB45855
XLOC_000536
FPKM
sample_name
Control
Infected
quant_status
OK
tracking_id
XLOC_000536

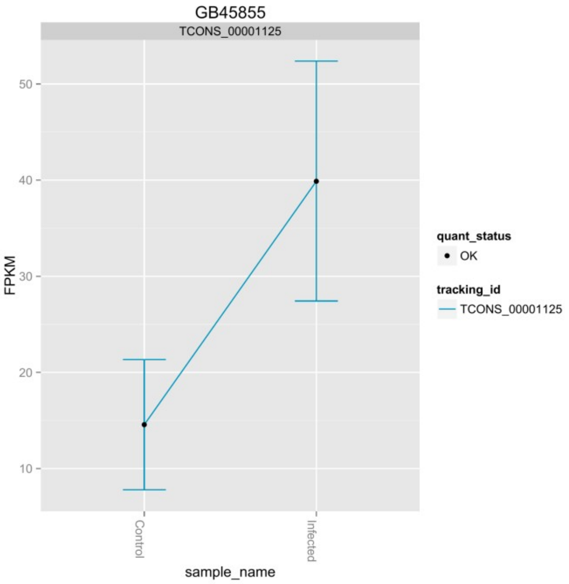

GB45855
TCONS_00001125
FPKM
sample_name
Control
Infected
quant_status
OK
tracking_id
TCONS_00001125

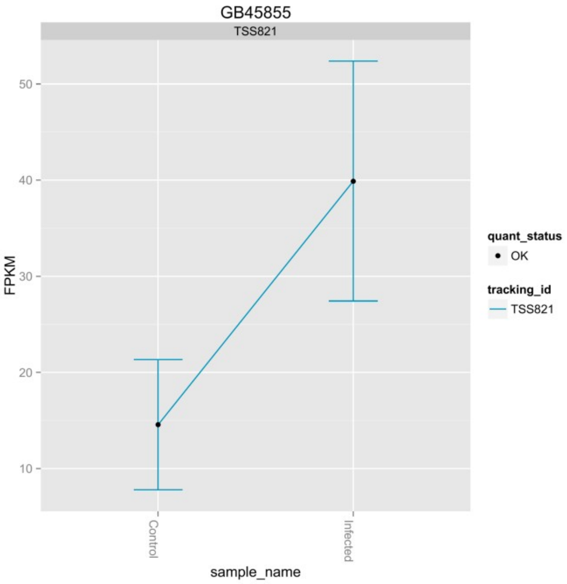

GB45855
TSS821
FPKM
sample_name
Control
Infected
quant_status
OK
tracking_id
TSS821

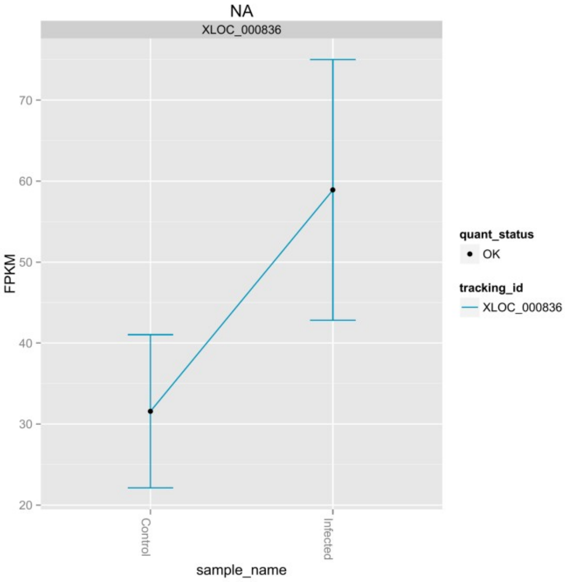
NA
XLOC_000836
FPKM
70
60
50
40
30
20
Control
Infected
sample_name
quant_status
OK
tracking_id
XLOC_000836

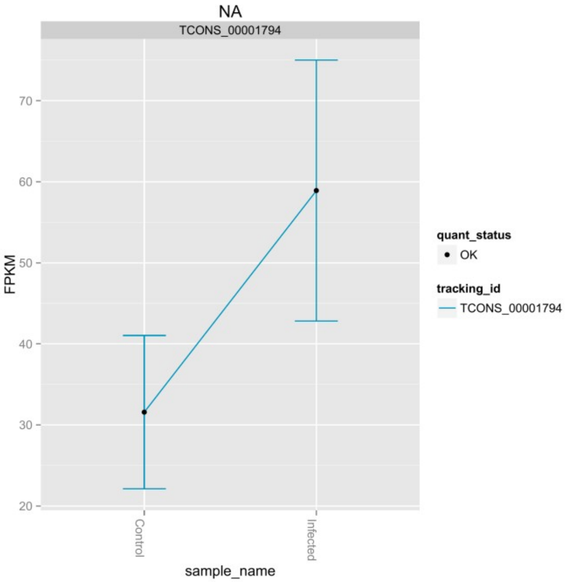
NA
TCONS_00001794
FPKM
70
60
50
40
30
20
Control
Infected
sample_name
quant_status
OK
tracking_id
TCONS_00001794

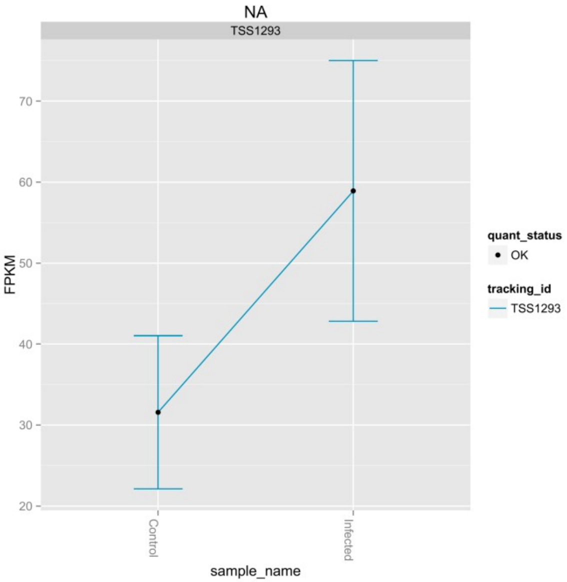
NA
TSS1293
FPKM
70
60
50
40
30
20
Control
Infected
sample_name
quant_status
OK
tracking_id
TSS1293

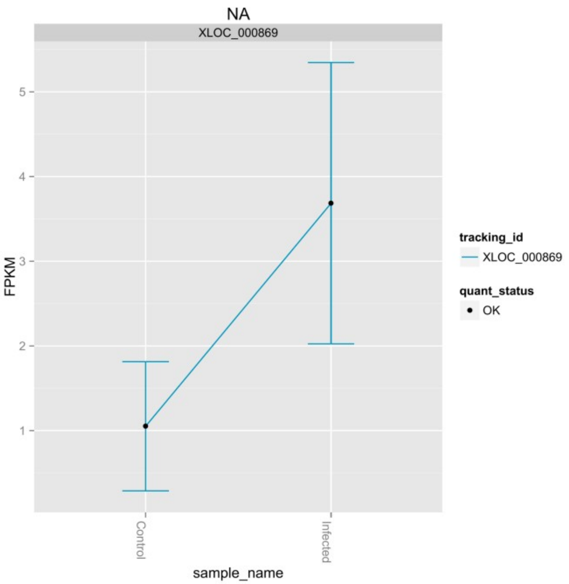
NA
XLOC_000869
FPKM
5
4
3
2
1
Control
Infected
sample_name
tracking_id
XLOC_000869
quant_status
OK

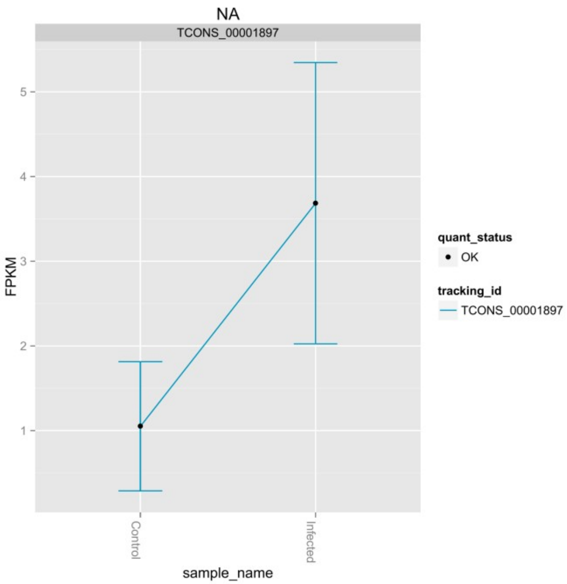
NA
TCONS_00001897
FPKM
5
4
3
2
1
Control
Infected
sample_name
quant_status
OK
tracking_id
TCONS_00001897

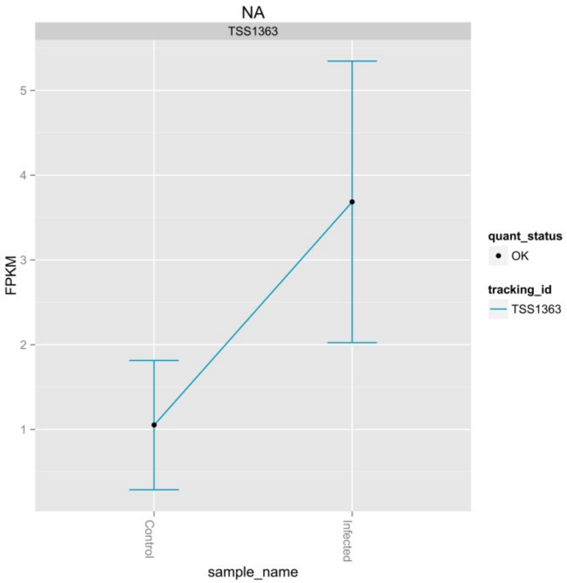
NA
TSS1363
FPKM
5
4
3
2
1
Control
Infected
sample_name
quant_status
OK
tracking_id
TSS1363

Group 2: spliced and transcriptionally regulated genes

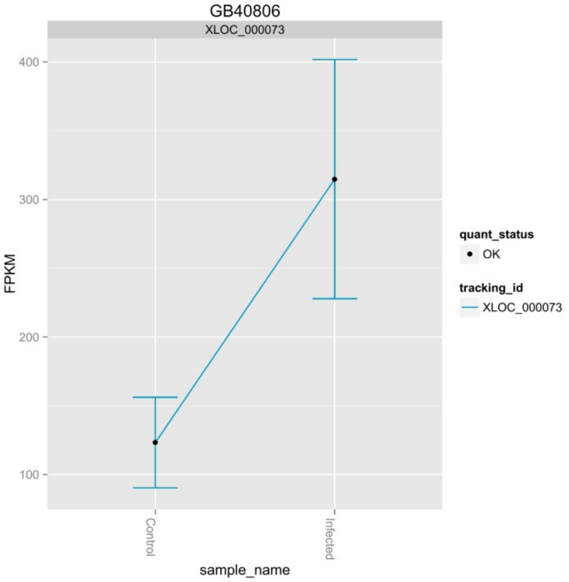
GB40806
XLOC_000073
400
300
200
100
FPKM
Control
Infected
sample_name
quant_status
OK
tracking_id
XLOC_000073

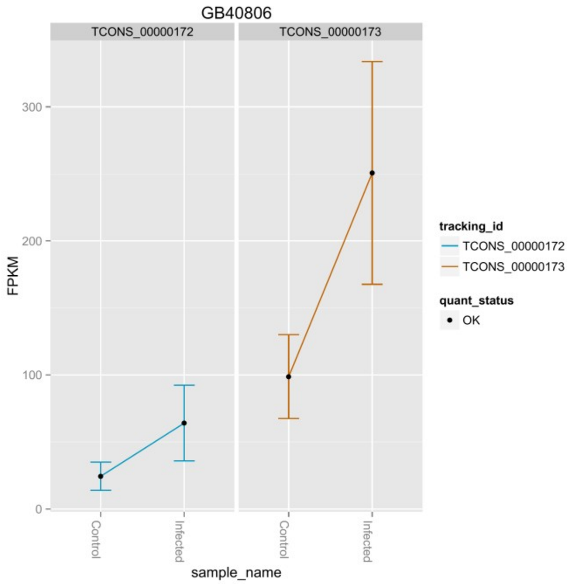
GB40806
TCONS_00000172
TCONS_00000173
300
200
100
0
FPKM
Control
Infected
Control
Infected
sample_name
tracking_id
TCONS_00000172
TCONS_00000173
quant_status
OK

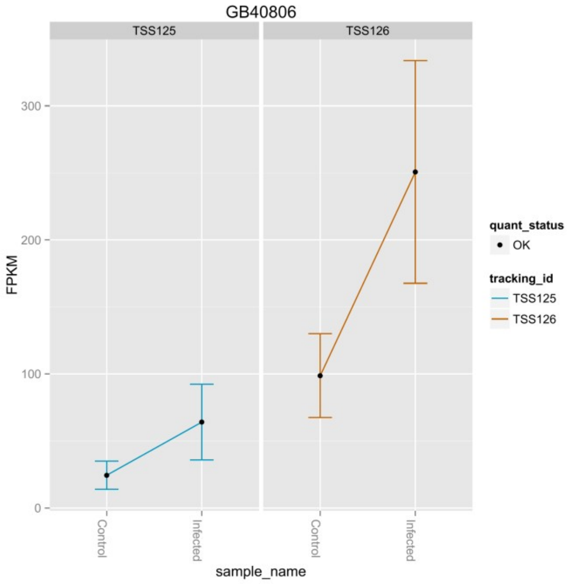
GB40806
TSS125
TSS126
300
200
100
0
FPKM
Control
Infected
Control
Infected
sample_name
quant_status
OK
tracking_id
TSS125
TSS126

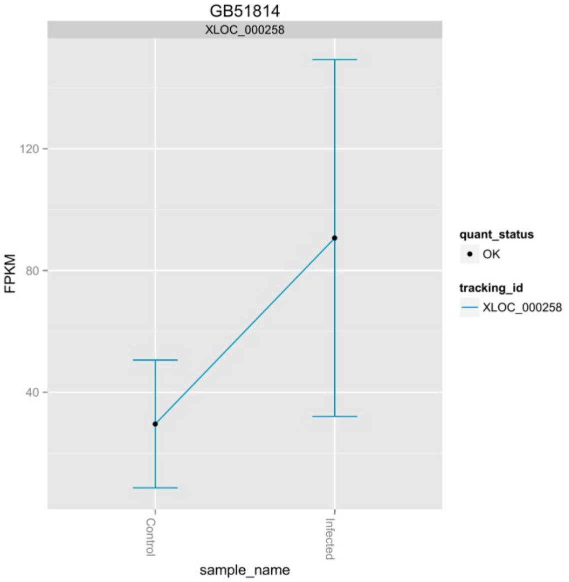
GB51814
XLOC_000258
FPKM
120
80
40
Control
Infected
sample_name
quant_status
OK
tracking_id
XLOC_000258

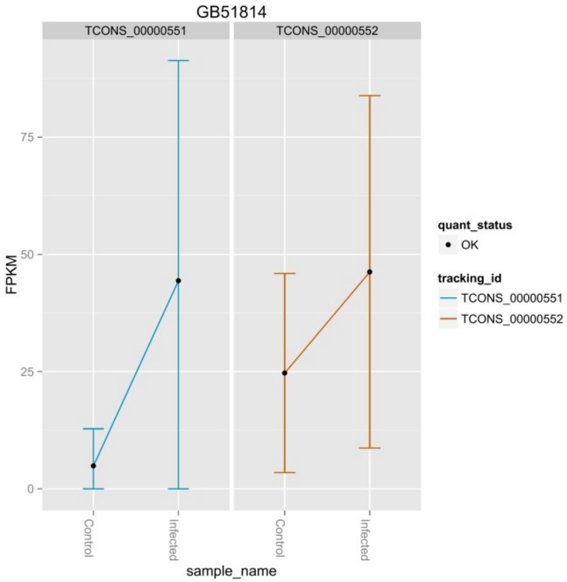
GB51814
TCONS_00000551
TCONS_00000552
FPKM
75
50
25
0
Control
Infected
Control
Infected
sample_name
quant_status
OK
tracking_id
TCONS_00000551
TCONS_00000552

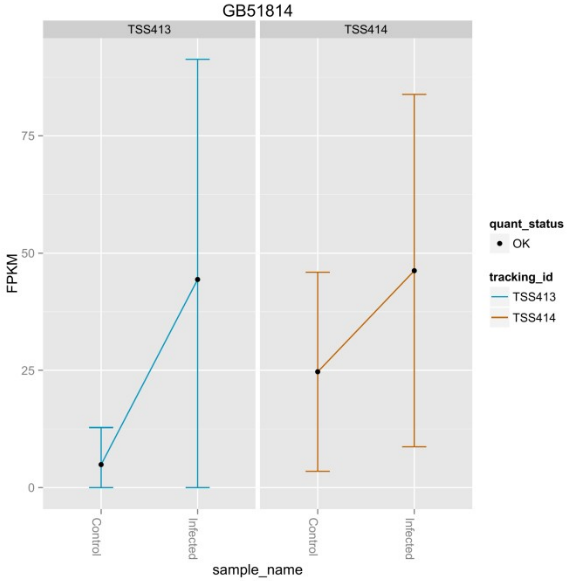
GB51814
TSS413
TSS414
FPKM
75
50
25
0
Control
Infected
Control
Infected
sample_name
quant_status
OK
tracking_id
TSS413
TSS414

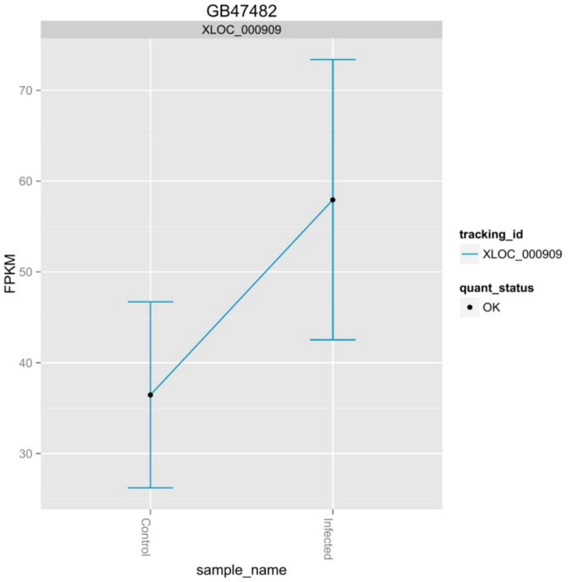
GB47482
XLOC_000909
FPKM
70
60
50
40
30
Control
Infected
sample_name
tracking_id
XLOC_000909
quant_status
OK

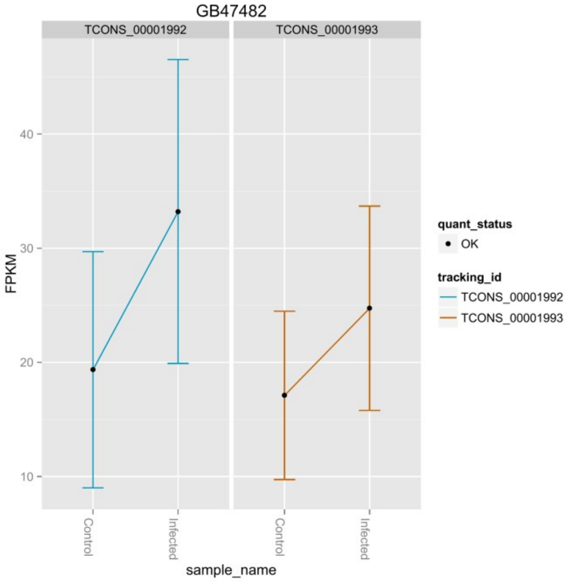
GB47482
TCONS_00001992
TCONS_00001993
FPKM
40
30
20
10
Control
Infected
Control
Infected
sample_name
quant_status
OK
tracking_id
TCONS_00001992
TCONS_00001993

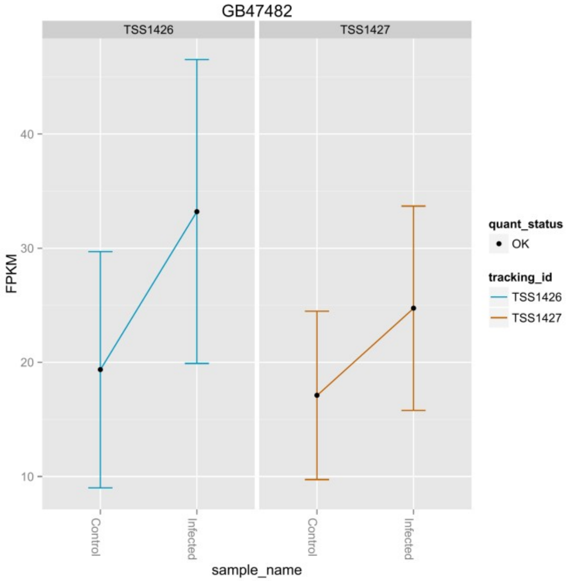
GB47482
TSS1426
TSS1427
FPKM
40
30
20
10
Control
Infected
Control
Infected
sample_name
quant_status
OK
tracking_id
TSS1426
TSS1427

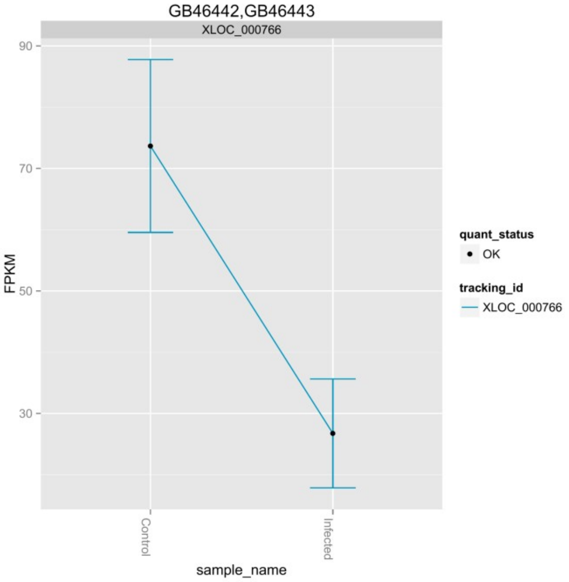
GB46442,GB46443
XLOC_000766
90
70
50
30
FPKM
Control
Infected
sample_name
quant_status
OK
tracking_id
XLOC_000766

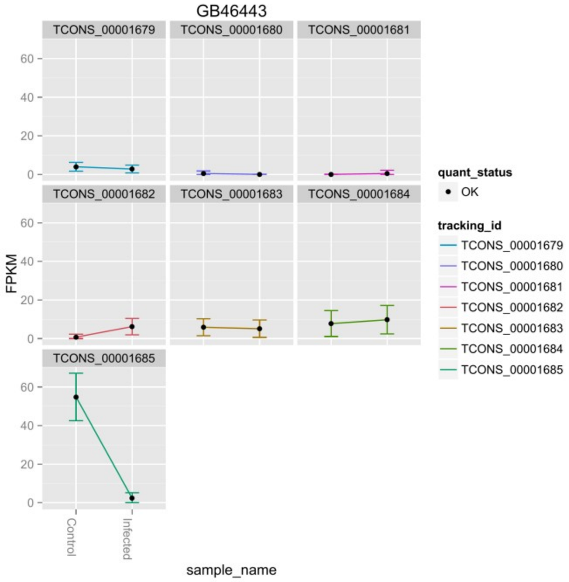
GB46443
TCONS_00001679
TCONS_00001680
TCONS_00001681
TCONS_00001682
TCONS_00001683
TCONS_00001684
TCONS_00001685
60
40
20
0
FPKM
Control
Infected
sample_name
quant_status
OK
tracking_id
TCONS_00001679
TCONS_00001680
TCONS_00001681
TCONS_00001682
TCONS_00001683
TCONS_00001684
TCONS_00001685

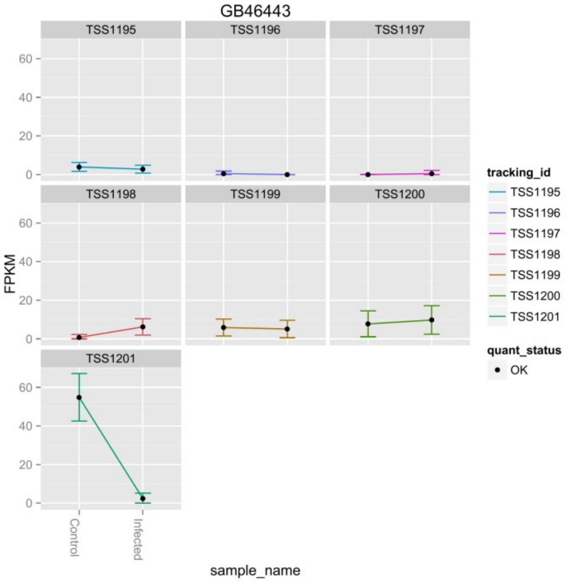
GB46443
TSS1195
TSS1196
TSS1197
TSS1198
TSS1199
TSS1200
TSS1201
60
40
20
0
FPKM
Control
Infected
sample_name
tracking_id
TSS1195
TSS1196
TSS1197
TSS1198
TSS1199
TSS1200
TSS1201
quant_status
OK

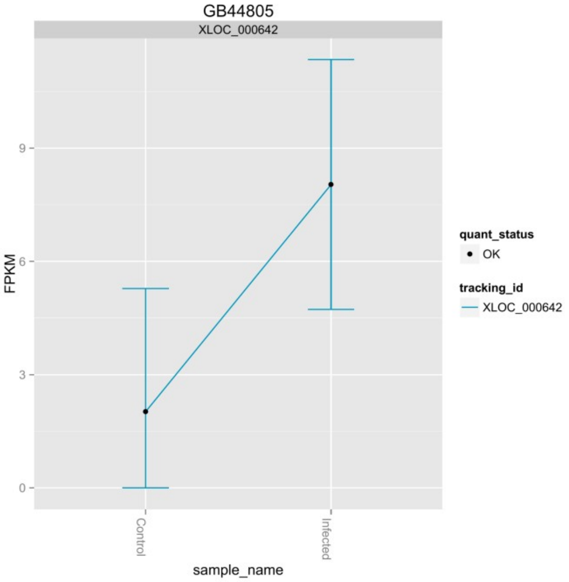
GB44805
XLOC_000642
FPKM
sample_name
Control
Infected
quant_status
OK
tracking_id
XLOC_000642

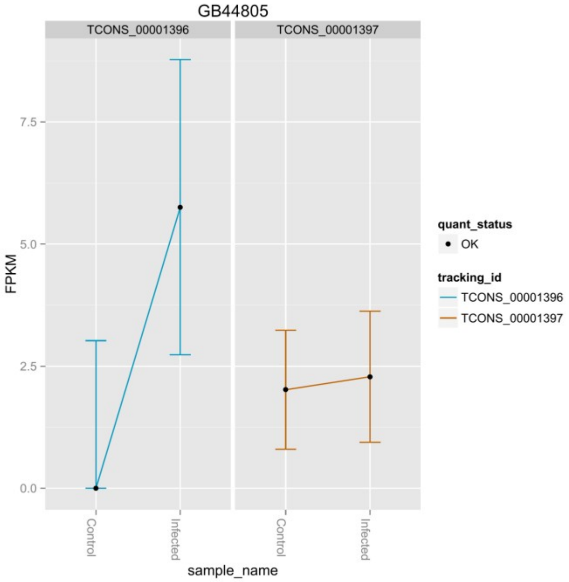
GB44805
TCONS_00001396
TCONS_00001397
FPKM
sample_name
Control
Infected
quant_status
OK
tracking_id
TCONS_00001396
TCONS_00001397

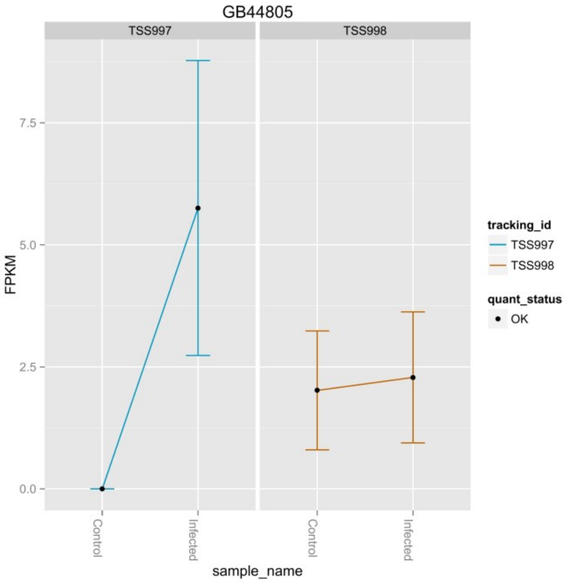
GB44805
TSS997
TSS998
FPKM
sample_name
Control
Infected
tracking_id
TSS997
TSS998
quant_status
OK

Group 3:
Spliced and both transcriptionally and post-transcriptionally regulated

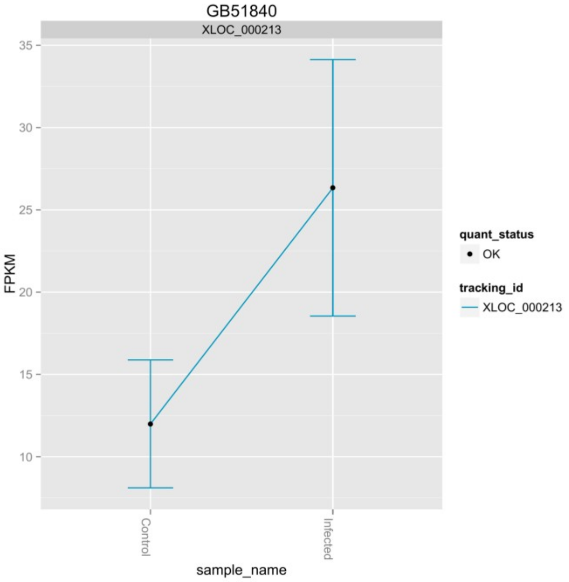
GB51840
XLOC_000213
FPKM
35
30
25
20
15
10
Control
Infected
sample_name
quant_status
OK
tracking_id
XLOC_000213

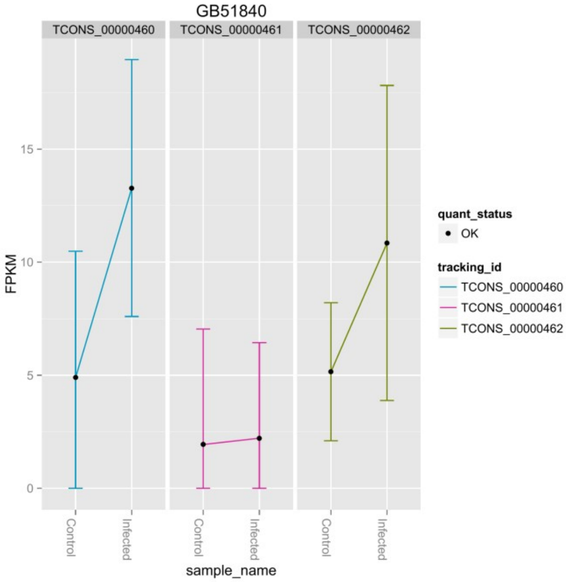
GB51840
TCONS_00000460
TCONS_00000461
TCONS_00000462
FPKM
15
10
5
0
Control
Infected
Control
Infected
Control
Infected
sample_name
quant_status
OK
tracking_id
TCONS_00000460
TCONS_00000461
TCONS_00000462

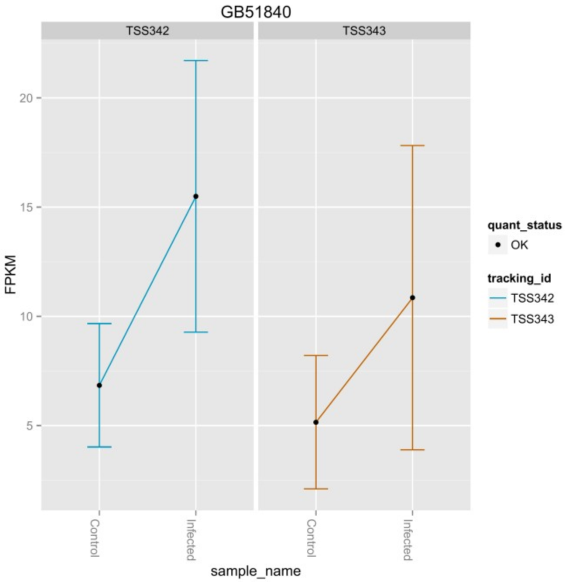
GB51840
TSS342
TSS343
FPKM
20
15
10
5
Control
Infected
Control
Infected
sample_name
quant_status
OK
tracking_id
TSS342
TSS343

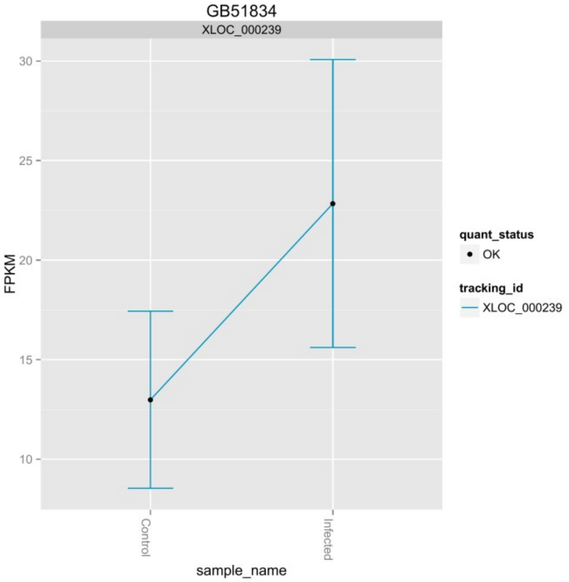
GB51834
XLOC_000239
FPKM
sample_name
Control
Infected
quant_status
OK
tracking_id
XLOC_000239

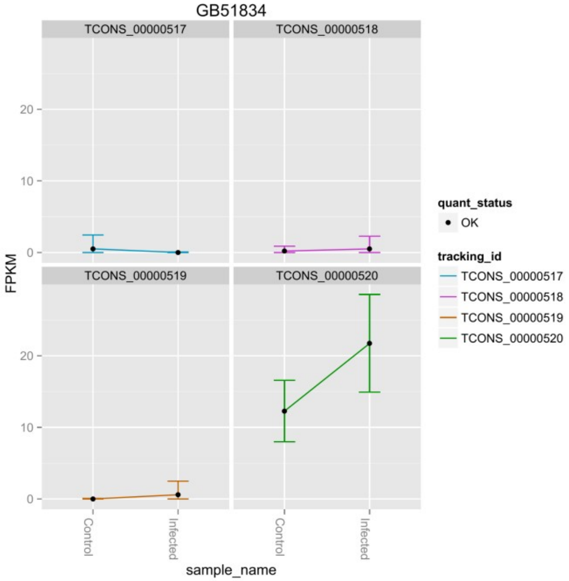
GB51834
TCONS_00000517
TCONS_00000518
TCONS_00000519
TCONS_00000520
FPKM
sample_name
Control
Infected
quant_status
OK
tracking_id
TCONS_00000517
TCONS_00000518
TCONS_00000519
TCONS_00000520

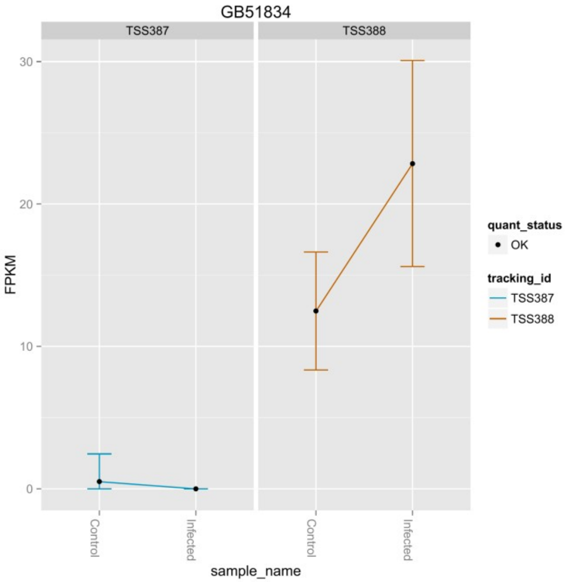
GB51834
TSS387
TSS388
FPKM
sample_name
Control
Infected
quant_status
OK
tracking_id
TSS387
TSS388

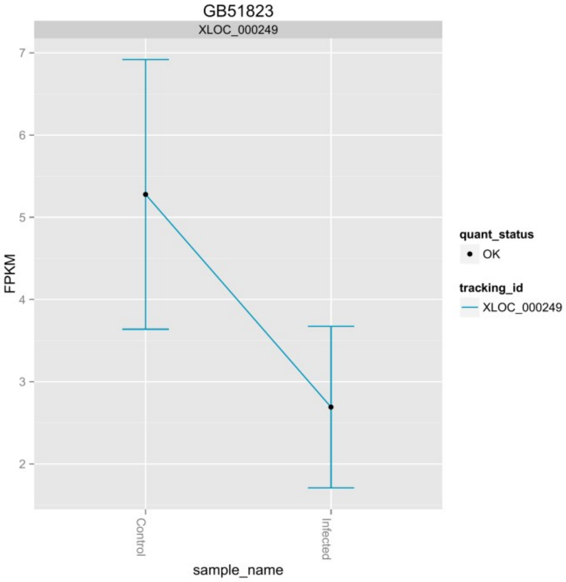
GB51823
XLOC_000249
FPKM
7
6
5
4
3
2
Control
Infected
sample_name
quant_status
OK
tracking_id
XLOC_000249

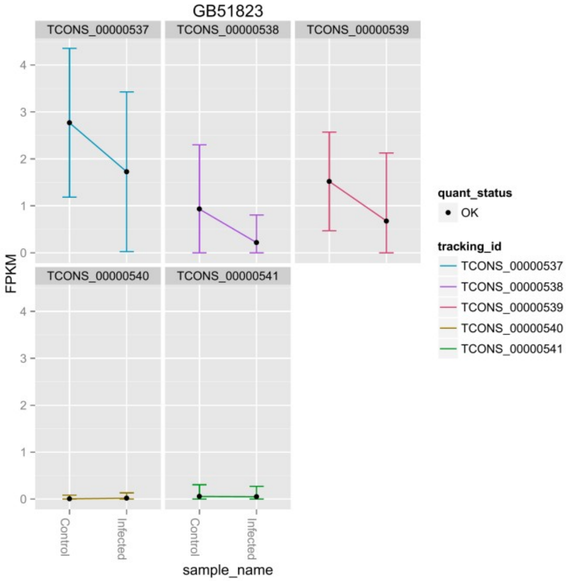
GB51823
TCONS_00000537
TCONS_00000538
TCONS_00000539
TCONS_00000540
TCONS_00000541
FPKM
4
3
2
1
0
Control
Infected
sample_name
quant_status
OK
tracking_id
TCONS_00000537
TCONS_00000538
TCONS_00000539
TCONS_00000540
TCONS_00000541

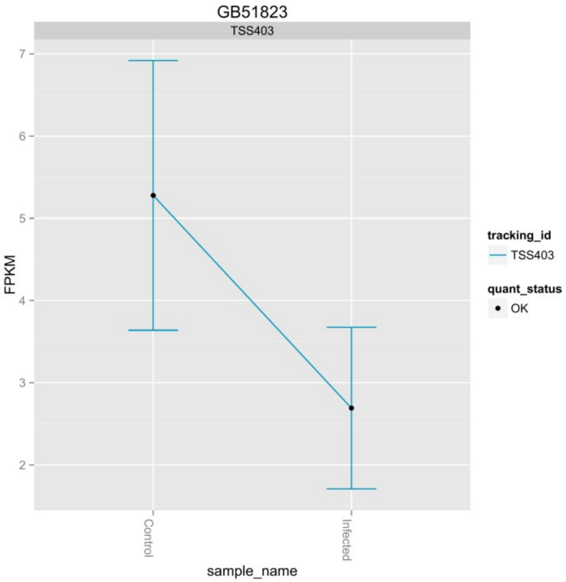
GB51823
TSS403
FPKM
7
6
5
4
3
2
Control
Infected
sample_name
tracking_id
TSS403
quant_status
OK

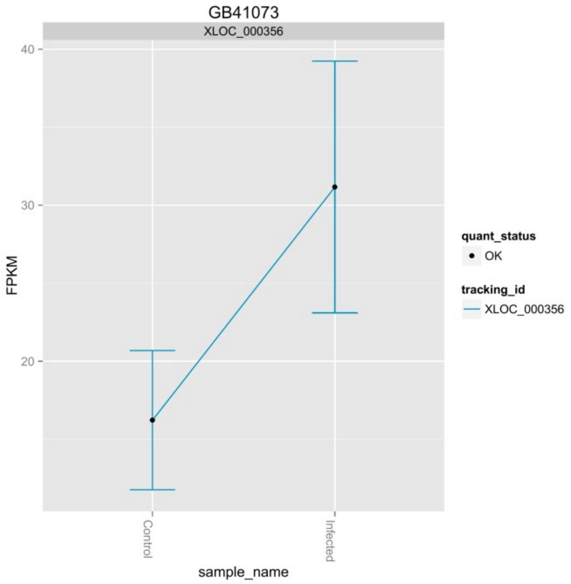
GB41073
XLOC_000356
40
30
20
FPKM
Control
Infected
sample_name
quant_status
OK
tracking_id
XLOC_000356

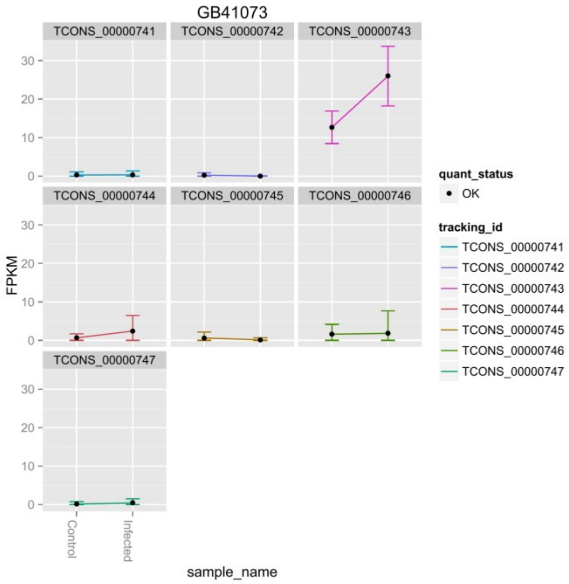
GB41073
TCONS_00000741
TCONS_00000742
TCONS_00000743
TCONS_00000744
TCONS_00000745
TCONS_00000746
TCONS_00000747
30
20
10
0
FPKM
Control
Infected
sample_name
quant_status
OK
tracking_id
TCONS_00000741
TCONS_00000742
TCONS_00000743
TCONS_00000744
TCONS_00000745
TCONS_00000746
TCONS_00000747

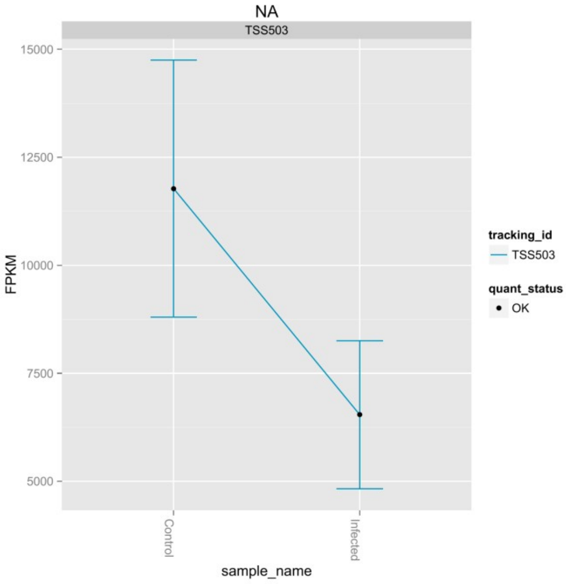
NA
TSS503
15000
12500
10000
7500
5000
FPKM
Control
Infected
sample_name
tracking_id
TSS503
quant_status
OK

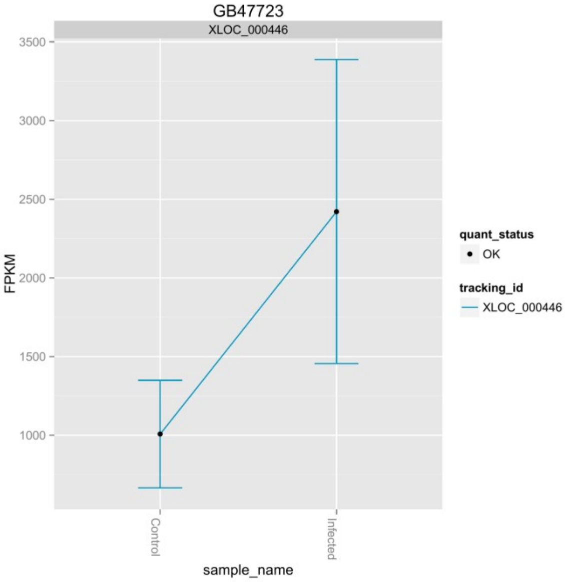
GB47723
XLOC_000446
FPKM
sample_name
Control
Infected
quant_status
OK
tracking_id
XLOC_000446

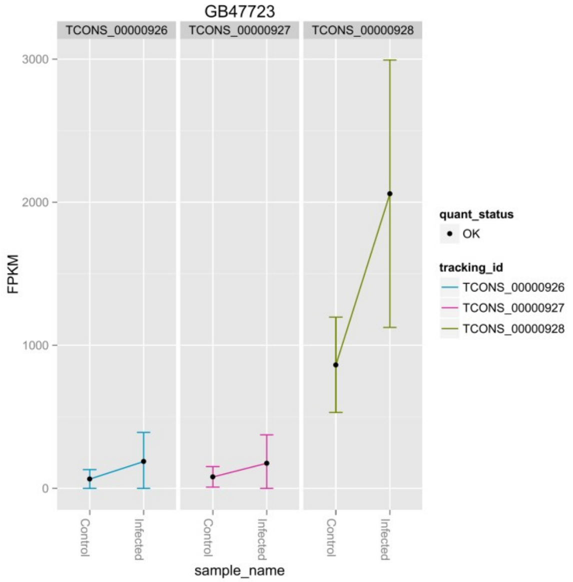
GB47723
TCONS_00000926
TCONS_00000927
TCONS_00000928
FPKM
sample_name
Control
Infected
quant_status
OK
tracking_id
TCONS_00000926
TCONS_00000927
TCONS_00000928

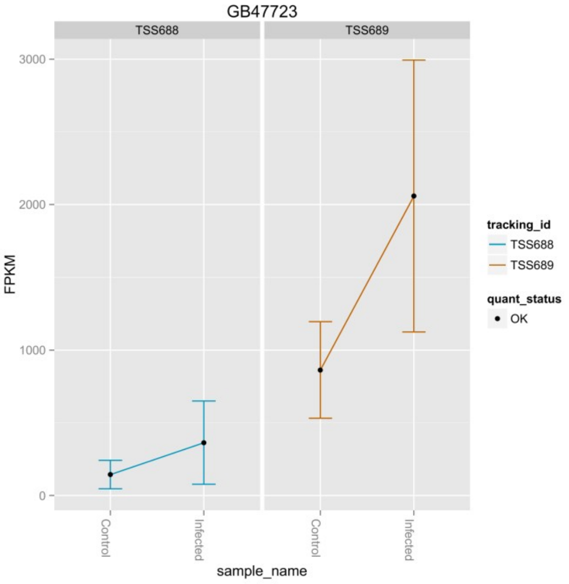
GB47723
TSS688
TSS689
FPKM
sample_name
Control
Infected
tracking_id
TSS688
TSS689
quant_status
OK

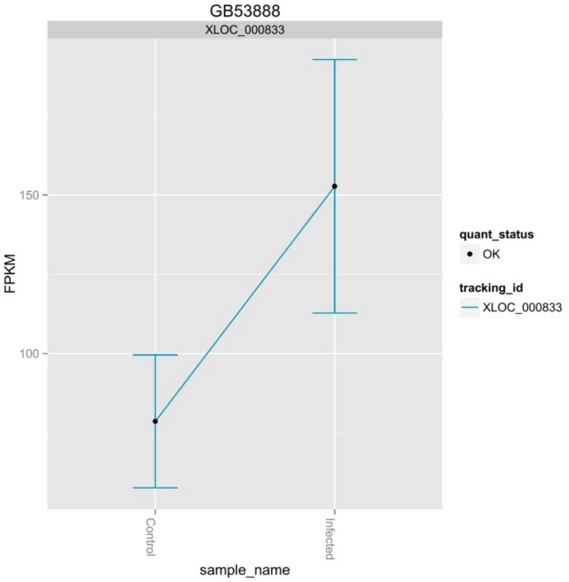
GB53888
XLOC_000833
150
100
FPKM
Control
Infected
sample_name
quant_status
OK
tracking_id
XLOC_000833

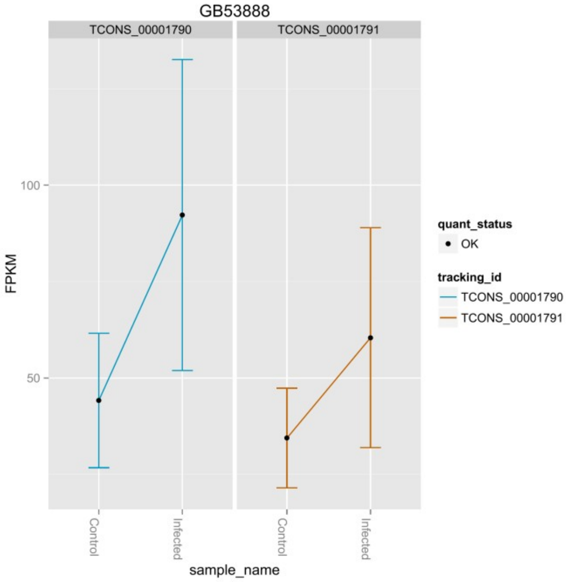
GB53888
TCONS_00001790
TCONS_00001791
100
50
FPKM
Control
Infected
Control
Infected
sample_name
quant_status
OK
tracking_id
TCONS_00001790
TCONS_00001791

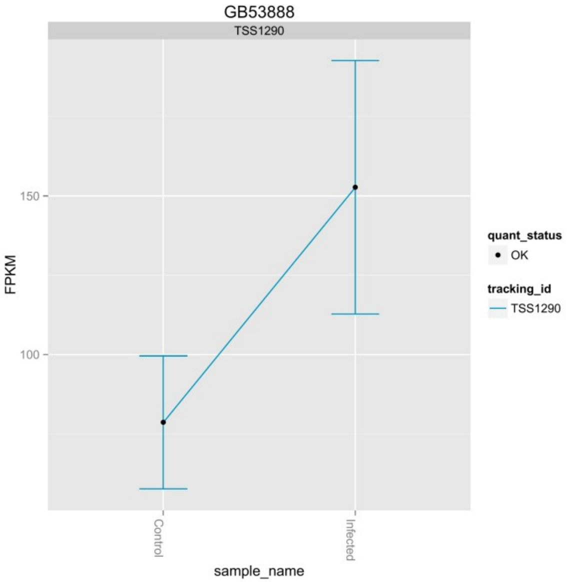
GB53888
TSS1290
150
100
FPKM
Control
Infected
sample_name
quant_status
OK
tracking_id
TSS1290
