# Supplementary material for: RNA-sequence analysis of gene expression from honeybees (Apis mellifera) infected with Nosema ceranae
Source: PLoS One. 2017 Mar 28;12(3):e0173438. doi: 10.1371/journal.pone.0173438 (PMC5370102; doi:10.1371/journal.pone.0173438)
Supplement: S6 Fig — (Group 1) Un-spliced and transcriptionally regulated genes,(Group 2) spliced and post-transcriptionally regulated genes and(Group 3) spliced and both transcriptionally and post-transcriptionally regulated genes. For each transcript,the “XLOC”, “TSS” and “TCONS” suffixes correspond to the genes, TSSs and isoforms, respectively. Differentially expressed isoforms with different TSSs are transcriptionally regulated, while isoforms with the same TSS are regulated at the post-transcriptional level. (PDF) [file pone.0173438.s011.pdf]

Group 1: un-spliced and transcriptionally regulated genes

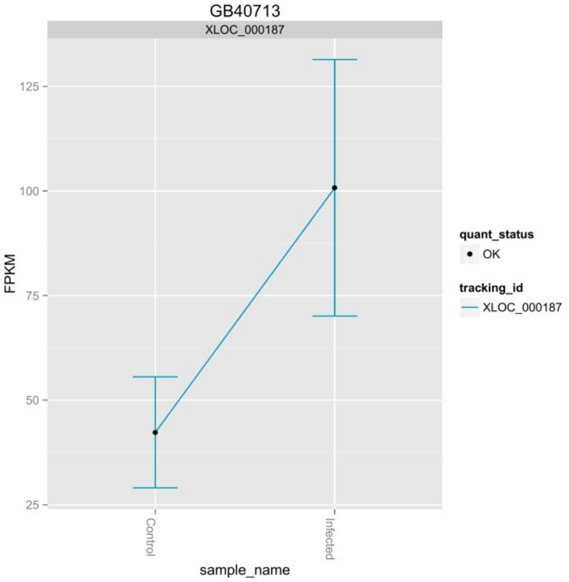

GB40713
XLOC_000187
FPKM
125
100
75
50
25
Control
Infected
sample_name
quant_status
OK
tracking_id
XLOC_000187

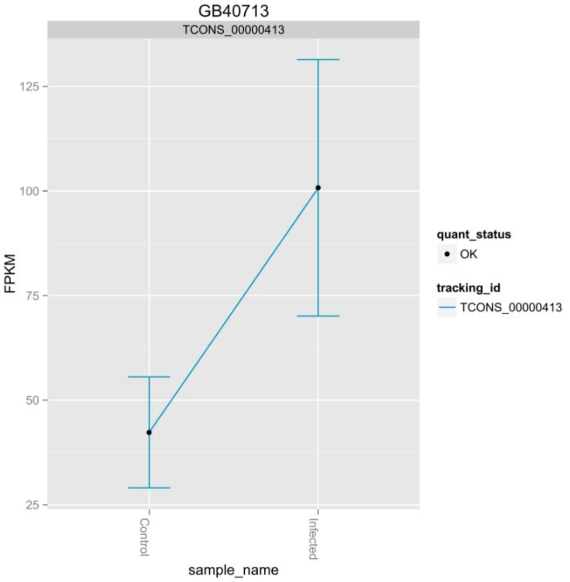

GB40713
TCONS_00000413
FPKM
125
100
75
50
25
Control
Infected
sample_name
quant_status
OK
tracking_id
TCONS_00000413

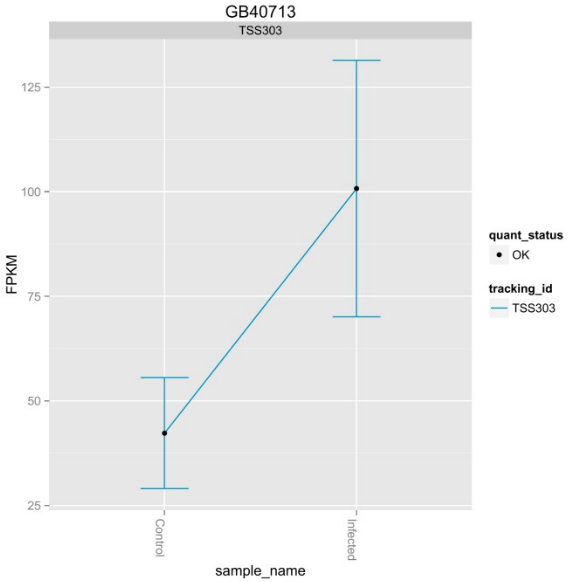

GB40713
TSS303
FPKM
125
100
75
50
25
Control
Infected
sample_name
quant_status
OK
tracking_id
TSS303

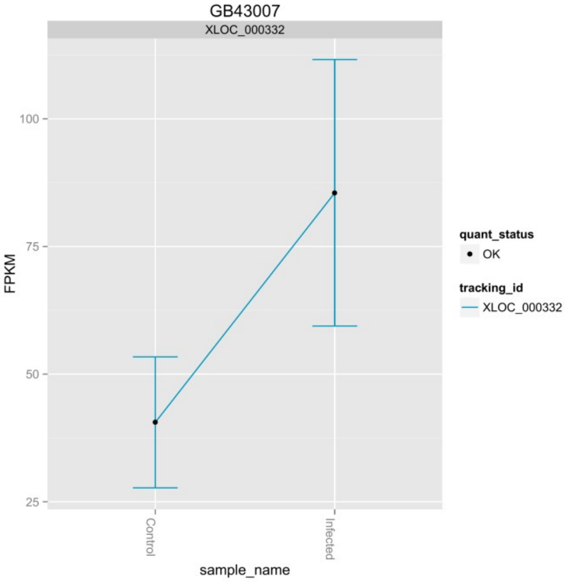
GB43007
XLOC_000332
100
75
50
25
FPKM
Control
Infected
sample_name
quant_status
OK
tracking_id
XLOC_000332

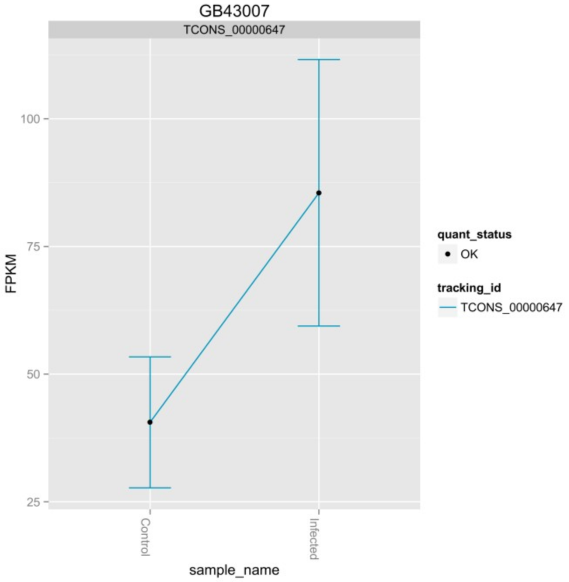
GB43007
TCONS_00000647
100
75
50
25
FPKM
Control
Infected
sample_name
quant_status
OK
tracking_id
TCONS_00000647

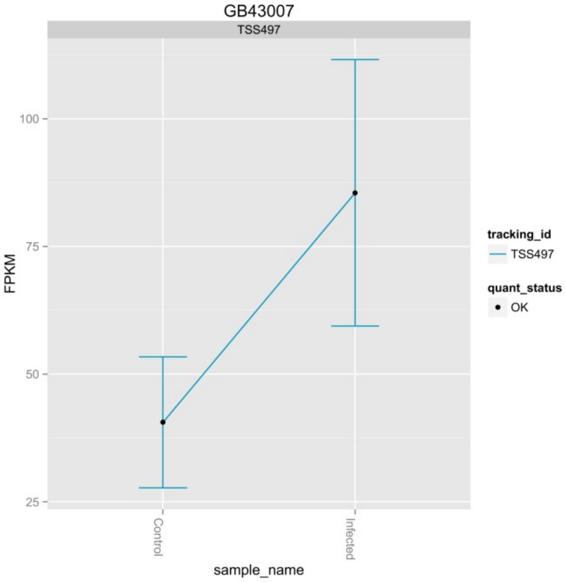
GB43007
TSS497
100
75
50
25
FPKM
Control
Infected
sample_name
tracking_id
TSS497
quant_status
OK

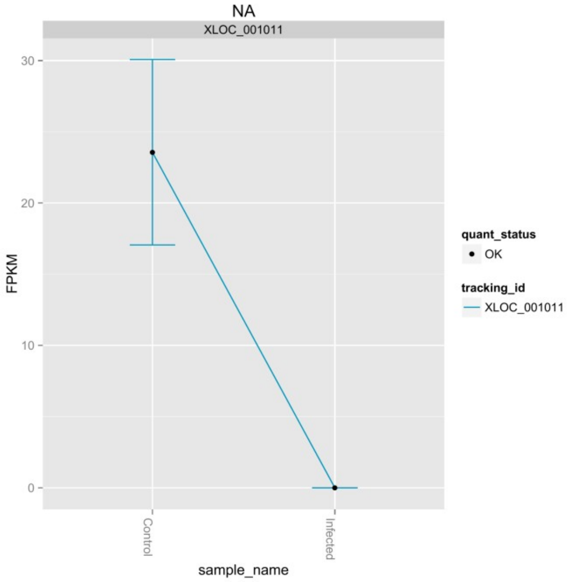
NA
XLOC_001011
30
20
10
0
FPKM
Control
Infected
sample_name
quant_status
OK
tracking_id
XLOC_001011

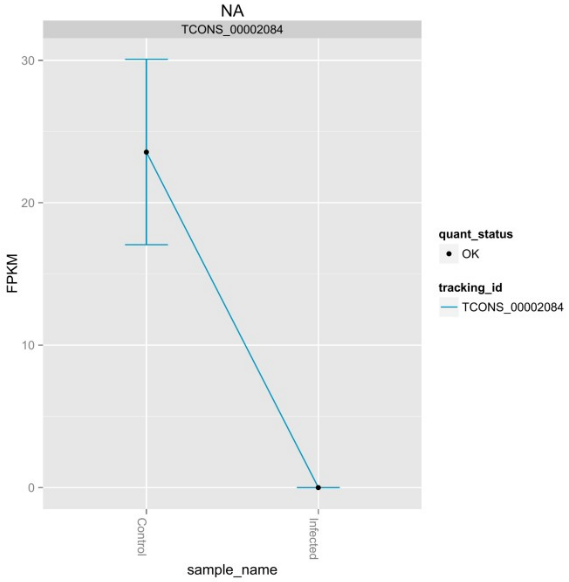
NA
TCONS_00002084
30
20
10
0
FPKM
Control
Infected
sample_name
quant_status
OK
tracking_id
TCONS_00002084

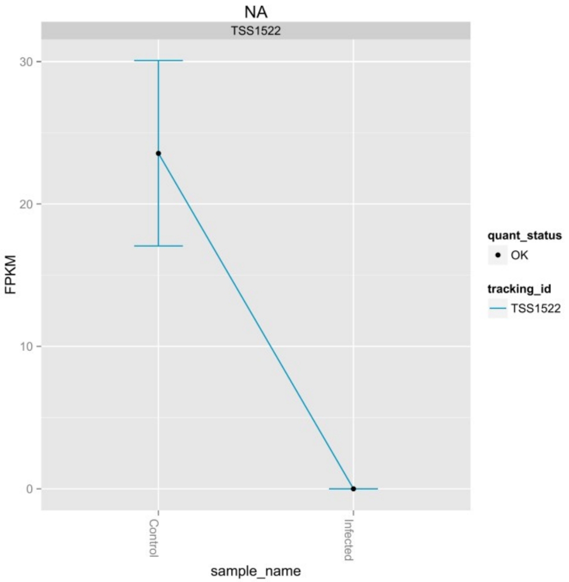
NA
TSS1522
30
20
10
0
FPKM
Control
Infected
sample_name
quant_status
OK
tracking_id
TSS1522

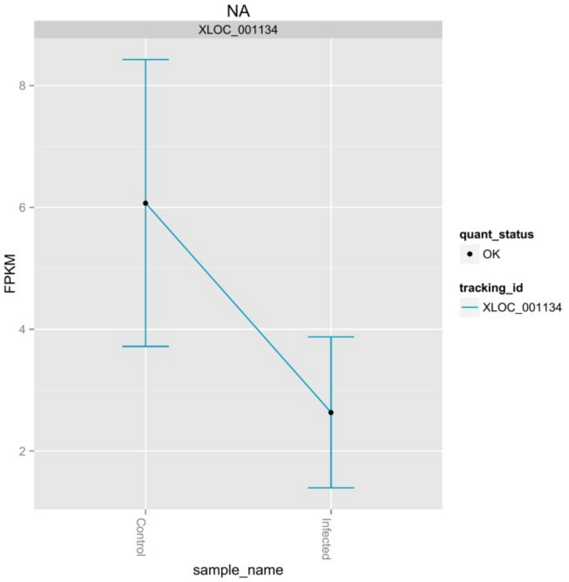
NA
XLOC_001134
8
6
4
2
FPKM
Control
Infected
sample_name
quant_status
OK
tracking_id
XLOC_001134

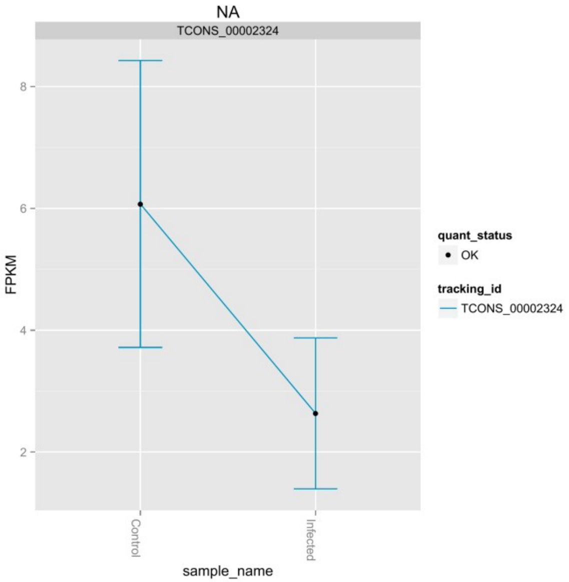
NA
TCONS_00002324
8
6
4
2
FPKM
Control
Infected
sample_name
quant_status
OK
tracking_id
TCONS_00002324

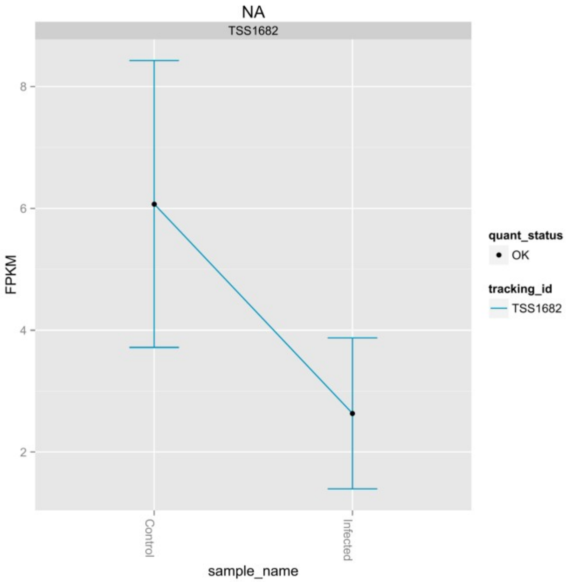
NA
TSS1682
8
6
4
2
FPKM
Control
Infected
sample_name
quant_status
OK
tracking_id
TSS1682

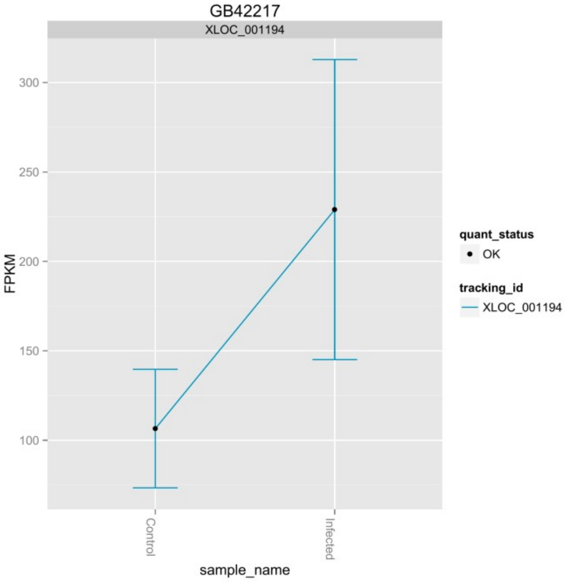
GB42217
XLOC_001194
FPKM
300
250
200
150
100
Control
Infected
sample_name
quant_status
OK
tracking_id
XLOC_001194

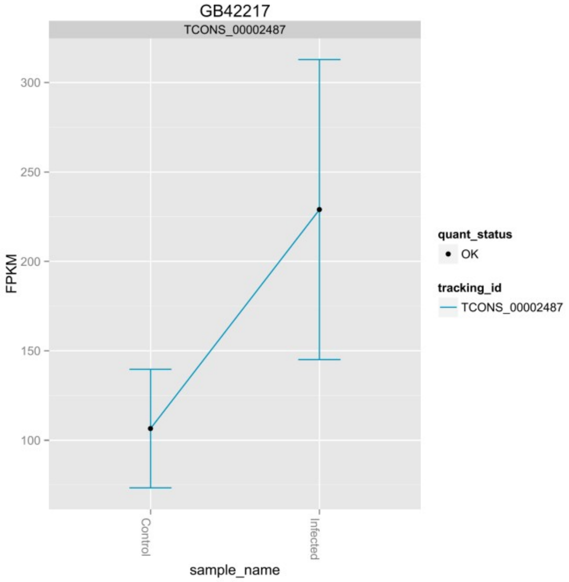
GB42217
TCONS_00002487
FPKM
300
250
200
150
100
Control
Infected
sample_name
quant_status
OK
tracking_id
TCONS_00002487

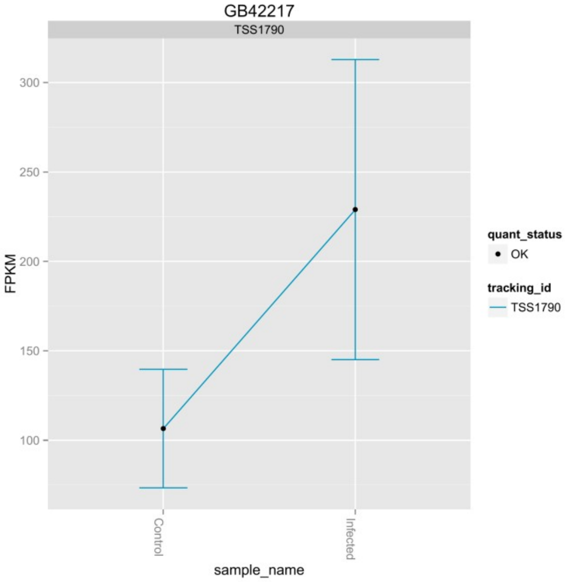
GB42217
TSS1790
FPKM
300
250
200
150
100
Control
Infected
sample_name
quant_status
OK
tracking_id
TSS1790

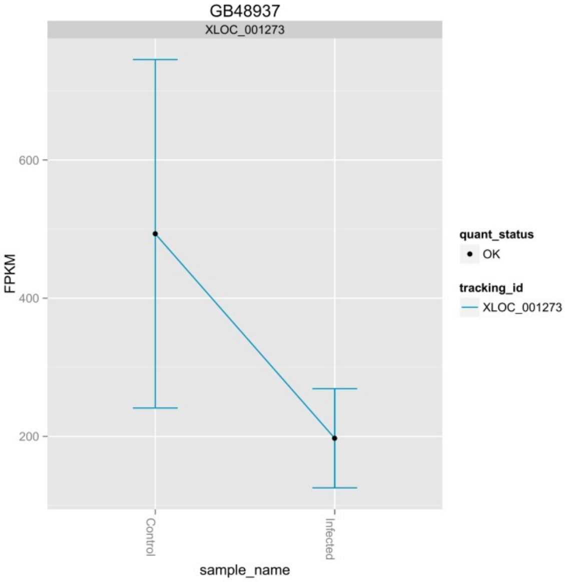

GB48937
XLOC_001273
FPKM
600
400
200
Control
Infected
sample_name
quant_status
OK
tracking_id
XLOC_001273

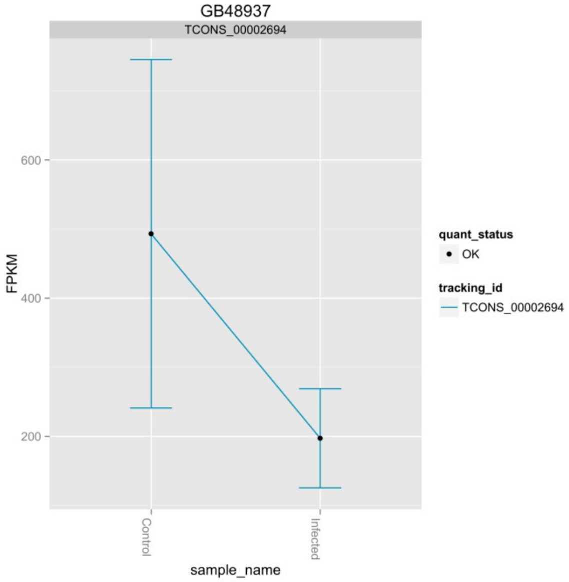

GB48937
TCONS_00002694
FPKM
600
400
200
Control
Infected
sample_name
quant_status
OK
tracking_id
TCONS_00002694

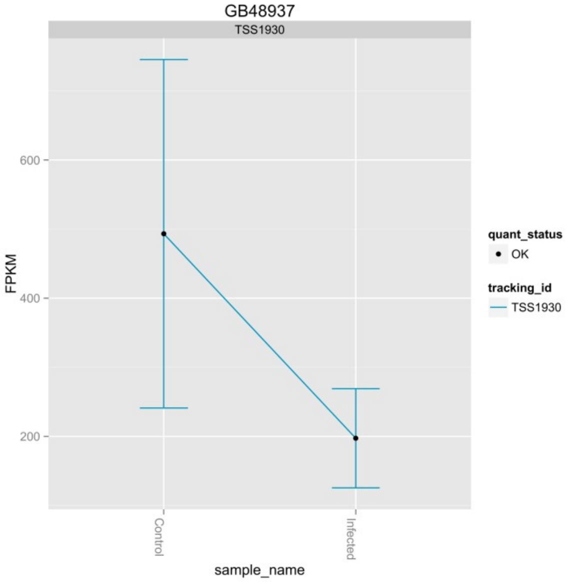

GB48937
TSS1930
FPKM
600
400
200
Control
Infected
sample_name
quant_status
OK
tracking_id
TSS1930

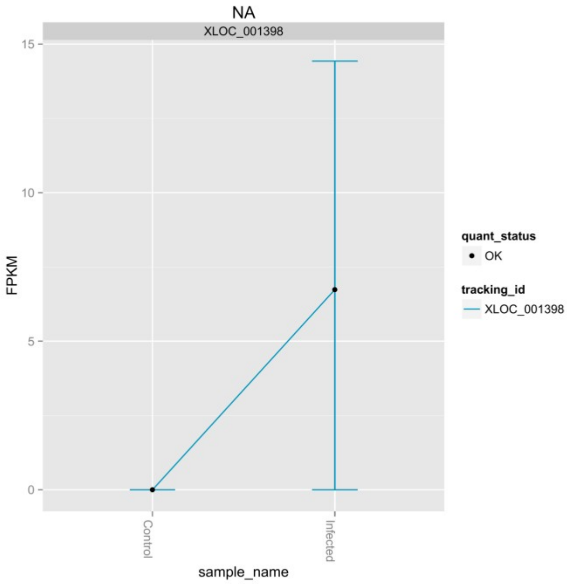
NA
XLOC_001398
15
10
5
0
FPKM
Control
Infected
sample_name
quant_status
OK
tracking_id
XLOC_001398

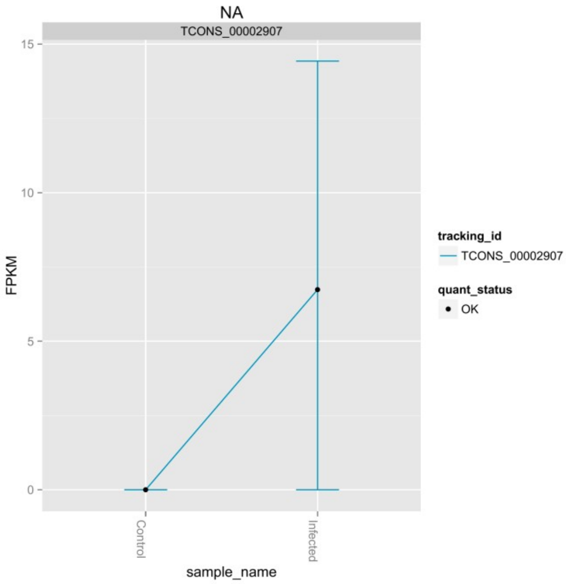
NA
TCONS_00002907
15
10
5
0
FPKM
Control
Infected
sample_name
tracking_id
TCONS_00002907
quant_status
OK

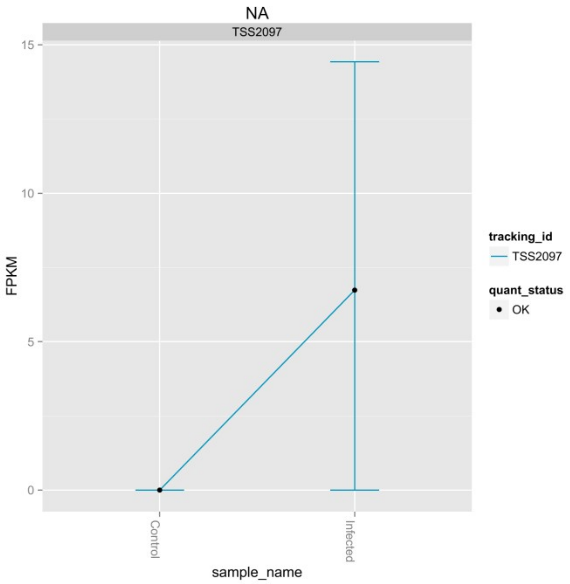
NA
TSS2097
15
10
5
0
FPKM
Control
Infected
sample_name
tracking_id
TSS2097
quant_status
OK

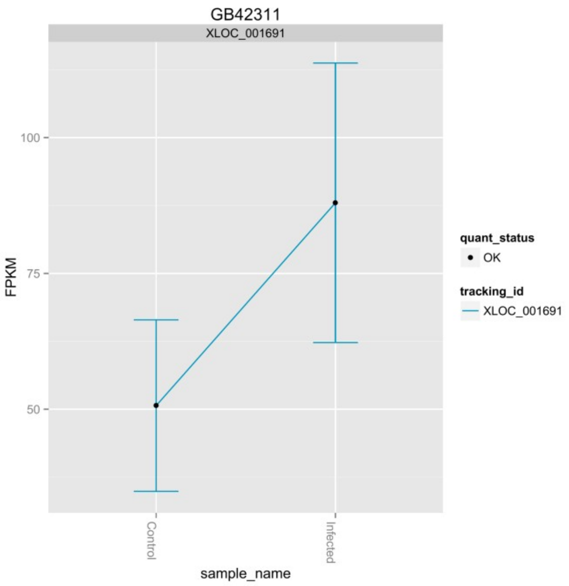
GB42311
XLOC_001691
100
75
50
FPKM
Control
Infected
sample_name
quant_status
OK
tracking_id
XLOC_001691

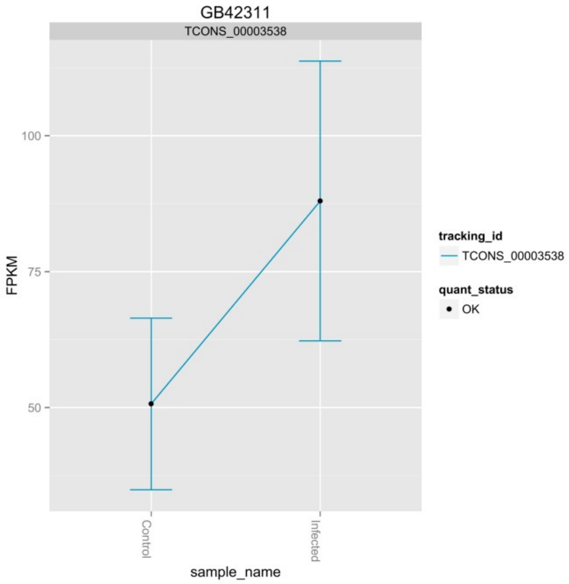
GB42311
TCONS_00003538
100
75
50
FPKM
Control
Infected
sample_name
tracking_id
TCONS_00003538
quant_status
OK

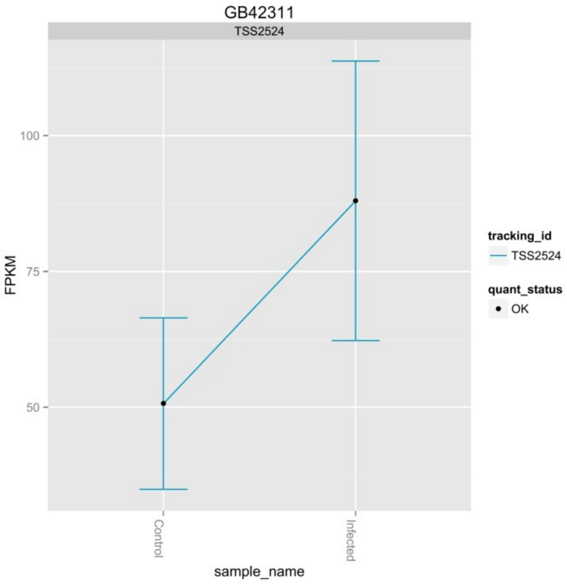
GB42311
TSS2524
100
75
50
FPKM
Control
Infected
sample_name
tracking_id
TSS2524
quant_status
OK

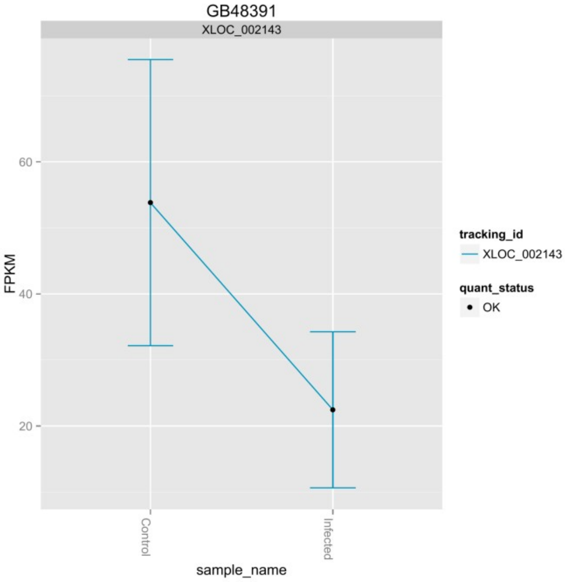
GB48391
XLOC_002143
60
40
20
FPKM
Control
Infected
sample_name
tracking_id
XLOC_002143
quant_status
OK

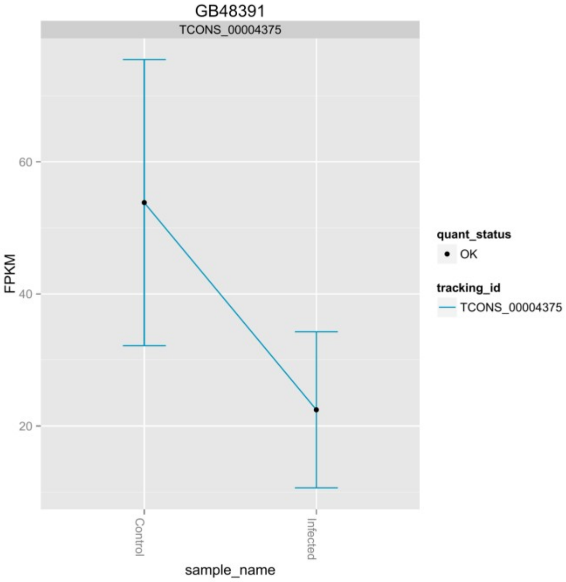
GB48391
TCONS_00004375
60
40
20
FPKM
Control
Infected
sample_name
quant_status
OK
tracking_id
TCONS_00004375

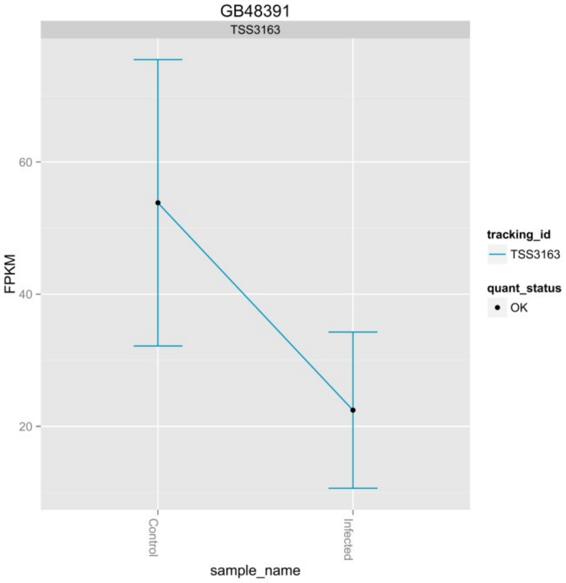
GB48391
TSS3163
60
40
20
FPKM
Control
Infected
sample_name
tracking_id
TSS3163
quant_status
OK

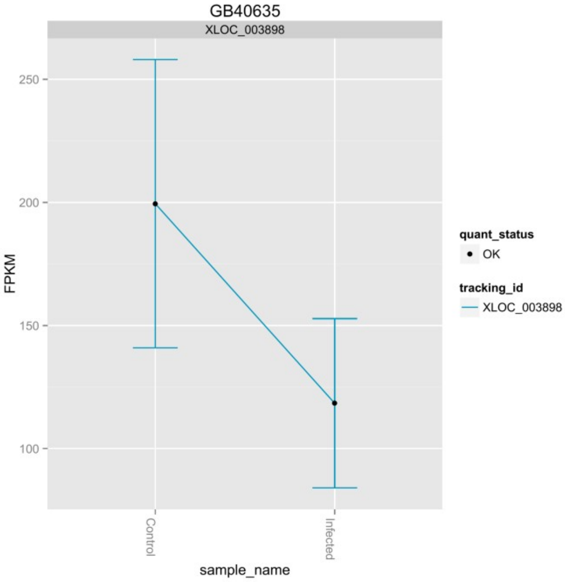
GB40635
XLOC_003898
250
200
150
100
FPKM
Control
Infected
sample_name
quant_status
OK
tracking_id
XLOC_003898

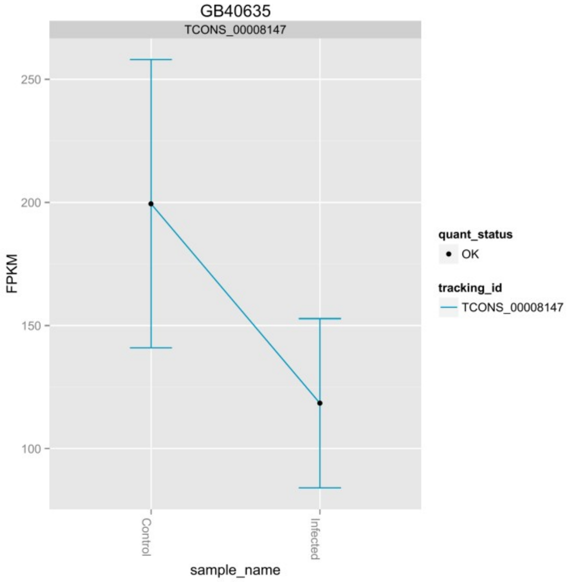
GB40635
TCONS_00008147
250
200
150
100
FPKM
Control
Infected
sample_name
quant_status
OK
tracking_id
TCONS_00008147

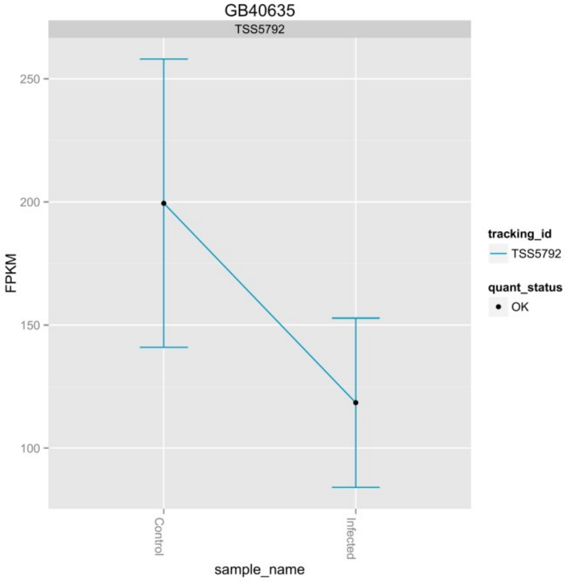
GB40635
TSS5792
250
200
150
100
FPKM
Control
Infected
sample_name
tracking_id
TSS5792
quant_status
OK

Group 2: spliced and transcriptionally regulated genes

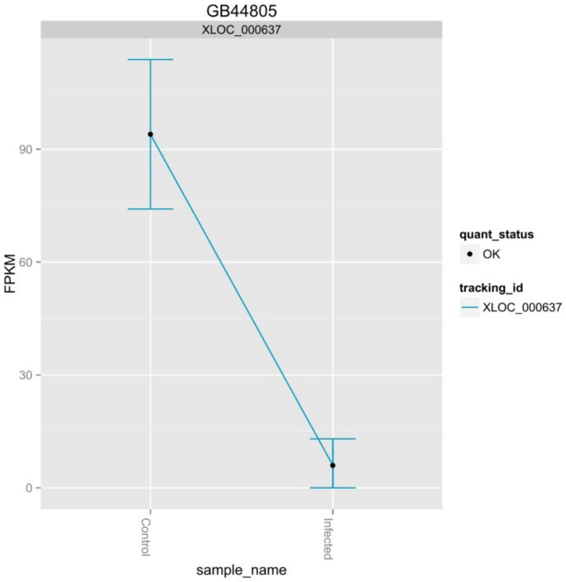
GB44805
XLOC_000637
FPKM
90
60
30
0
Control
Infected
sample_name
quant_status
OK
tracking_id
XLOC_000637

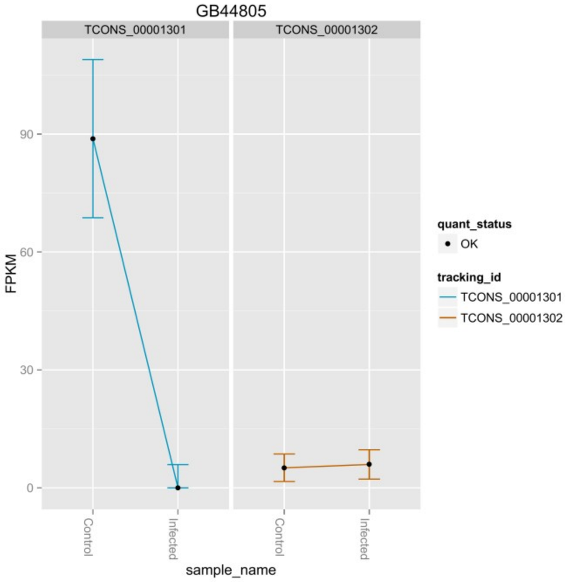
GB44805
TCONS_00001301
TCONS_00001302
FPKM
90
60
30
0
Control
Infected
Control
Infected
sample_name
quant_status
OK
tracking_id
TCONS_00001301
TCONS_00001302

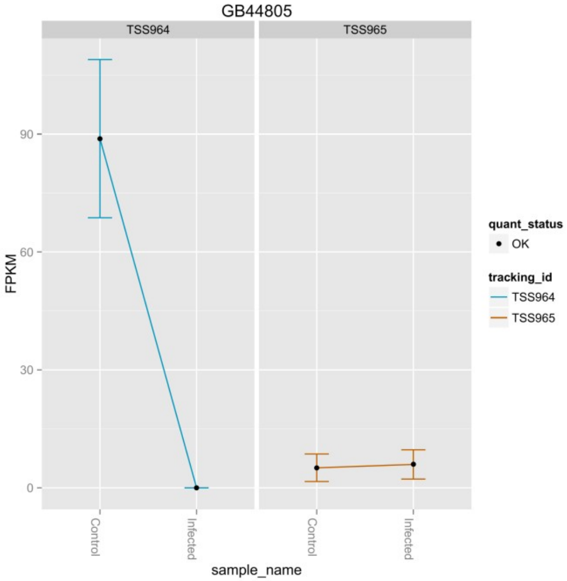
GB44805
TSS964
TSS965
FPKM
90
60
30
0
Control
Infected
Control
Infected
sample_name
quant_status
OK
tracking_id
TSS964
TSS965

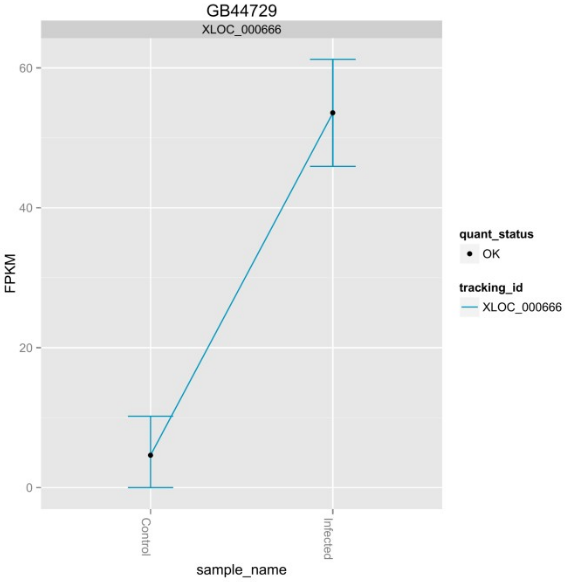

GB44729
XLOC_000666
FPKM
0
20
40
60
Control
Infected
sample_name
quant_status
OK
tracking_id
XLOC_000666

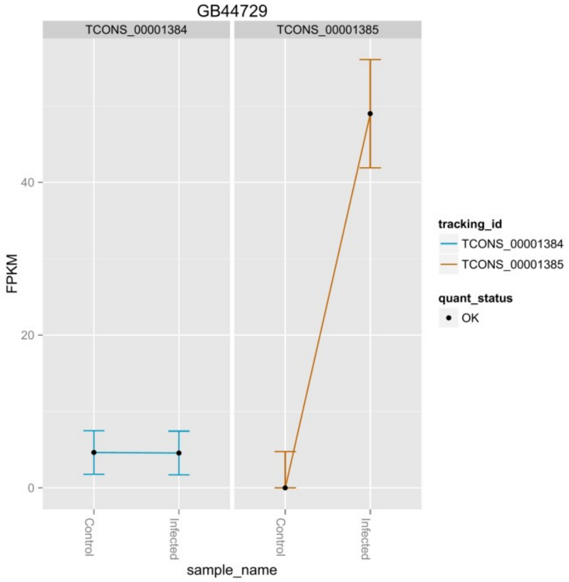

GB44729
TCONS_00001384
TCONS_00001385
FPKM
0
20
40
Control
Infected
Control
Infected
sample_name
tracking_id
TCONS_00001384
TCONS_00001385
quant_status
OK

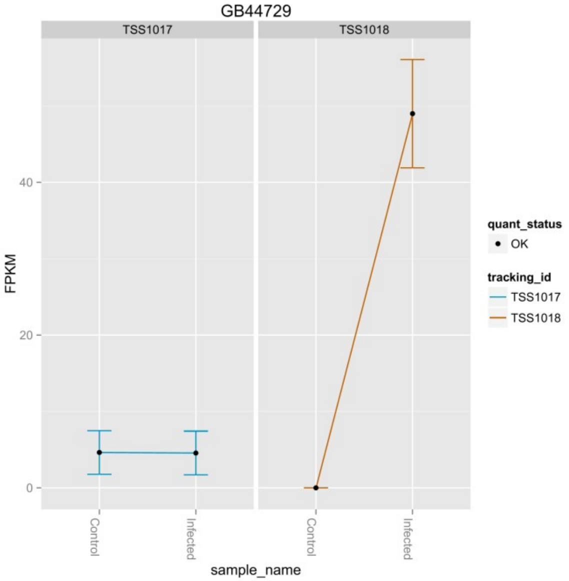

GB44729
TSS1017
TSS1018
FPKM
0
20
40
Control
Infected
Control
Infected
sample_name
quant_status
OK
tracking_id
TSS1017
TSS1018

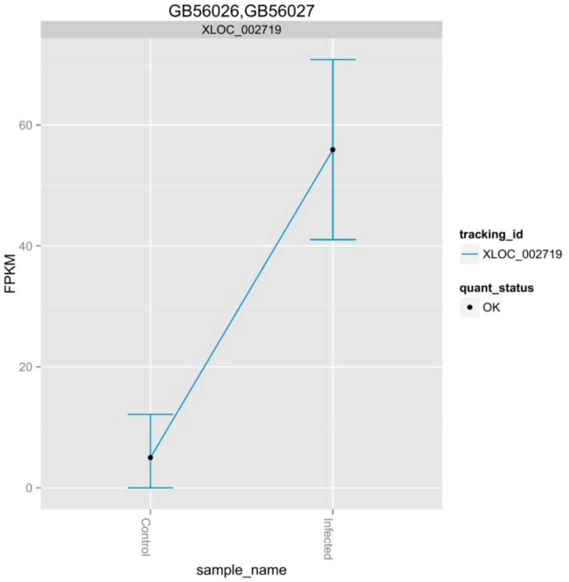
GB56026,GB56027
XLOC_002719
FPKM
0
20
40
60
Control
Infected
sample_name
tracking_id
XLOC_002719
quant_status
OK

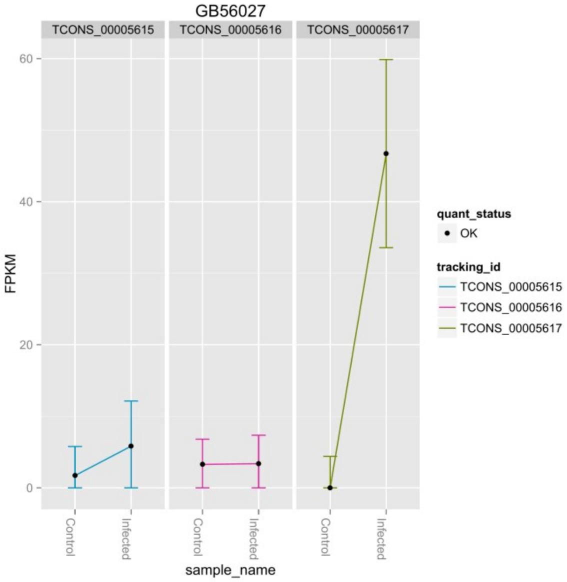
GB56027
TCONS_00005615
TCONS_00005616
TCONS_00005617
FPKM
0
20
40
60
Control
Infected
sample_name
quant_status
OK
tracking_id
TCONS_00005615
TCONS_00005616
TCONS_00005617

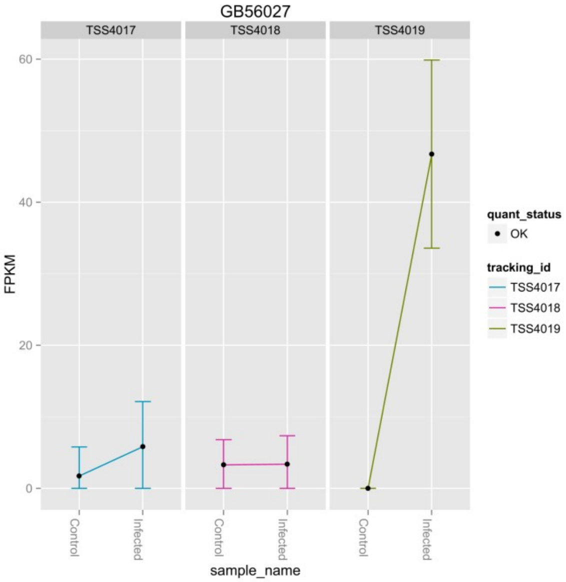
GB56027
TSS4017
TSS4018
TSS4019
FPKM
0
20
40
60
Control
Infected
sample_name
quant_status
OK
tracking_id
TSS4017
TSS4018
TSS4019

Group 3:
Spliced and both transcriptionally and post-transcriptionally regulated

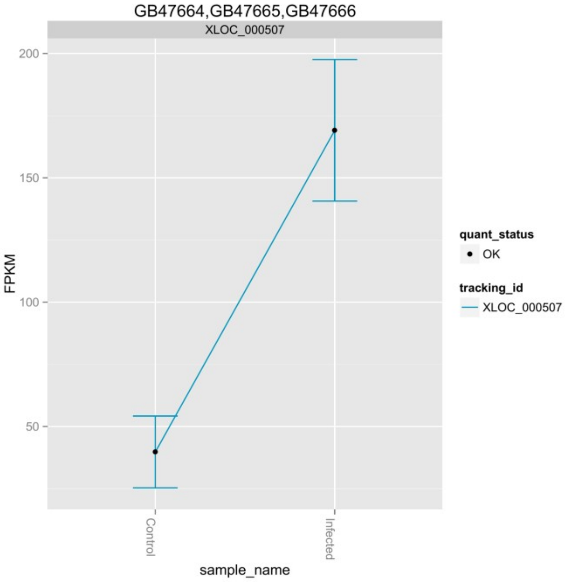
GB47664,GB47665,GB47666
XLOC_000507
FPKM
200
150
100
50
Control
Infected
sample_name
quant_status
OK
tracking_id
XLOC_000507

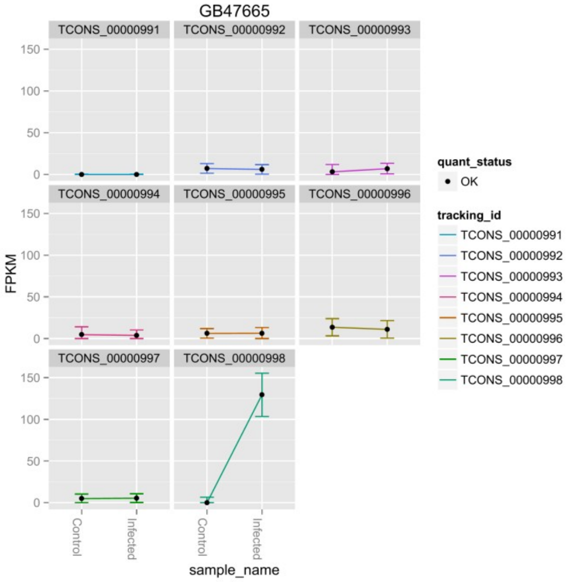
GB47665
TCONS_00000991
TCONS_00000992
TCONS_00000993
TCONS_00000994
TCONS_00000995
TCONS_00000996
TCONS_00000997
TCONS_00000998
FPKM
150
100
50
0
Control
Infected
sample_name
quant_status
OK
tracking_id
TCONS_00000991
TCONS_00000992
TCONS_00000993
TCONS_00000994
TCONS_00000995
TCONS_00000996
TCONS_00000997
TCONS_00000998

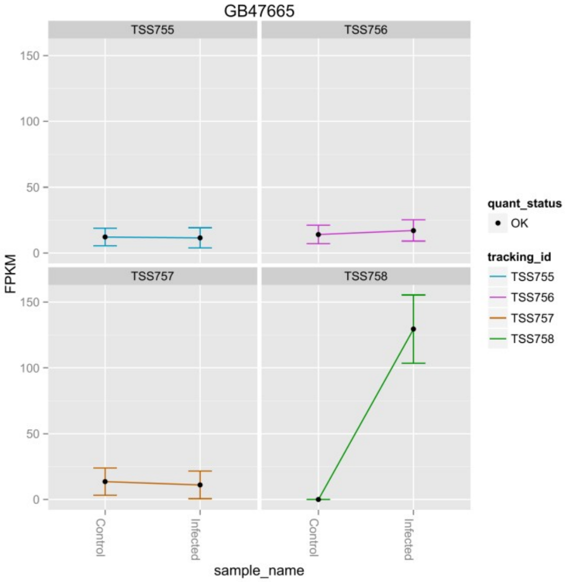
GB47665
TSS755
TSS756
TSS757
TSS758
FPKM
150
100
50
0
Control
Infected
sample_name
quant_status
OK
tracking_id
TSS755
TSS756
TSS757
TSS758

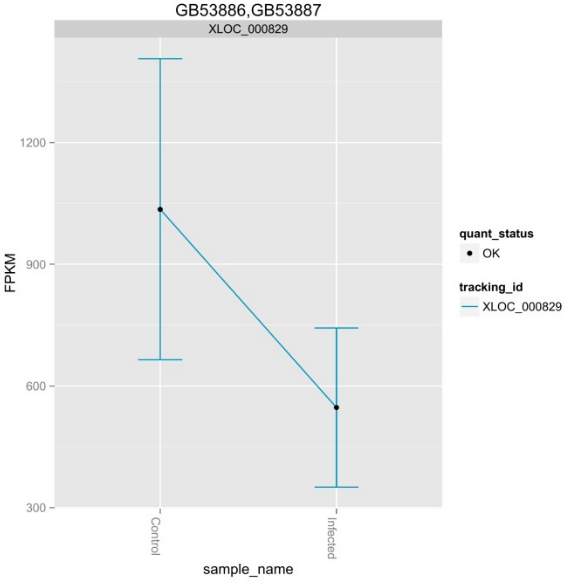
GB53886,GB53887
XLOC_000829
FPKM
1200
900
600
300
Control
Infected
sample_name
quant_status
OK
tracking_id
XLOC_000829

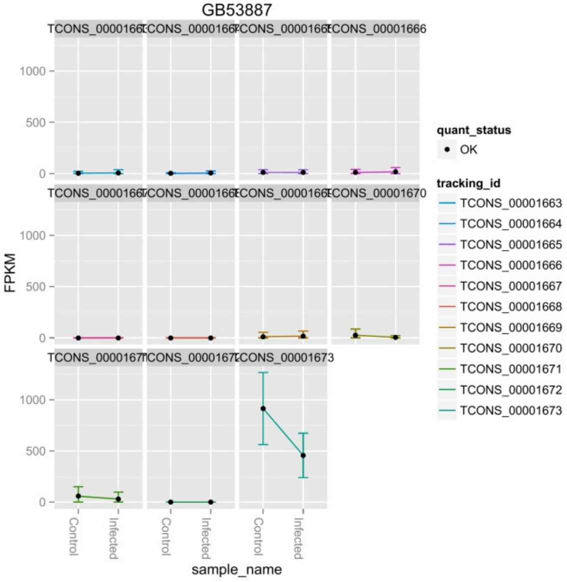
GB53887
TCONS_00001666
TCONS_00001670
TCONS_00001673
FPKM
1000
500
0
Control
Infected
sample_name
quant_status
OK
tracking_id
TCONS_00001663
TCONS_00001664
TCONS_00001665
TCONS_00001666
TCONS_00001667
TCONS_00001668
TCONS_00001669
TCONS_00001670
TCONS_00001671
TCONS_00001672
TCONS_00001673

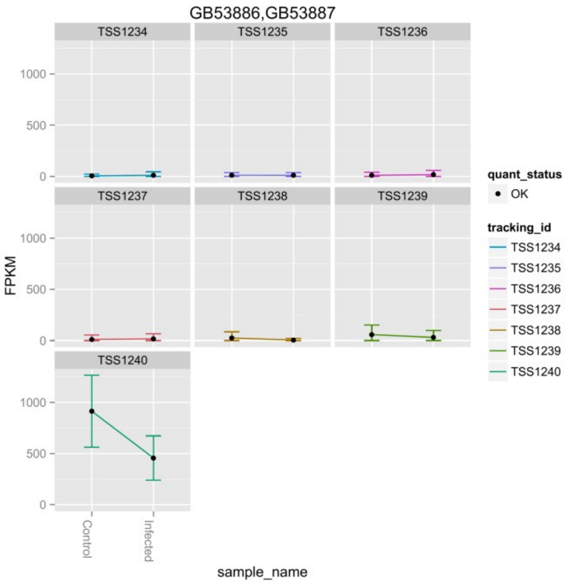
GB53886,GB53887
TSS1234
TSS1235
TSS1236
TSS1237
TSS1238
TSS1239
TSS1240
FPKM
1000
500
0
Control
Infected
sample_name
quant_status
OK
tracking_id
TSS1234
TSS1235
TSS1236
TSS1237
TSS1238
TSS1239
TSS1240

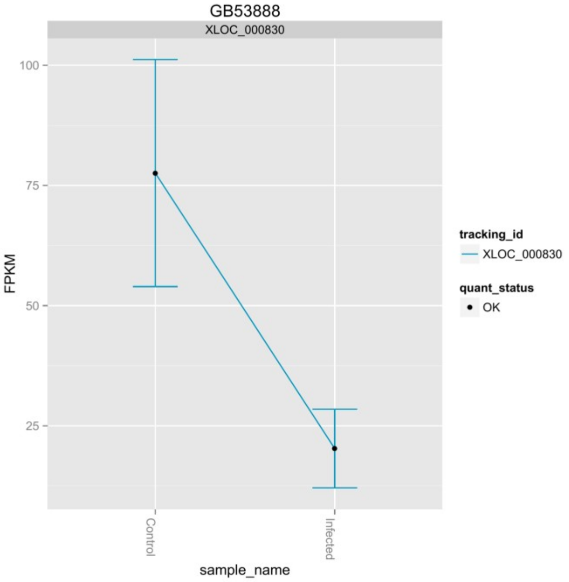
GB53888
XLOC_000830
100
75
50
25
FPKM
Control
Infected
sample_name
tracking_id
XLOC_000830
quant_status
OK

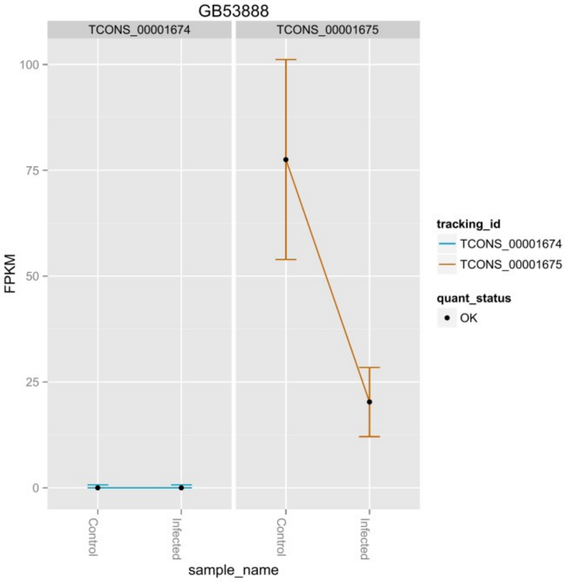
GB53888
TCONS_00001674
TCONS_00001675
100
75
50
25
0
FPKM
Control
Infected
Control
Infected
sample_name
tracking_id
TCONS_00001674
TCONS_00001675
quant_status
OK

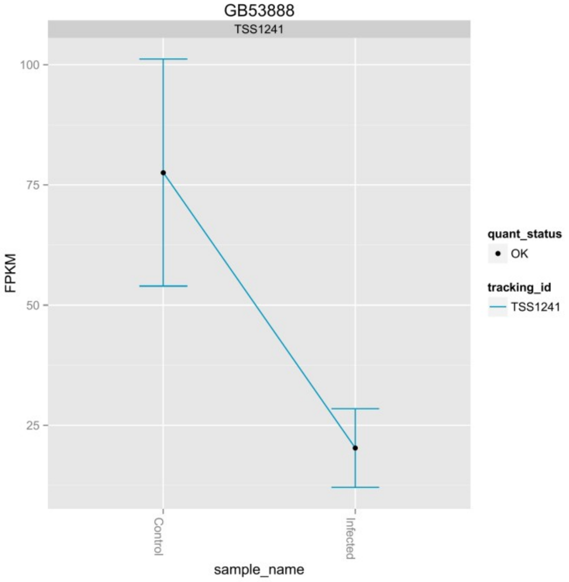
GB53888
TSS1241
100
75
50
25
FPKM
Control
Infected
sample_name
quant_status
OK
tracking_id
TSS1241

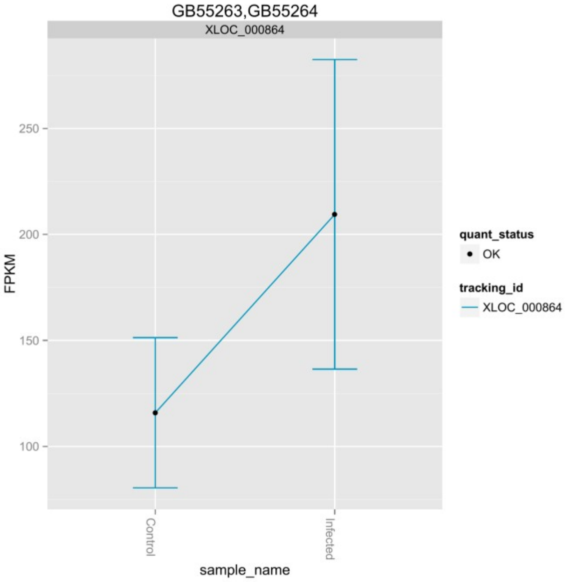
GB55263,GB55264
XLOC_000864
FPKM
250
200
150
100
Control
Infected
sample_name
quant_status
OK
tracking_id
XLOC_000864

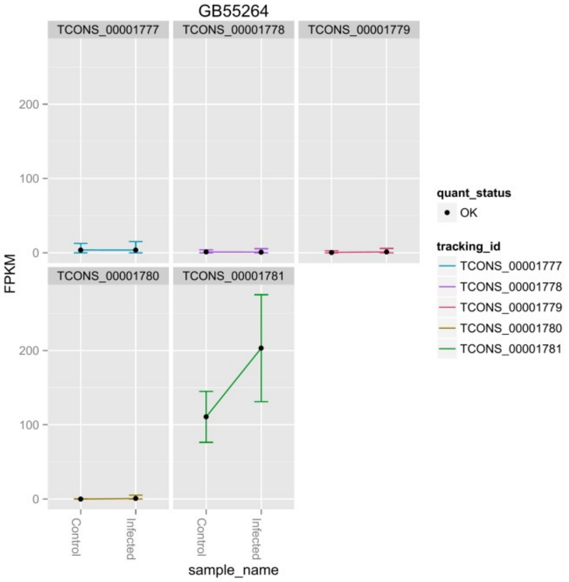
GB55264
TCONS_00001777
TCONS_00001778
TCONS_00001779
TCONS_00001780
TCONS_00001781
FPKM
200
100
0
Control
Infected
sample_name
quant_status
OK
tracking_id
TCONS_00001777
TCONS_00001778
TCONS_00001779
TCONS_00001780
TCONS_00001781

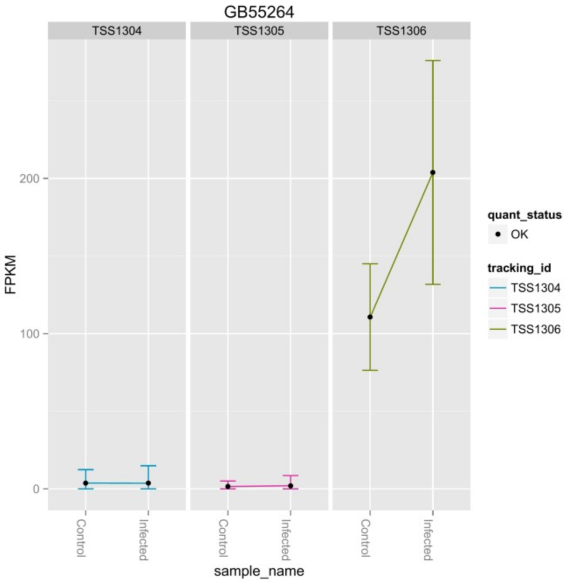
GB55264
TSS1304
TSS1305
TSS1306
FPKM
200
100
0
Control
Infected
sample_name
quant_status
OK
tracking_id
TSS1304
TSS1305
TSS1306

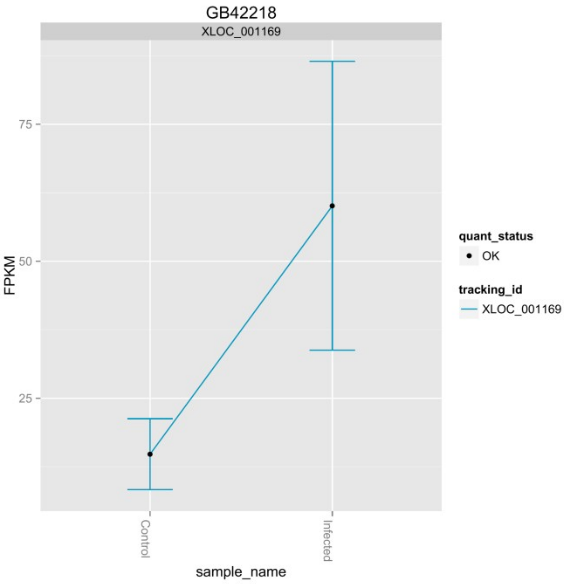
GB42218
XLOC_001169
75
50
25
FPKM
Control
Infected
sample_name
quant_status
OK
tracking_id
XLOC_001169

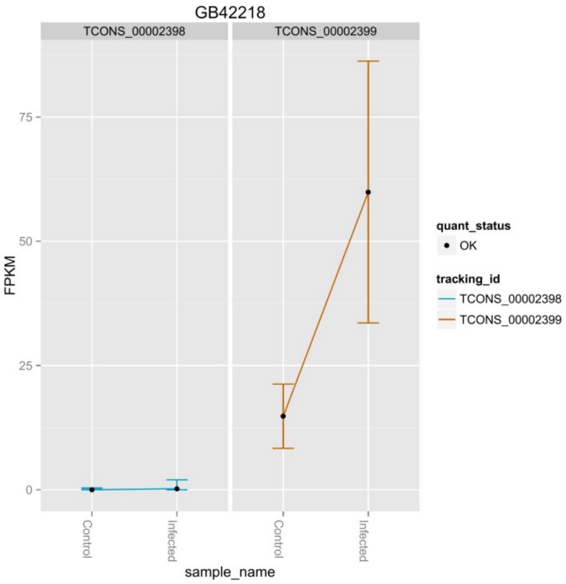
GB42218
TCONS_00002398
TCONS_00002399
75
50
25
0
FPKM
Control
Infected
Control
Infected
sample_name
quant_status
OK
tracking_id
TCONS_00002398
TCONS_00002399

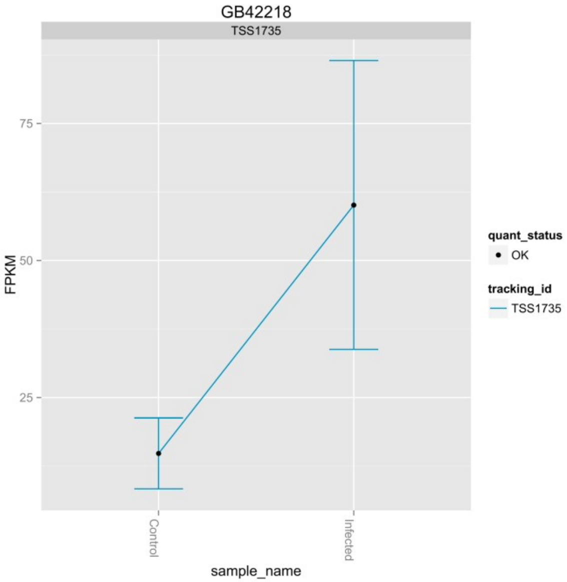
GB42218
TSS1735
75
50
25
FPKM
Control
Infected
sample_name
quant_status
OK
tracking_id
TSS1735

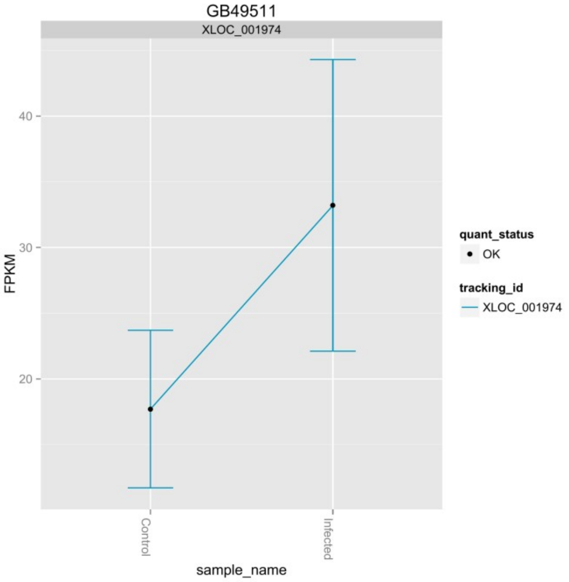
GB49511
XLOC_001974
FPKM
40
30
20
Control
Infected
sample_name
quant_status
OK
tracking_id
XLOC_001974

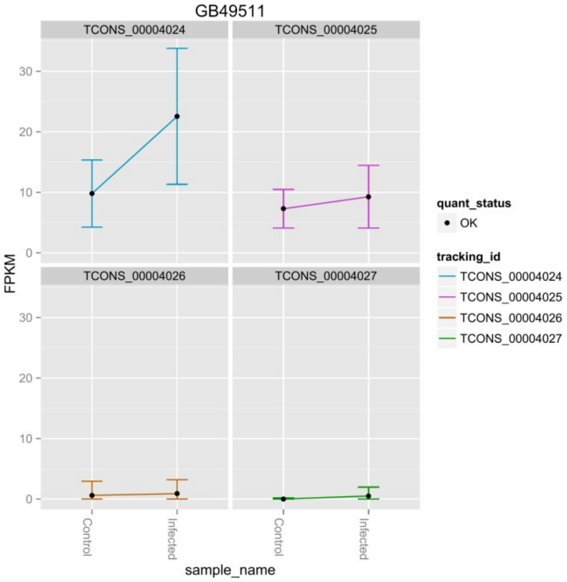
GB49511
TCONS_00004024
TCONS_00004025
TCONS_00004026
TCONS_00004027
FPKM
30
20
10
0
Control
Infected
sample_name
quant_status
OK
tracking_id
TCONS_00004024
TCONS_00004025
TCONS_00004026
TCONS_00004027

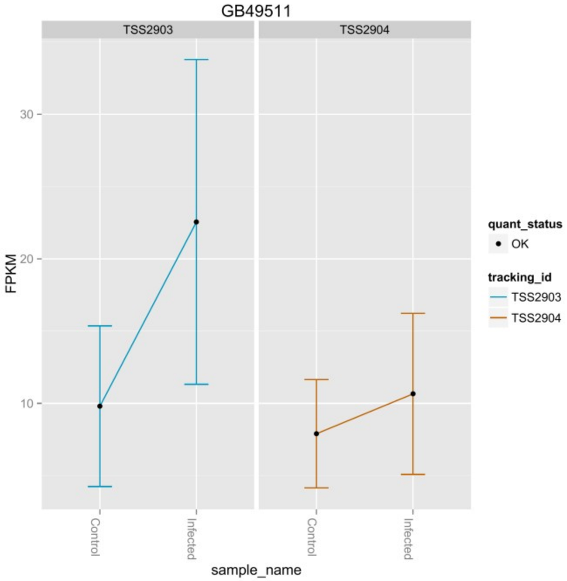
GB49511
TSS2903
TSS2904
FPKM
30
20
10
Control
Infected
sample_name
quant_status
OK
tracking_id
TSS2903
TSS2904

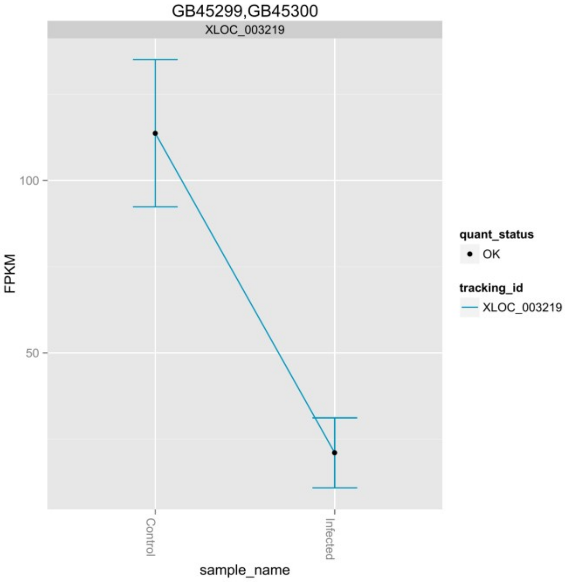
GB45299,GB45300
XLOC_003219
100
50
FPKM
Control
Infected
sample_name
quant_status
OK
tracking_id
XLOC_003219

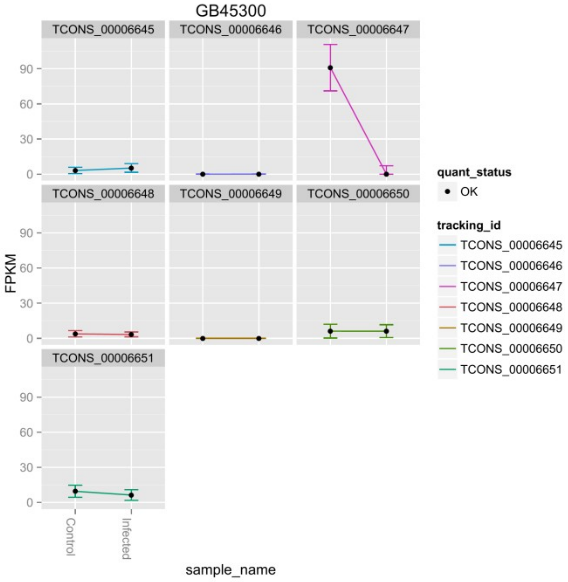
GB45300
TCONS_00006645
TCONS_00006646
TCONS_00006647
TCONS_00006648
TCONS_00006649
TCONS_00006650
TCONS_00006651
90
60
30
0
FPKM
Control
Infected
sample_name
quant_status
OK
tracking_id
TCONS_00006645
TCONS_00006646
TCONS_00006647
TCONS_00006648
TCONS_00006649
TCONS_00006650
TCONS_00006651

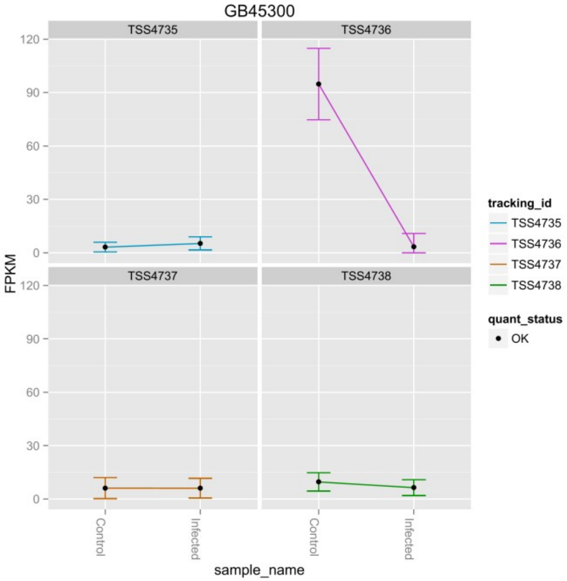
GB45300
TSS4735
TSS4736
TSS4737
TSS4738
120
90
60
30
0
FPKM
Control
Infected
sample_name
tracking_id
TSS4735
TSS4736
TSS4737
TSS4738
quant_status
OK
