# Supplementary material for: RNA-sequence analysis of gene expression from honeybees (Apis mellifera) infected with Nosema ceranae
Source: PLoS One. 2017 Mar 28;12(3):e0173438. doi: 10.1371/journal.pone.0173438 (PMC5370102; doi:10.1371/journal.pone.0173438)
Supplement: S7 Fig — (Group 1) Un-spliced and transcriptionally regulated genes,(Group 2) spliced and post-transcriptionally regulated genes and(Group 3) spliced and both transcriptionally and post-transcriptionally regulated genes. For each transcript, the “XLOC”, “TSS” and “TCONS” suffixes correspond to the genes, TSSs and isoforms, respectively. Differentially expressed isoforms with different TSSs are transcriptionally regulated, while isoforms with the same TSS are regulated at the post-transcriptional level. (PDF) [file pone.0173438.s012.pdf]

Group 1: un-spliced and transcriptionally regulated genes

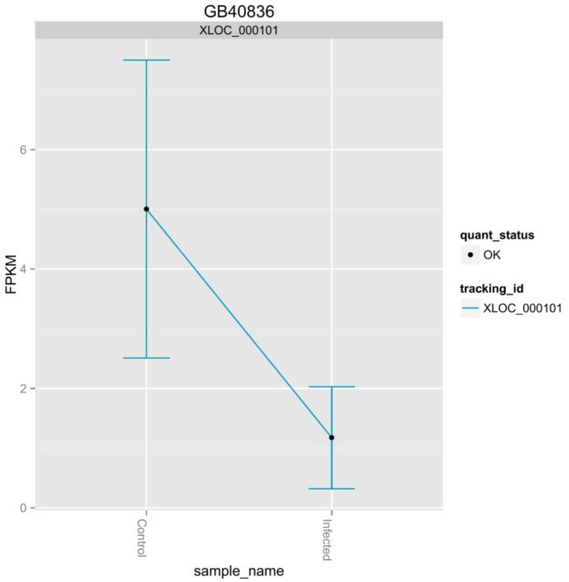
GB40836
XLOC_000101
FPKM
0
2
4
6
Control
Infected
sample_name
quant_status
OK
tracking_id
XLOC_000101

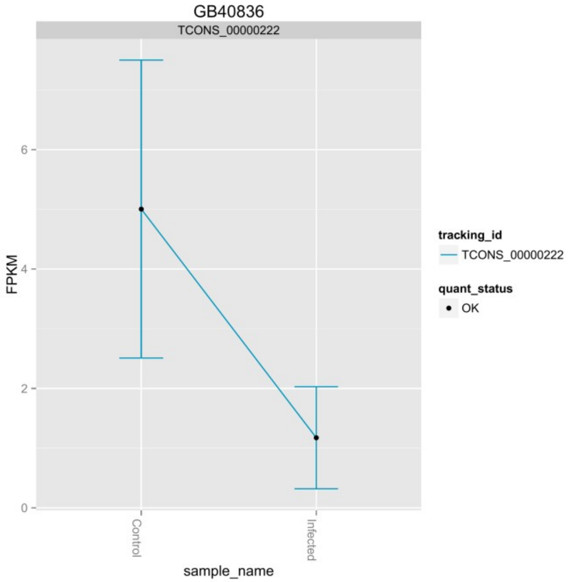
GB40836
TCONS_00000222
FPKM
0
2
4
6
Control
Infected
sample_name
tracking_id
TCONS_00000222
quant_status
OK

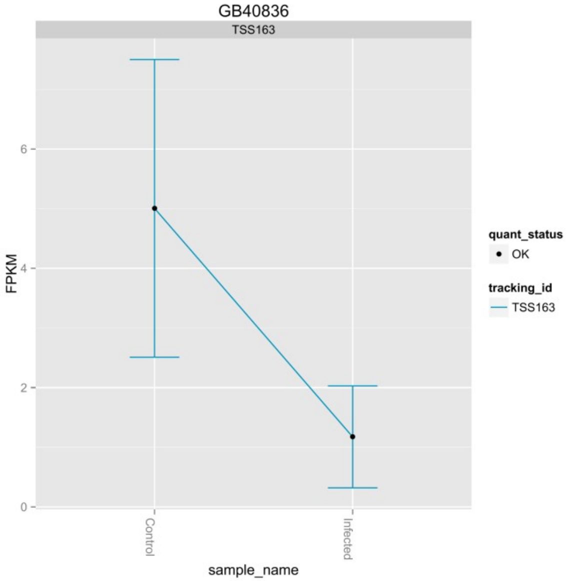
GB40836
TSS163
FPKM
0
2
4
6
Control
Infected
sample_name
quant_status
OK
tracking_id
TSS163

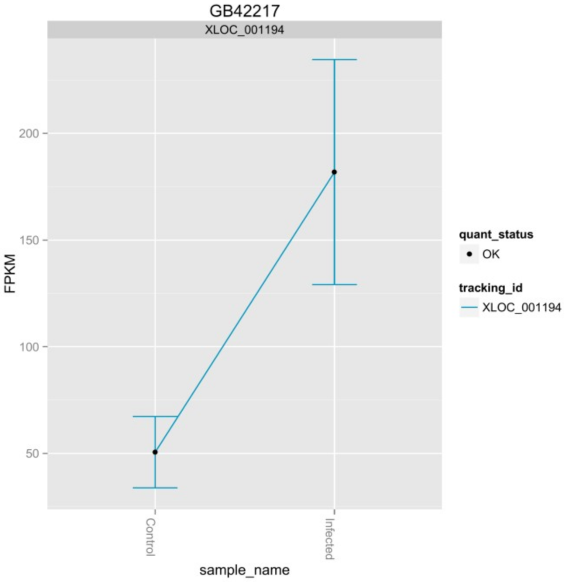
GB42217
XLOC_001194
FPKM
200
150
100
50
Control
Infected
sample_name
quant_status
OK
tracking_id
XLOC_001194

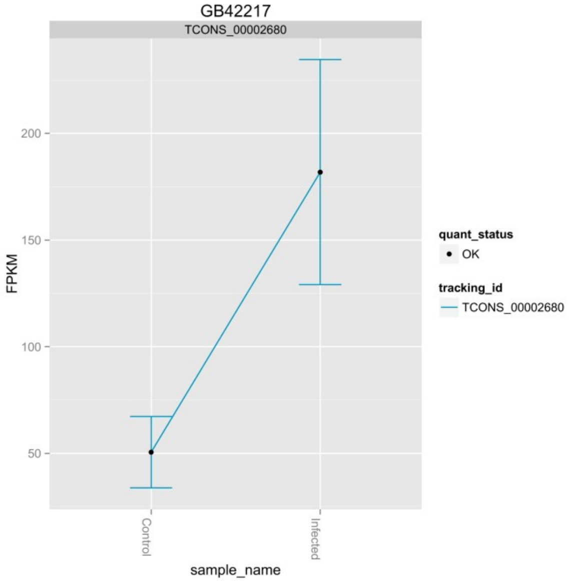
GB42217
TCONS_00002680
FPKM
200
150
100
50
Control
Infected
sample_name
quant_status
OK
tracking_id
TCONS_00002680

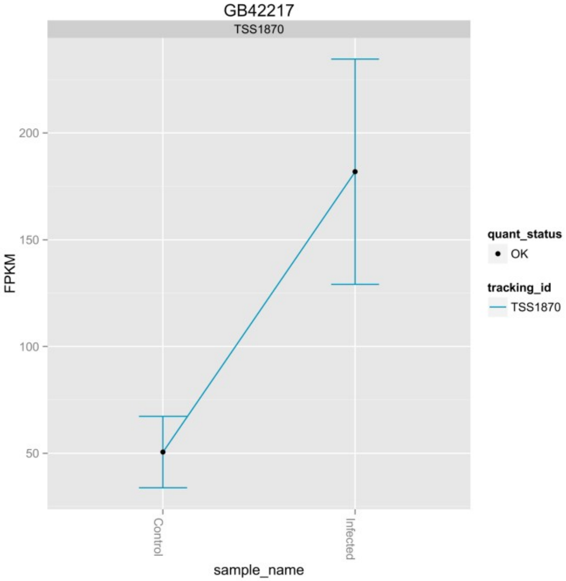
GB42217
TSS1870
FPKM
200
150
100
50
Control
Infected
sample_name
quant_status
OK
tracking_id
TSS1870

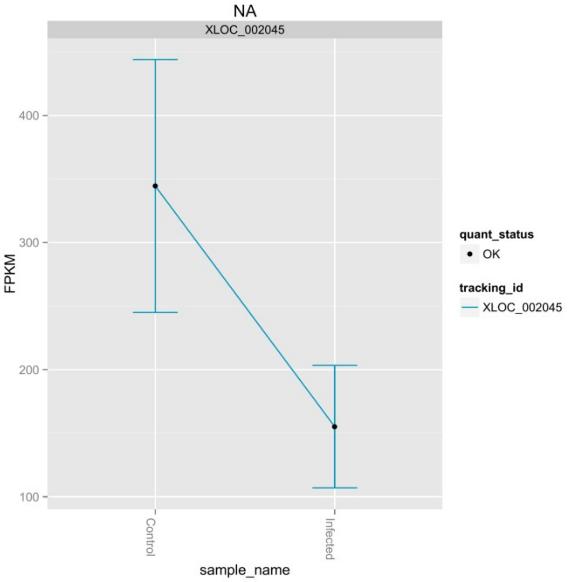
NA
XLOC_002045
FPKM
400
300
200
100
Control
Infected
sample_name
quant_status
OK
tracking_id
XLOC_002045

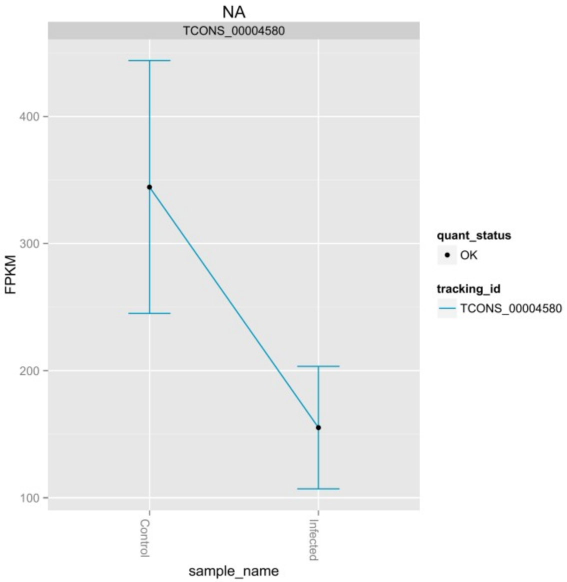
NA
TCONS_00004580
FPKM
400
300
200
100
Control
Infected
sample_name
quant_status
OK
tracking_id
TCONS_00004580

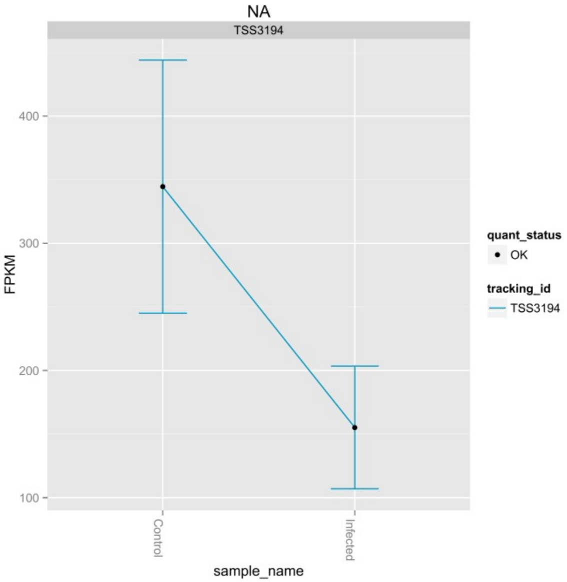
NA
TSS3194
FPKM
400
300
200
100
Control
Infected
sample_name
quant_status
OK
tracking_id
TSS3194

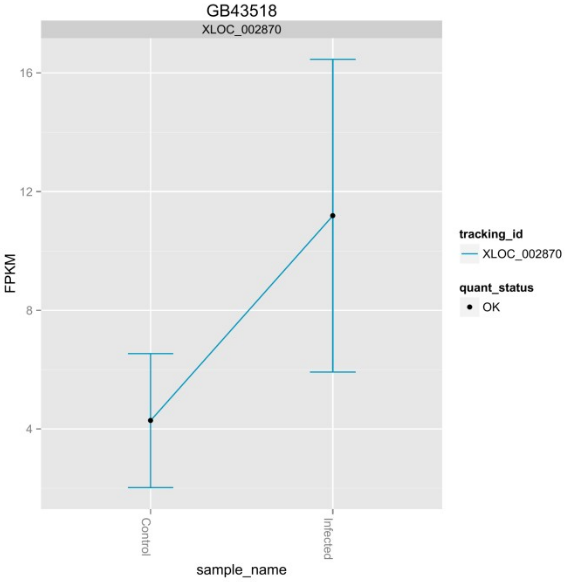
GB43518
XLOC_002870
FPKM
16
12
8
4
Control
Infected
sample_name
tracking_id
XLOC_002870
quant_status
OK

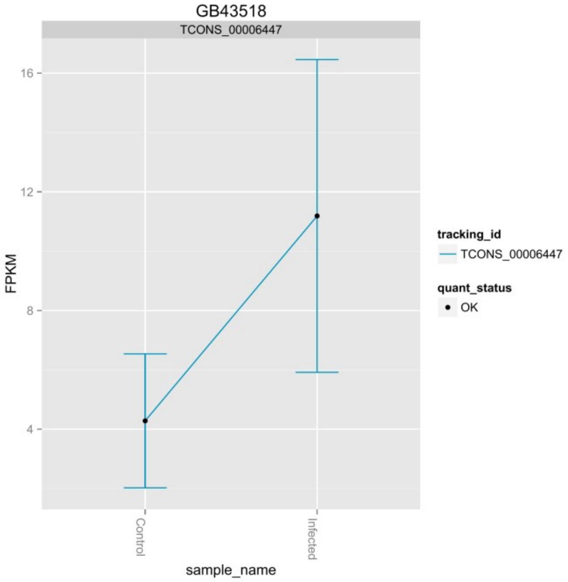
GB43518
TCONS_00006447
FPKM
16
12
8
4
Control
Infected
sample_name
tracking_id
TCONS_00006447
quant_status
OK

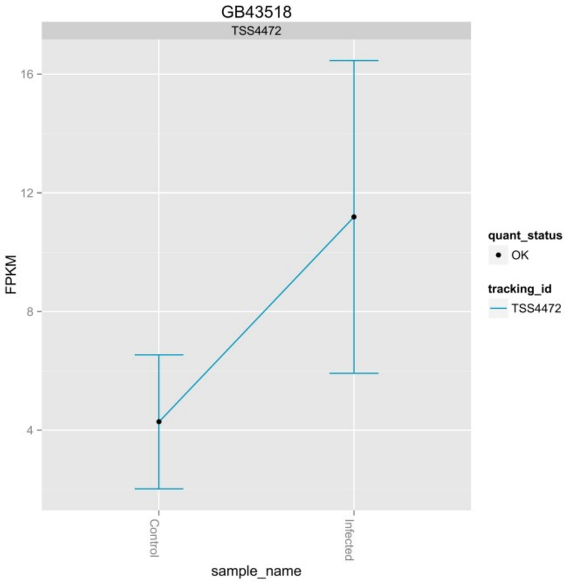
GB43518
TSS4472
FPKM
16
12
8
4
Control
Infected
sample_name
quant_status
OK
tracking_id
TSS4472

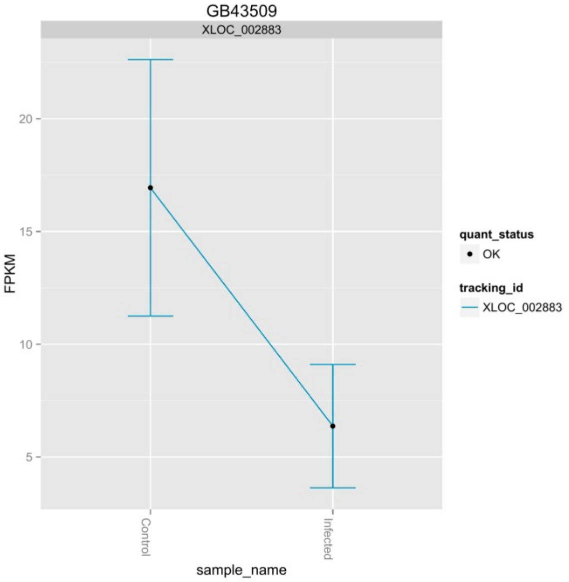

GB43509
XLOC_002883
FPKM
20
15
10
5
Control
Infected
sample_name
quant_status
OK
tracking_id
XLOC_002883

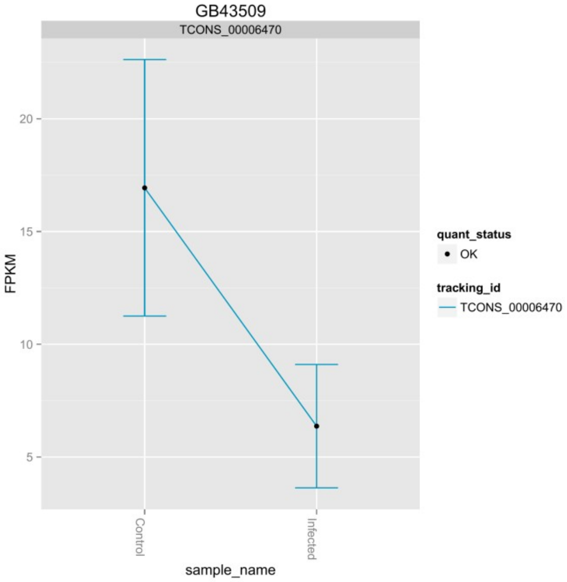

GB43509
TCONS_00006470
FPKM
20
15
10
5
Control
Infected
sample_name
quant_status
OK
tracking_id
TCONS_00006470

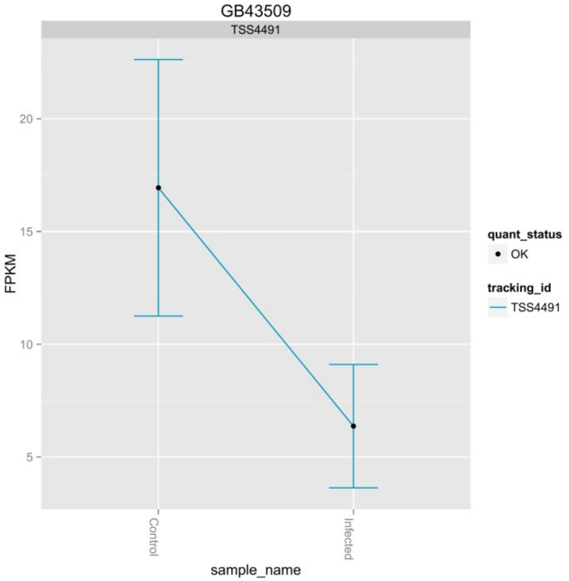

GB43509
TSS4491
FPKM
20
15
10
5
Control
Infected
sample_name
quant_status
OK
tracking_id
TSS4491

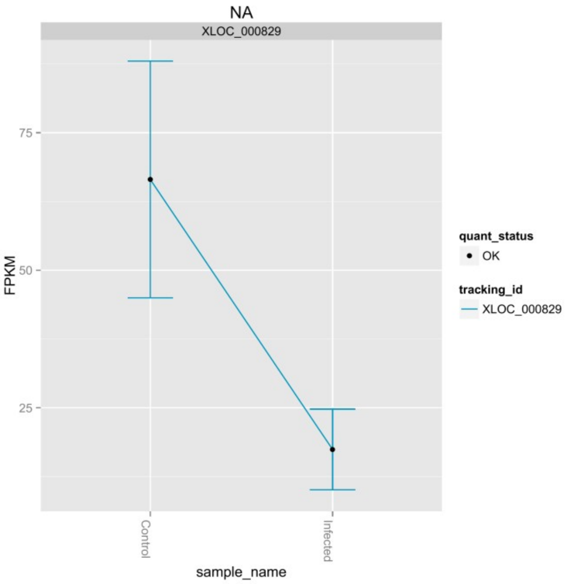
NA
XLOC_000829
75
50
25
FPKM
Control
Infected
sample_name
quant_status
OK
tracking_id
XLOC_000829

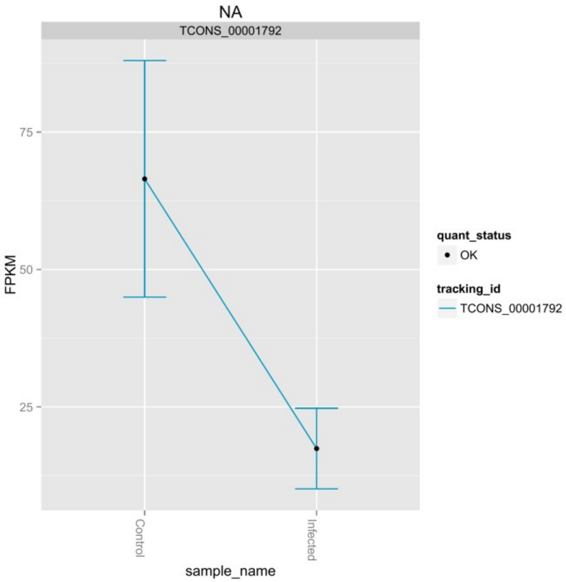
NA
TCONS_00001792
75
50
25
FPKM
Control
Infected
sample_name
quant_status
OK
tracking_id
TCONS_00001792

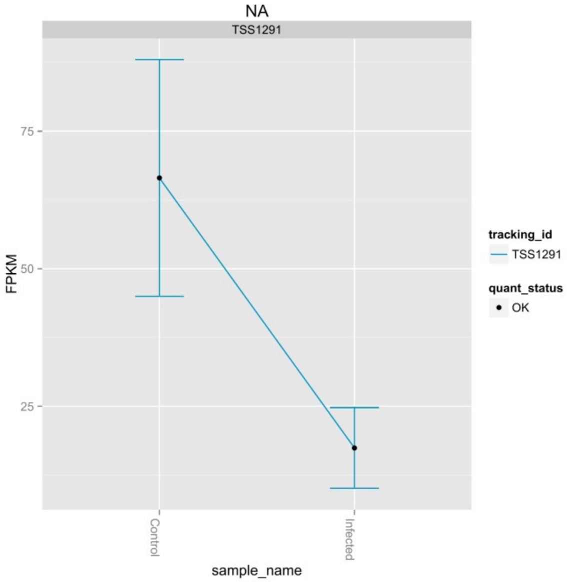
NA
TSS1291
75
50
25
FPKM
Control
Infected
sample_name
tracking_id
TSS1291
quant_status
OK

Group 2: spliced and transcriptionally regulated genes

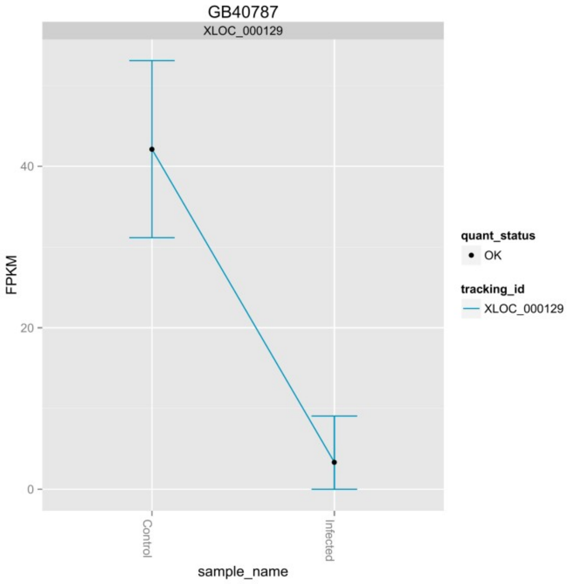
GB40787
XLOC_000129
FPKM
40
20
0
Control
Infected
sample_name
quant_status
OK
tracking_id
XLOC_000129

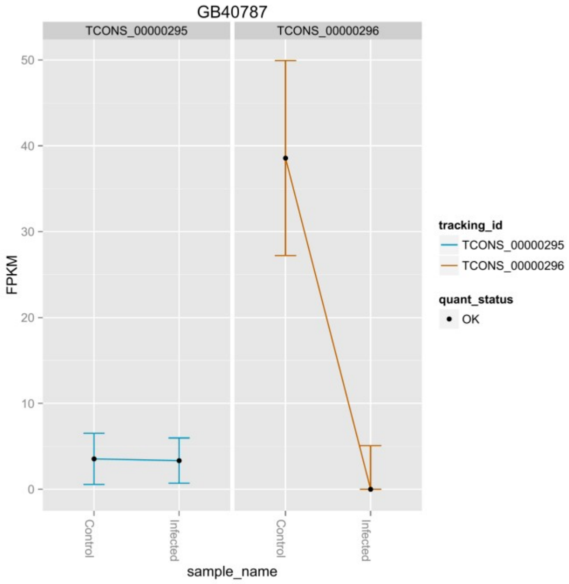
GB40787
TCONS_00000295
TCONS_00000296
FPKM
50
40
30
20
10
0
Control
Infected
Control
Infected
sample_name
tracking_id
TCONS_00000295
TCONS_00000296
quant_status
OK

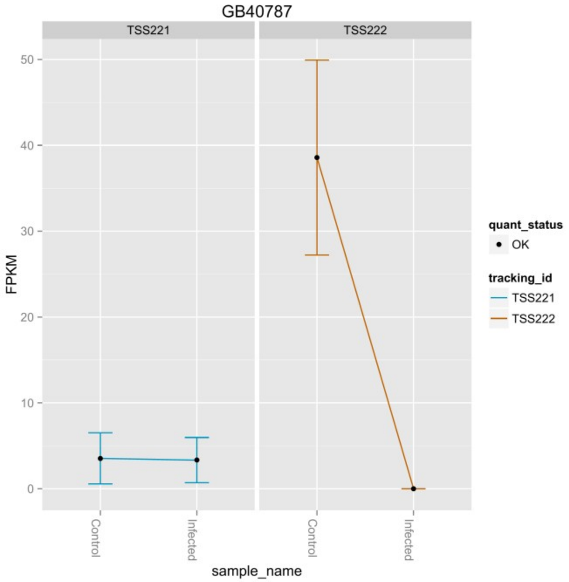
GB40787
TSS221
TSS222
FPKM
50
40
30
20
10
0
Control
Infected
Control
Infected
sample_name
quant_status
OK
tracking_id
TSS221
TSS222

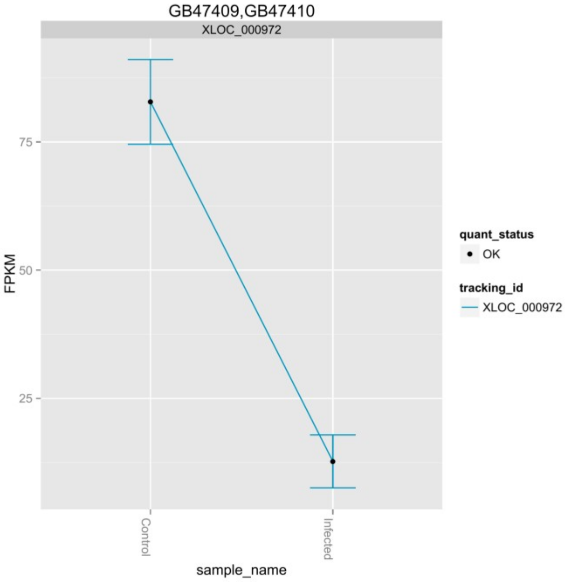
GB47409,GB47410
XLOC_000972
FPKM
75
50
25
Control
Infected
sample_name
quant_status
OK
tracking_id
XLOC_000972

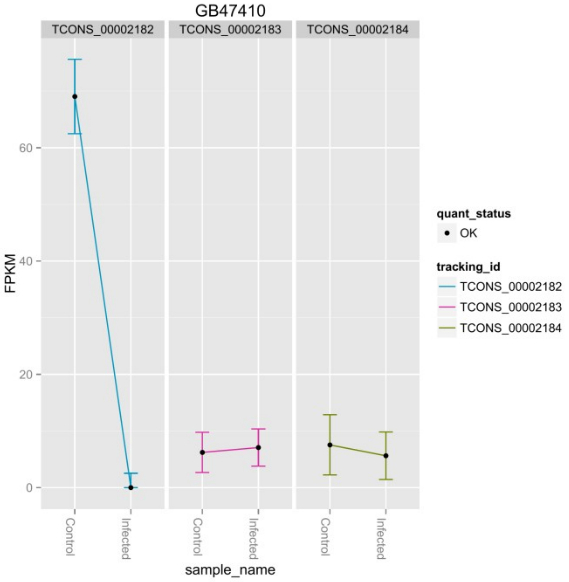
GB47410
TCONS_00002182
TCONS_00002183
TCONS_00002184
FPKM
60
40
20
0
Control
Infected
sample_name
quant_status
OK
tracking_id
TCONS_00002182
TCONS_00002183
TCONS_00002184

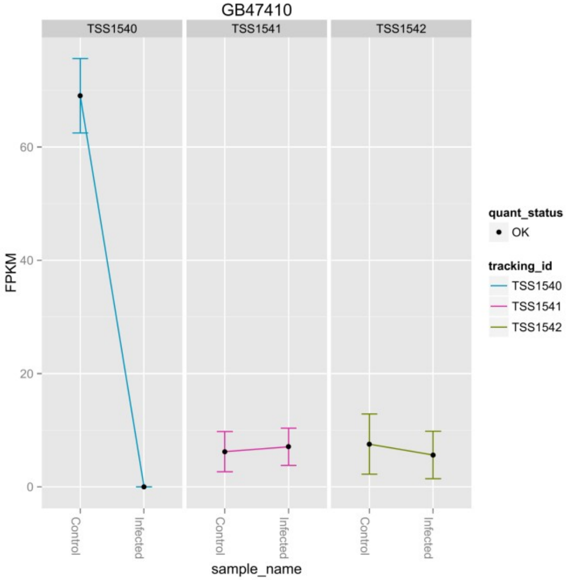
GB47410
TSS1540
TSS1541
TSS1542
FPKM
60
40
20
0
Control
Infected
sample_name
quant_status
OK
tracking_id
TSS1540
TSS1541
TSS1542

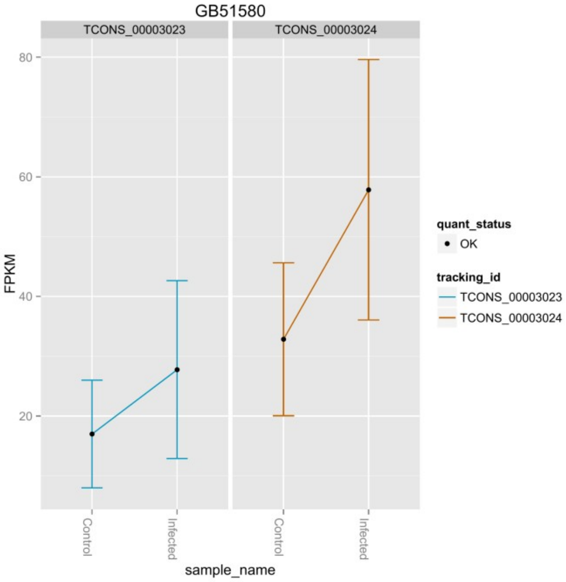
GB51580
TCONS_00003023
TCONS_00003024
80
60
40
20
FPKM
Control
Infected
Control
Infected
sample_name
quant_status
OK
tracking_id
TCONS_00003023
TCONS_00003024

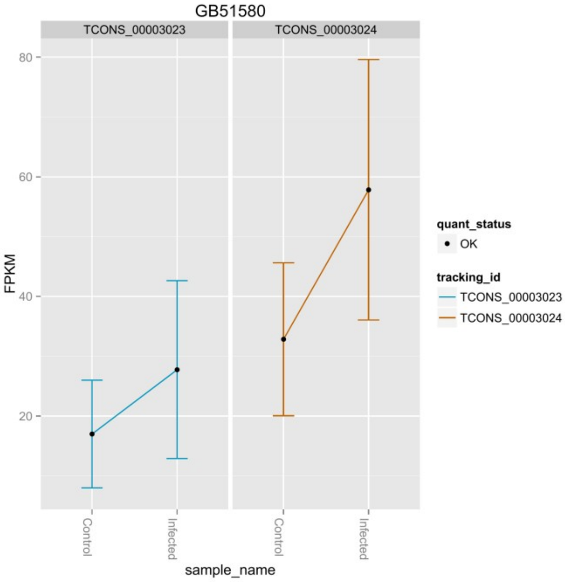
GB51580
TCONS_00003023
TCONS_00003024
80
60
40
20
FPKM
Control
Infected
Control
Infected
sample_name
quant_status
OK
tracking_id
TCONS_00003023
TCONS_00003024

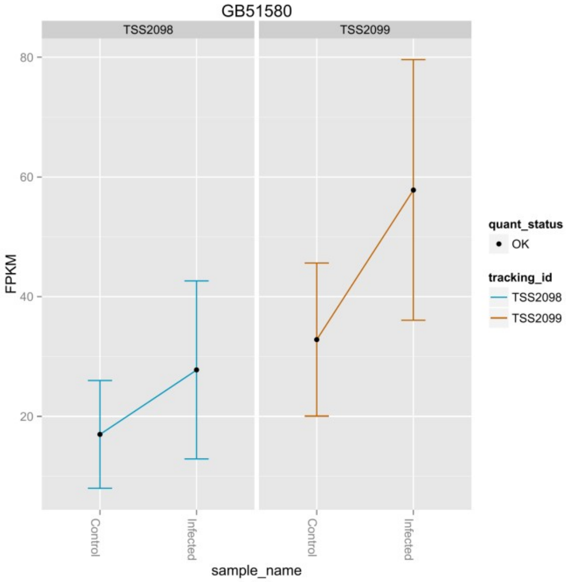
GB51580
TSS2098
TSS2099
80
60
40
20
FPKM
Control
Infected
Control
Infected
sample_name
quant_status
OK
tracking_id
TSS2098
TSS2099

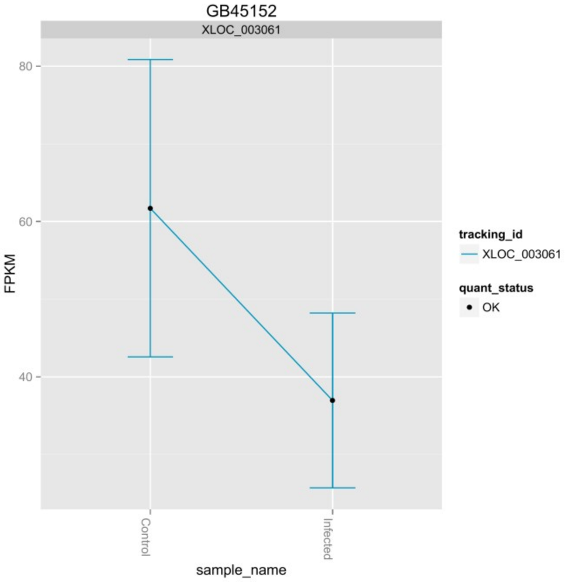
GB45152
XLOC_003061
80
60
40
FPKM
Control
Infected
sample_name
tracking_id
XLOC_003061
quant_status
OK

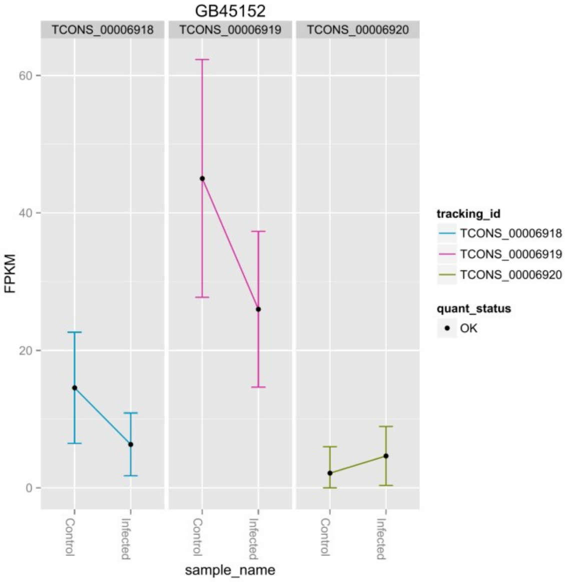
GB45152
TCONS_00006918
TCONS_00006919
TCONS_00006920
60
40
20
0
FPKM
Control
Infected
sample_name
tracking_id
TCONS_00006918
TCONS_00006919
TCONS_00006920
quant_status
OK

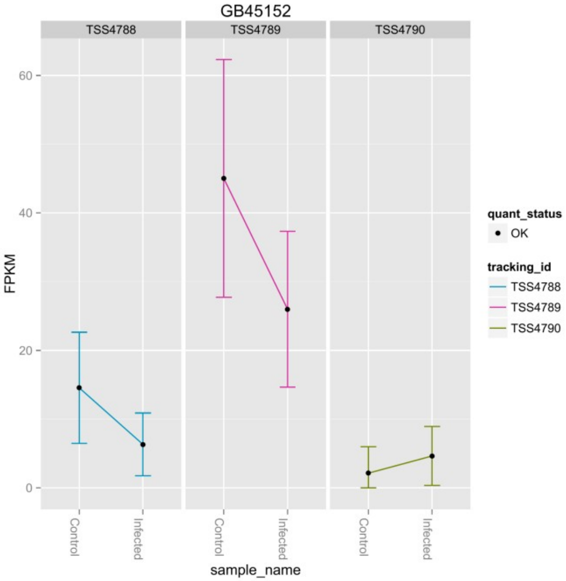
GB45152
TSS4788
TSS4789
TSS4790
60
40
20
0
FPKM
Control
Infected
sample_name
quant_status
OK
tracking_id
TSS4788
TSS4789
TSS4790

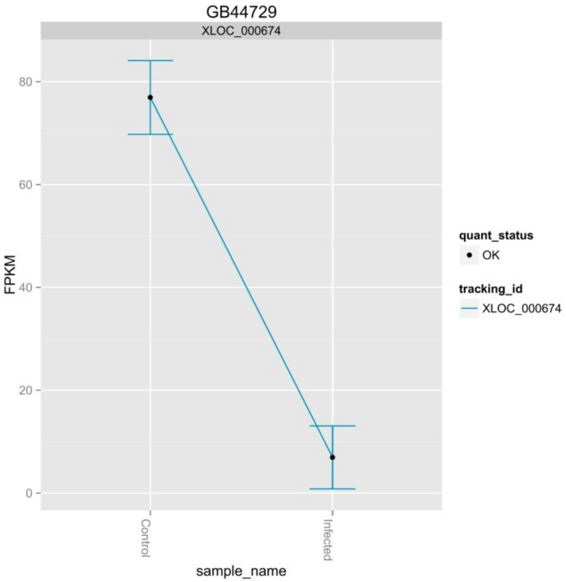
GB44729
XLOC_000674
FPKM
0
20
40
60
80
Control
Infected
sample_name
quant_status
OK
tracking_id
XLOC_000674

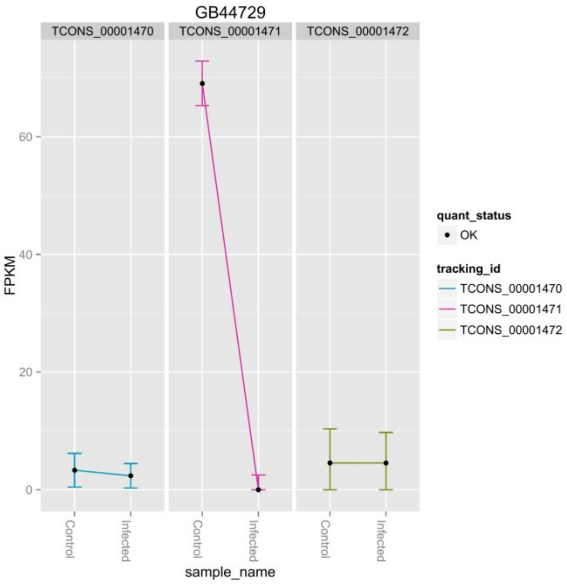
GB44729
TCONS_00001470
TCONS_00001471
TCONS_00001472
FPKM
0
20
40
60
Control
Infected
sample_name
quant_status
OK
tracking_id
TCONS_00001470
TCONS_00001471
TCONS_00001472

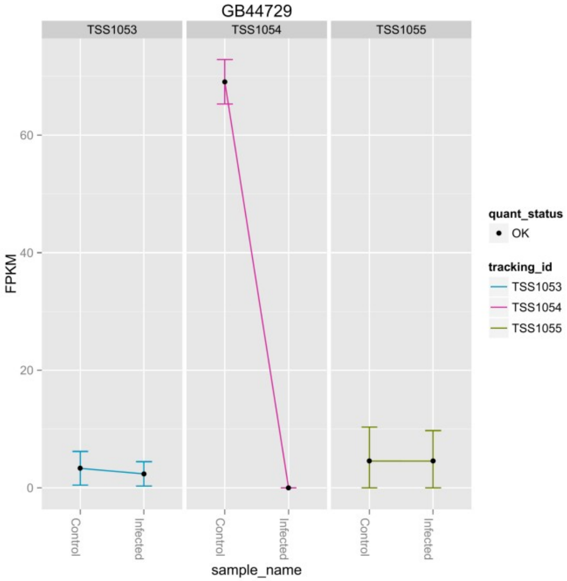
GB44729
TSS1053
TSS1054
TSS1055
FPKM
0
20
40
60
Control
Infected
sample_name
quant_status
OK
tracking_id
TSS1053
TSS1054
TSS1055

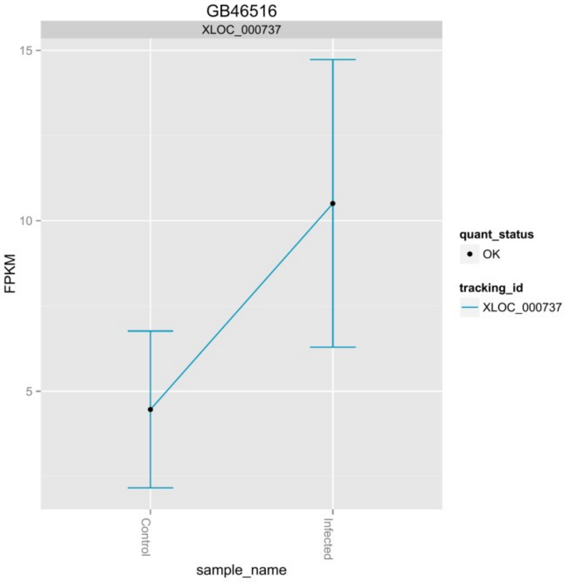
GB46516
XLOC_000737
15
10
5
FPKM
Control
Infected
sample_name
quant_status
OK
tracking_id
XLOC_000737

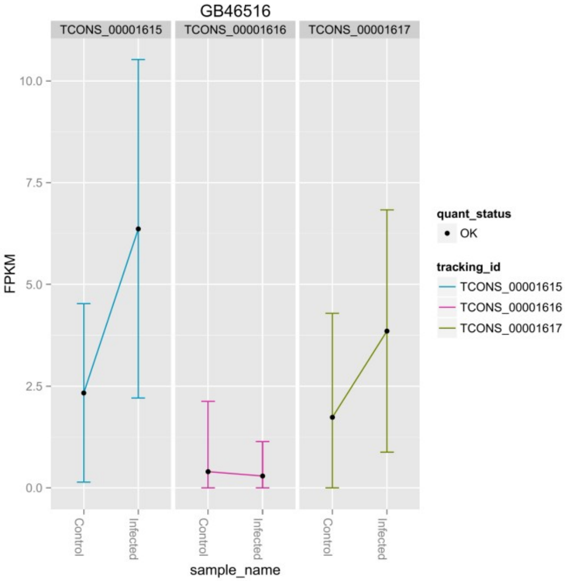
GB46516
TCONS_00001615
TCONS_00001616
TCONS_00001617
10.0
7.5
5.0
2.5
0.0
FPKM
Control
Infected
sample_name
quant_status
OK
tracking_id
TCONS_00001615
TCONS_00001616
TCONS_00001617

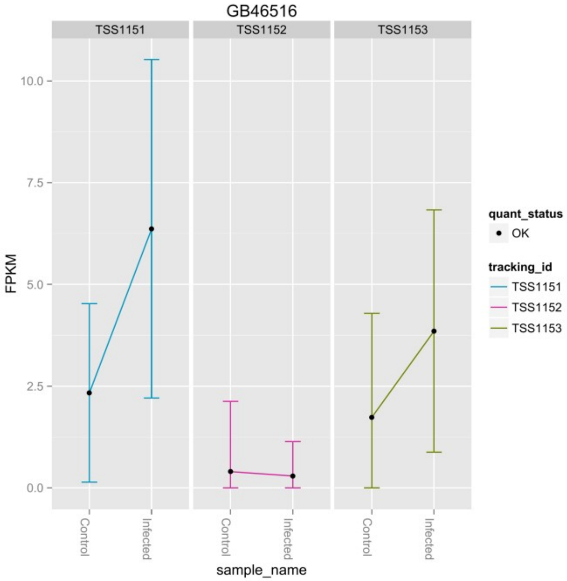
GB46516
TSS1151
TSS1152
TSS1153
10.0
7.5
5.0
2.5
0.0
FPKM
Control
Infected
sample_name
quant_status
OK
tracking_id
TSS1151
TSS1152
TSS1153

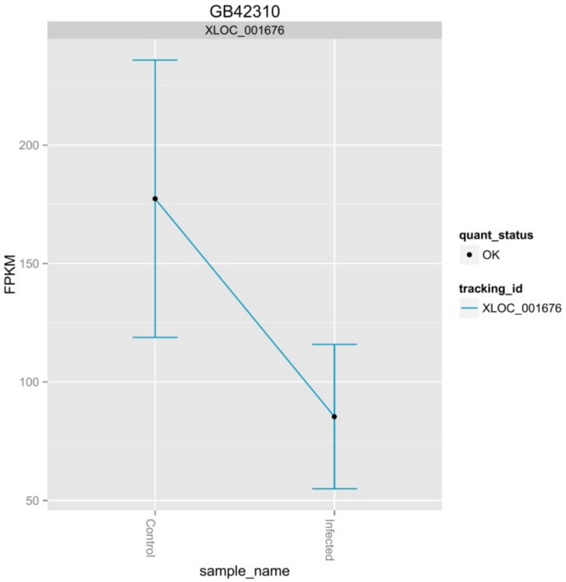
GB42310
XLOC_001676
FPKM
200
150
100
50
Control
Infected
sample_name
quant_status
OK
tracking_id
XLOC_001676

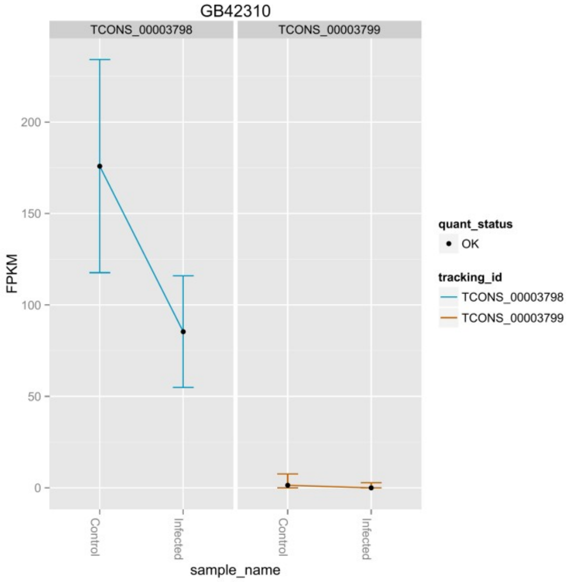
GB42310
TCONS_00003798
TCONS_00003799
FPKM
200
150
100
50
0
Control
Infected
Control
Infected
sample_name
quant_status
OK
tracking_id
TCONS_00003798
TCONS_00003799

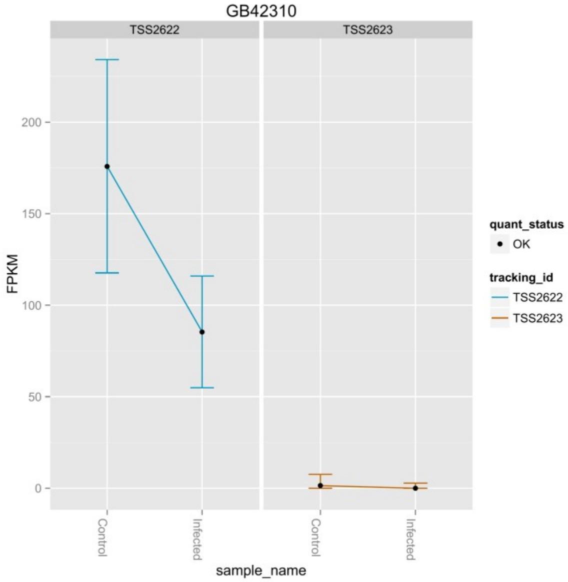
GB42310
TSS2622
TSS2623
FPKM
200
150
100
50
0
Control
Infected
Control
Infected
sample_name
quant_status
OK
tracking_id
TSS2622
TSS2623

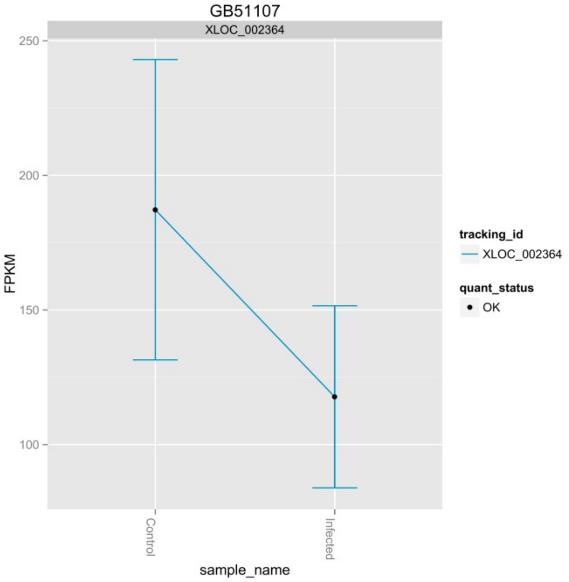
GB51107
XLOC_002364
250
200
150
100
FPKM
Control
Infected
sample_name
tracking_id
XLOC_002364
quant_status
OK

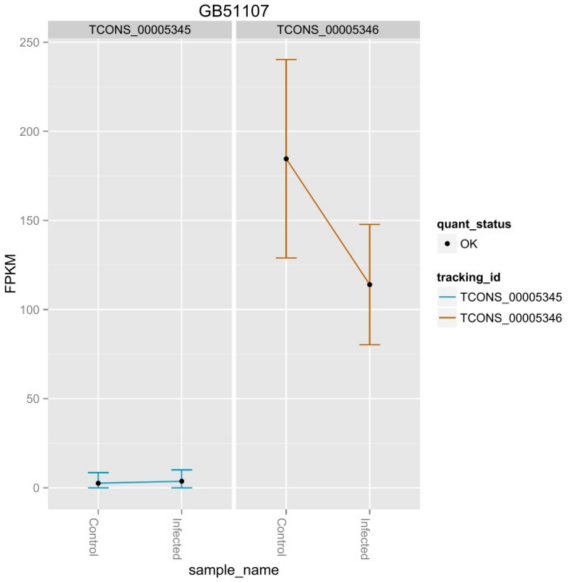
GB51107
TCONS_00005345
TCONS_00005346
250
200
150
100
50
0
FPKM
Control
Infected
Control
Infected
sample_name
quant_status
OK
tracking_id
TCONS_00005345
TCONS_00005346

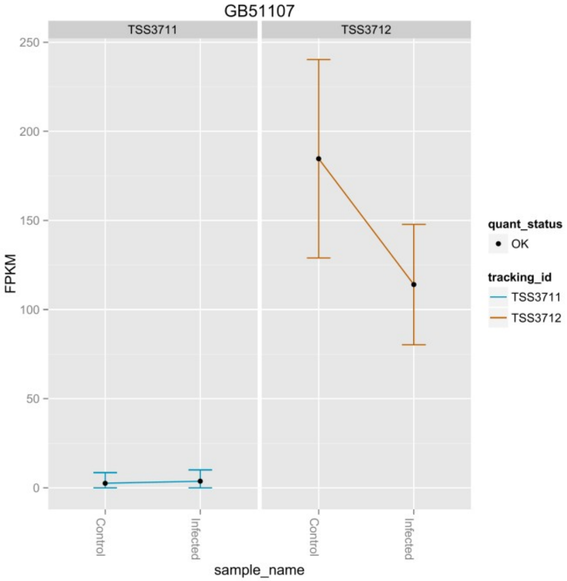
GB51107
TSS3711
TSS3712
250
200
150
100
50
0
FPKM
Control
Infected
Control
Infected
sample_name
quant_status
OK
tracking_id
TSS3711
TSS3712

Group 3:
Spliced and both transcriptionally and post-transcriptionally regulated

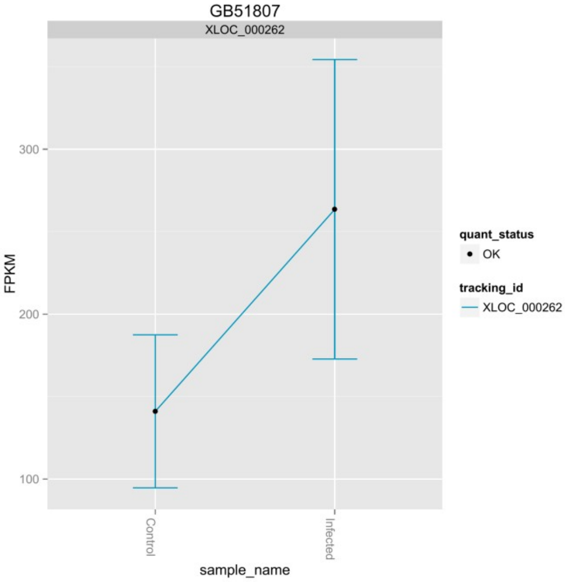
GB51807
XLOC_000262
FPKM
sample_name
Control
Infected
quant_status
OK
tracking_id
XLOC_000262

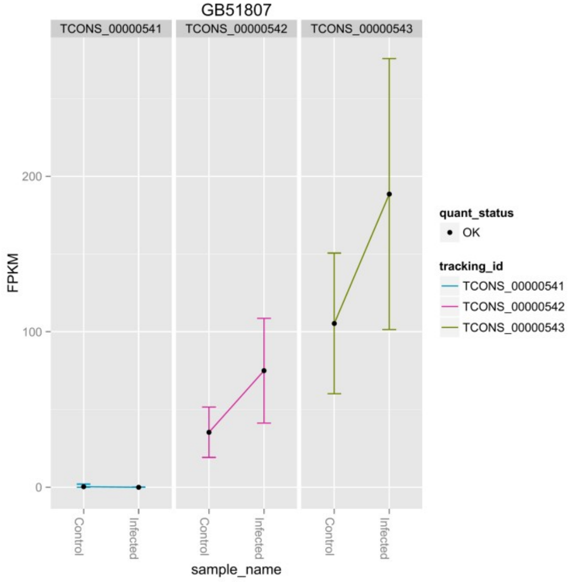
GB51807
TCONS_00000541
TCONS_00000542
TCONS_00000543
FPKM
sample_name
Control
Infected
quant_status
OK
tracking_id
TCONS_00000541
TCONS_00000542
TCONS_00000543

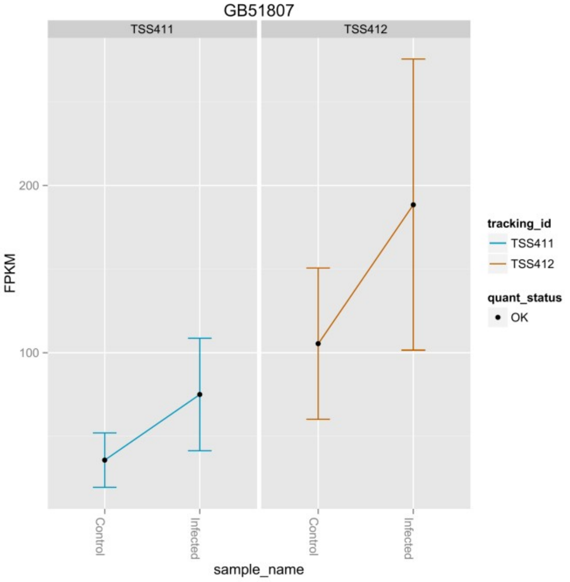
GB51807
TSS411
TSS412
FPKM
sample_name
Control
Infected
tracking_id
TSS411
TSS412
quant_status
OK

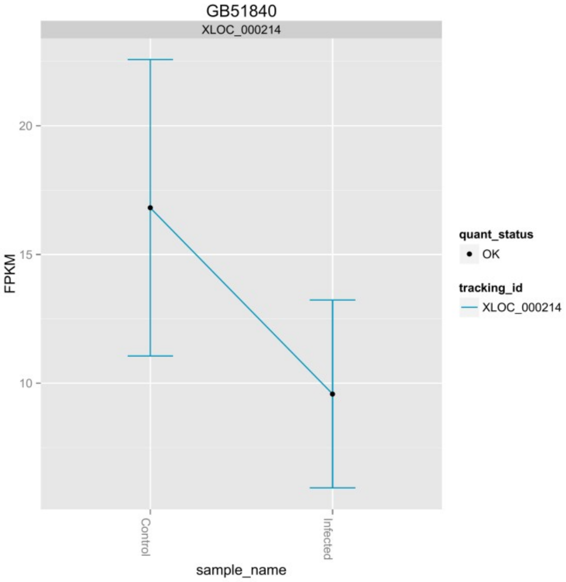
GB51840
XLOC_000214
20
15
10
FPKM
Control
Infected
sample_name
quant_status
OK
tracking_id
XLOC_000214

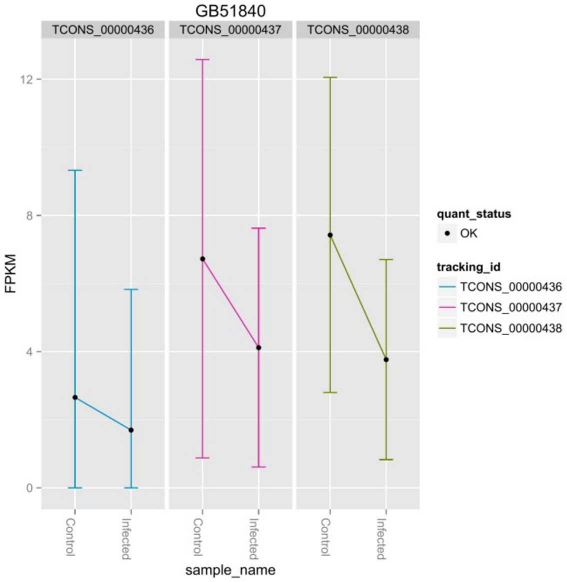
GB51840
TCONS_00000436
TCONS_00000437
TCONS_00000438
12
8
4
0
FPKM
Control
Infected
Control
Infected
Control
Infected
sample_name
quant_status
OK
tracking_id
TCONS_00000436
TCONS_00000437
TCONS_00000438

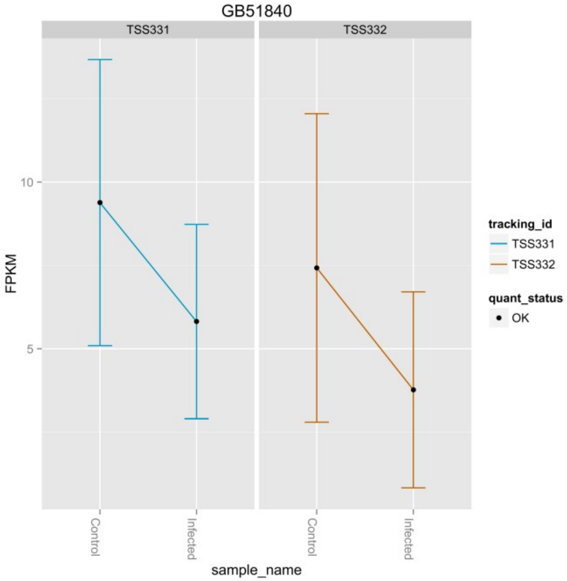
GB51840
TSS331
TSS332
10
5
FPKM
Control
Infected
Control
Infected
sample_name
tracking_id
TSS331
TSS332
quant_status
OK

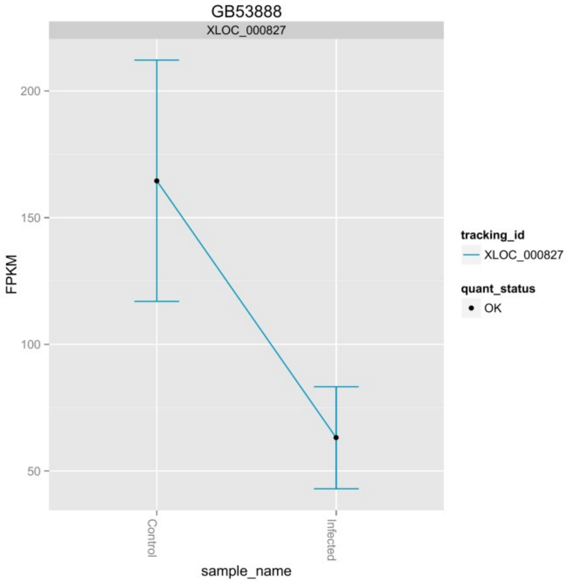
GB53888
XLOC_000827
FPKM
200
150
100
50
Control
Infected
sample_name
tracking_id
XLOC_000827
quant_status
OK

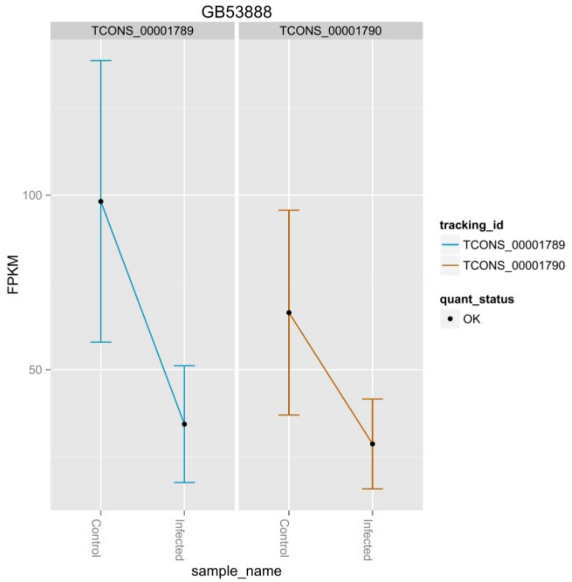
GB53888
TCONS_00001789
TCONS_00001790
FPKM
100
50
Control
Infected
Control
Infected
sample_name
tracking_id
TCONS_00001789
TCONS_00001790
quant_status
OK

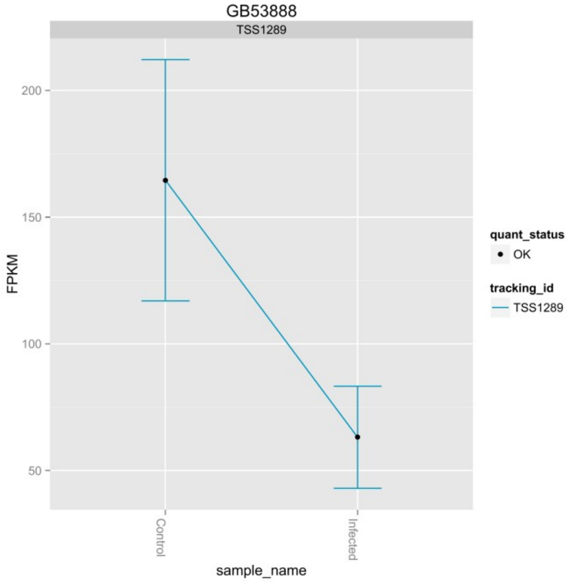
GB53888
TSS1289
FPKM
200
150
100
50
Control
Infected
sample_name
quant_status
OK
tracking_id
TSS1289

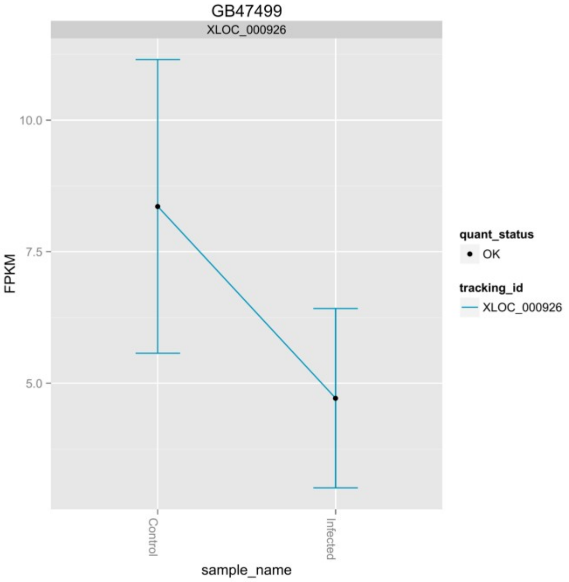
GB47499
XLOC_000926
FPKM
10.0
7.5
5.0
Control
Infected
sample_name
quant_status
OK
tracking_id
XLOC_000926

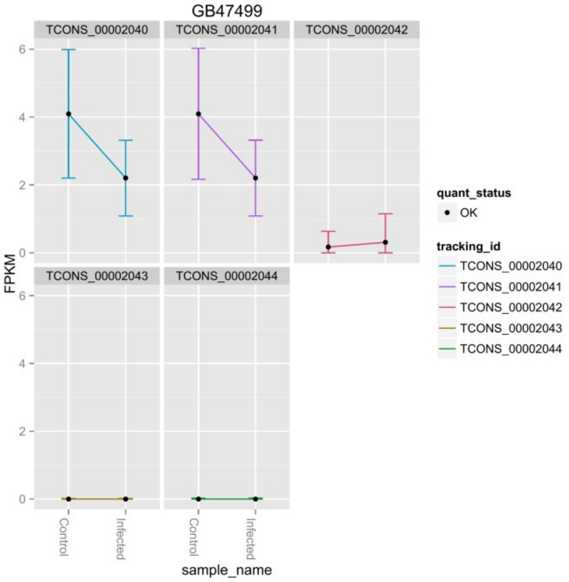
GB47499
TCONS_00002040
TCONS_00002041
TCONS_00002042
TCONS_00002043
TCONS_00002044
FPKM
6
4
2
0
Control
Infected
sample_name
quant_status
OK
tracking_id
TCONS_00002040
TCONS_00002041
TCONS_00002042
TCONS_00002043
TCONS_00002044

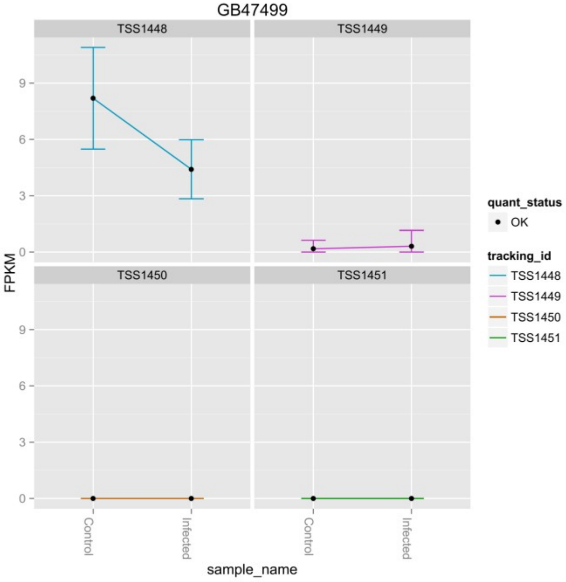
GB47499
TSS1448
TSS1449
TSS1450
TSS1451
FPKM
9
6
3
0
Control
Infected
sample_name
quant_status
OK
tracking_id
TSS1448
TSS1449
TSS1450
TSS1451

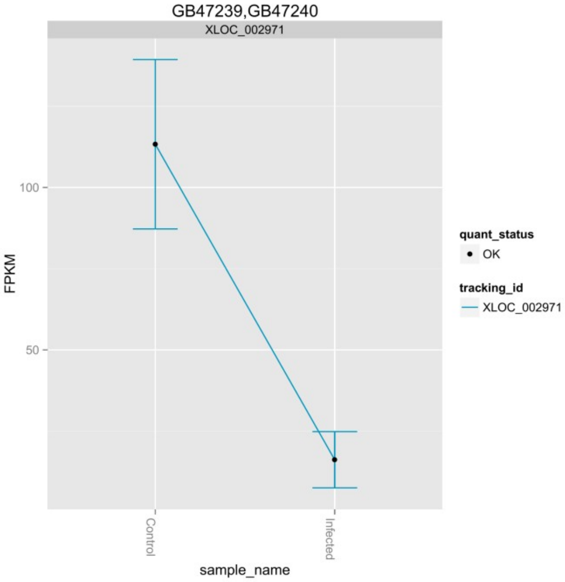
GB47239,GB47240
XLOC_002971
FPKM
100
50
Control
Infected
sample_name
quant_status
OK
tracking_id
XLOC_002971

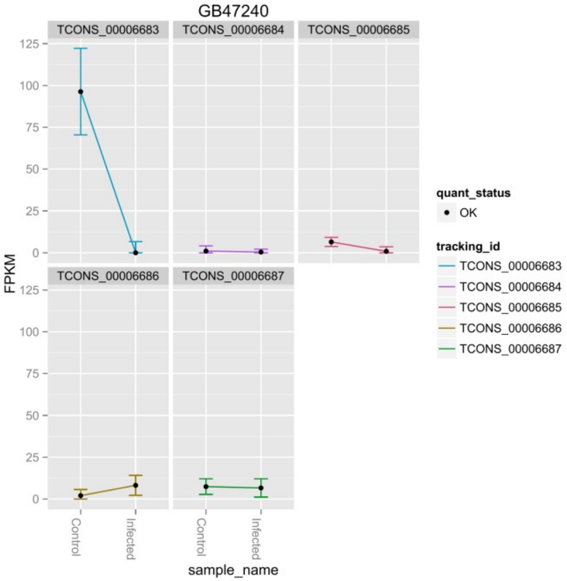
GB47240
TCONS_00006683
TCONS_00006684
TCONS_00006685
TCONS_00006686
TCONS_00006687
FPKM
125
100
75
50
25
0
Control
Infected
sample_name
quant_status
OK
tracking_id
TCONS_00006683
TCONS_00006684
TCONS_00006685
TCONS_00006686
TCONS_00006687

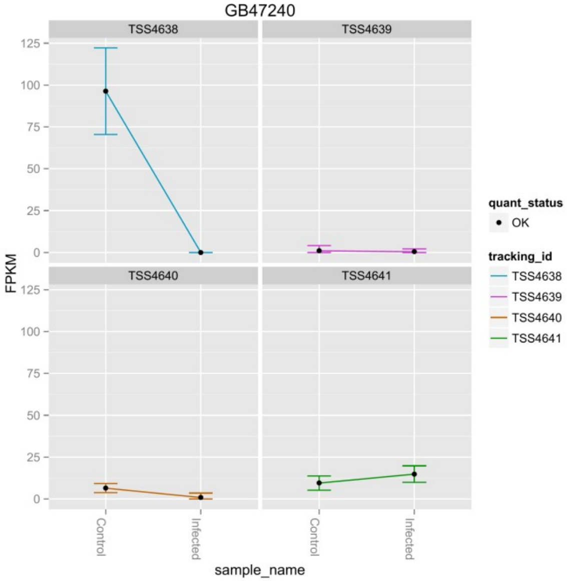
GB47240
TSS4638
TSS4639
TSS4640
TSS4641
FPKM
125
100
75
50
25
0
Control
Infected
sample_name
quant_status
OK
tracking_id
TSS4638
TSS4639
TSS4640
TSS4641

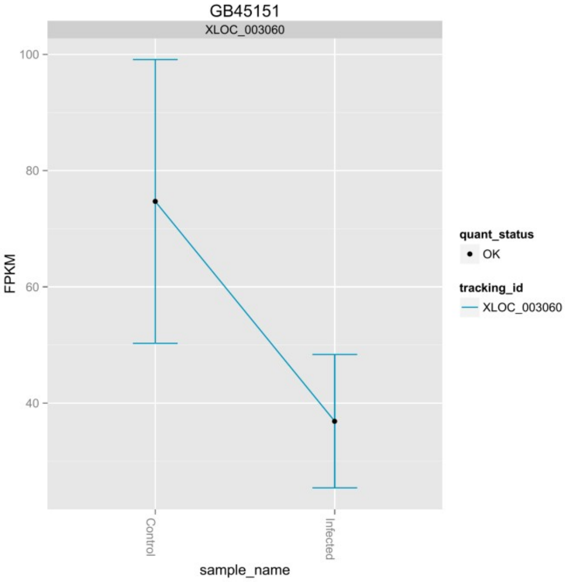
GB45151
XLOC_003060
FPKM
sample_name
Control
Infected
quant_status
OK
tracking_id
XLOC_003060

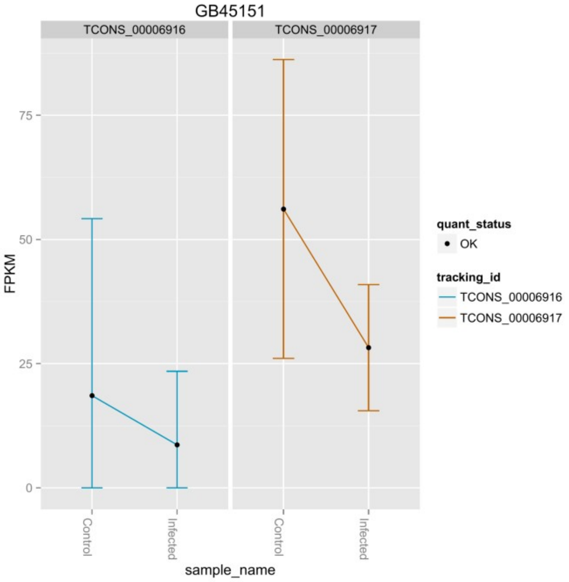
GB45151
TCONS_00006916
TCONS_00006917
FPKM
sample_name
Control
Infected
quant_status
OK
tracking_id
TCONS_00006916
TCONS_00006917

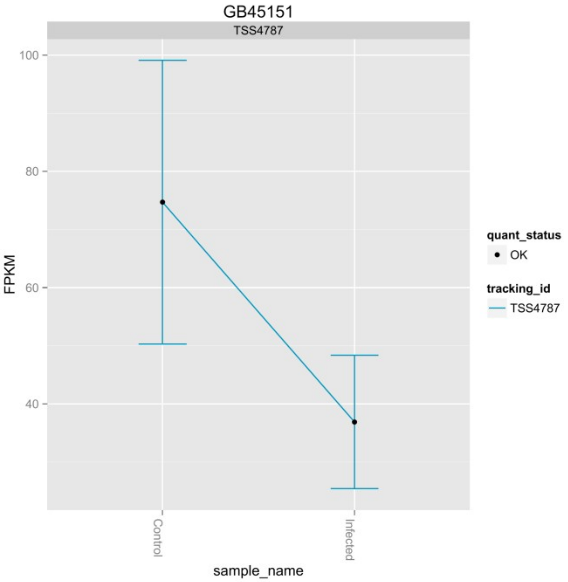
GB45151
TSS4787
FPKM
sample_name
Control
Infected
quant_status
OK
tracking_id
TSS4787
