# Supplementary figures and images for: RNA-sequence analysis of gene expression from honeybees (Apis mellifera) infected with Nosema ceranae
Source: PLoS One. 2017 Mar 28;12(3):e0173438. doi: 10.1371/journal.pone.0173438 (PMC5370102; doi:10.1371/journal.pone.0173438)

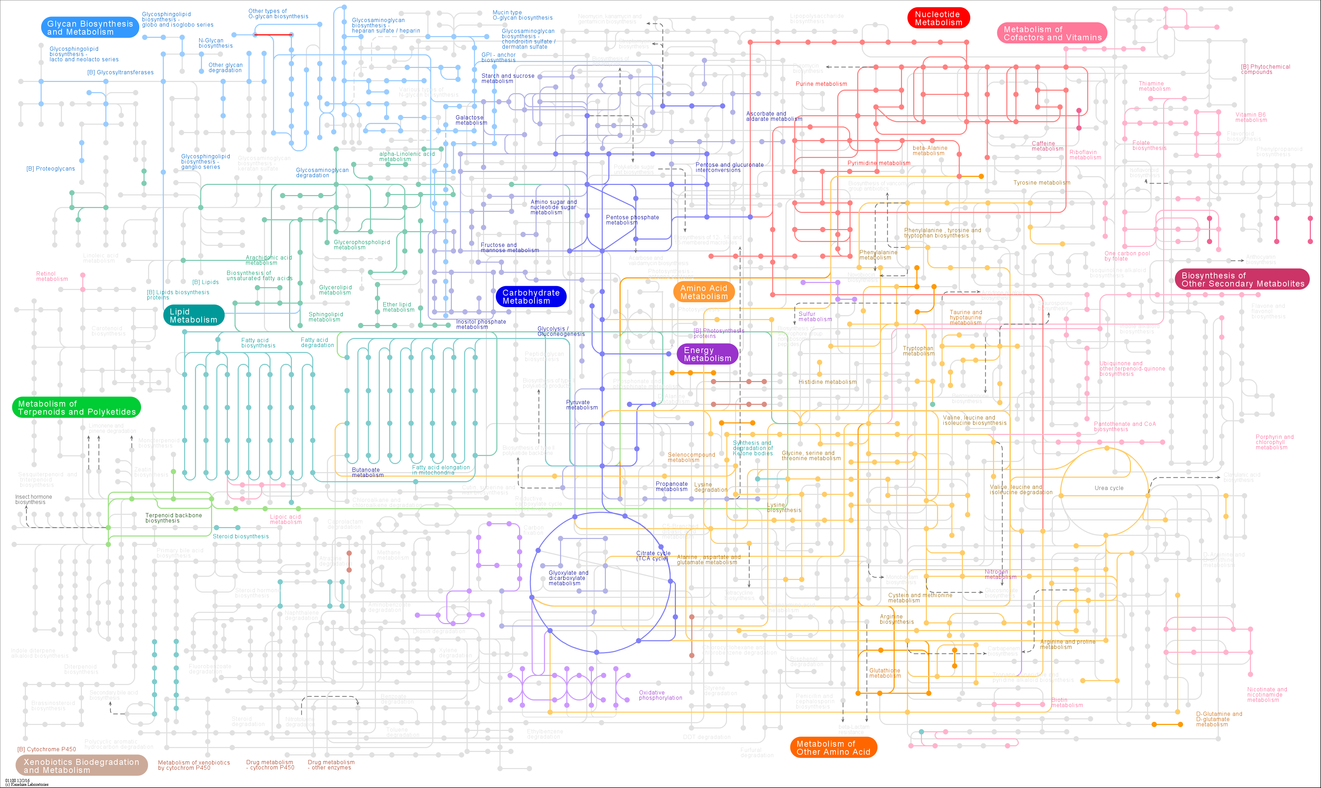

Supplement: S8 Fig — This global pathway includes mainly 'energy metabolism', 'carbohydrate metabolism', 'amino acids metabolism' and 'lipid metabolism' related genes. (TIF) [file pone.0173438.s013.tif]
